# The interferon γ pathway enhances pluripotency and X-chromosome reactivation in iPSC reprogramming

**DOI:** 10.1126/sciadv.adj8862

**Published:** 2024-08-07

**Authors:** Mercedes Barrero, Aleksey Lazarenkov, Enrique Blanco, Luis G. Palma, Anna V. López-Rubio, Moritz Bauer, Anna Bigas, Luciano Di Croce, José Luis Sardina, Bernhard Payer

**Affiliations:** ^1^ Centre for Genomic Regulation (CRG), The Barcelona Institute of Science and Technology, Dr. Aiguader 88, Barcelona 08003, Spain.; ^2^ Josep Carreras Leukemia Research Institute (IJC), Badalona 08916, Spain.; ^3^ Institut Hospital del Mar d’Investigacions Mèdiques, CIBERONC, Barcelona 08003, Spain.; ^4^ Universitat Pompeu Fabra (UPF), Barcelona 08003, Spain.; ^5^ ICREA, Passeig Lluís Companys 23, Barcelona 08010, Spain.

## Abstract

Reprogramming somatic cells into induced pluripotent stem cells (iPSCs) requires activation of the pluripotency network and resetting of the epigenome by erasing the epigenetic memory of the somatic state. In female mouse cells, a critical epigenetic reprogramming step is the reactivation of the inactive X chromosome. Despite its importance, a systematic understanding of the regulatory networks linking pluripotency and X-reactivation is missing. Here, we reveal important pathways for pluripotency acquisition and X-reactivation using a genome-wide CRISPR screen during neural precursor to iPSC reprogramming. In particular, we discover that activation of the interferon γ (IFNγ) pathway early during reprogramming accelerates pluripotency acquisition and X-reactivation. IFNγ stimulates STAT3 signaling and the pluripotency network and leads to enhanced TET-mediated DNA demethylation, which consequently boosts X-reactivation. We therefore gain a mechanistic understanding of the role of IFNγ in reprogramming and X-reactivation and provide a comprehensive resource of the molecular networks involved in these processes.

## Teaser

IFNγ treatment during reprogramming enhances X-reactivation, which depends on TET-mediated DNA demethylation.

## INTRODUCTION

A characteristic hallmark of embryonic development and pluripotency is extensive epigenetic reprogramming (*1*, *2*), for which the X chromosome is a prime example in female mammals (*3*, *4*). During female mouse development, one of the two X chromosomes switches between active and inactive states in a dynamic fashion to balance gene dosage with autosomes and XY males. The paternally inherited X chromosome is first inactivated during preimplantation development and is then subsequently reactivated in the epiblast of the late blastocyst embryo, the lineage from which all embryonic cell types emerge, and pluripotent embryonic stem cells (ESCs) are derived in culture (*5*–*7*). The erasure of epigenetic memory during X-chromosome reactivation allows afterward postimplantation epiblast cells to undergo random X-chromosome inactivation during their exit from naive pluripotency. While X-inactivation is stably maintained in somatic cells, female germ cells go through a second wave of X-chromosome reactivation before and around the time that the cells are entering meiosis and differentiating into oocytes (*8*–*12*).

Not only during female mouse development in vivo but also in cell culture in vitro, the cellular differentiation and X-chromosome states are tightly linked. While differentiated cell types are characterized by X-chromosome inactivation, female mouse pluripotent stem cells such as ESCs and induced pluripotent stem cells (iPSCs) have two active X chromosomes. On a molecular level, this can be explained by the repressive effect of the pluripotency factor network on the expression of *Xist*, the noncoding master regulator of X-inactivation (*13*–*16*), coupled with the up-regulation of *Xist* activators during differentiation of pluripotent stem cells, thereby triggering random X-chromosome inactivation (*17*–*21*). X-inactivation in mouse somatic cells is reversed during reprogramming into iPSCs by the process of X-chromosome reactivation (*22*). Previous studies have characterized the kinetics and revealed some of the regulators of X-chromosome reactivation during iPSC reprogramming (*15*, *23*–*27*); however, the full mechanisms are far from being understood.

The implementation of the CRISPR/Cas9 technology for genome and epigenome editing has allowed the generation of large-scale CRISPR screens based on the expression of pooled guide RNA (gRNA) libraries (*28*), leading to the identification of previously unknown players in pluripotency exit (*29*–*32*), maintenance (*33*–*36*), and acquisition (*37*–*39*). Furthermore, CRISPR screens in the context of the X chromosome enabled the identification of genes driving sex differences in ESCs (*40*) and *Xist* regulators (*21*, *41*). So far, most perturbation screens on the topic of pluripotency acquisition have relied on the identification of factors constituting roadblocks of the reprogramming process (*39*, *42*–*46*), as small interfering RNAs (siRNAs), short hairpin RNAs (shRNAs), or gRNAs targeting those genes would be enriched and therefore easily detected in the final iPSC population when knocked out or knocked down. However, there is a lack of genome-wide screens revealing active players in pluripotency acquisition or X-reactivation, as dropout screens rely on large cell numbers to ensure faithful shRNA/gRNA representation, which has been hard to achieve during iPSC reprogramming due to low reprogramming efficiencies. As a result, only small-scale candidate approaches have been carried out so far to identify drivers of X-chromosome reactivation during somatic cell reprogramming (*15*, *23*, *25*, *27*), and a comprehensive study of the gene regulatory networks controlling this process is missing.

To fill this gap, we performed a genome-wide CRISPR knockout (KO) screen during reprogramming of neural precursor cells (NPCs) into iPSCs, with the aim to reveal the pathways important for the process of X-chromosome reactivation. Our results show that the activation of the interferon γ (IFNγ) pathway during early stages of NPC reprogramming enhances Janus kinase (JAK)–signal transducer and activator of transcription (STAT3) signaling, pluripotency gene expression, and TET-mediated DNA demethylation, resulting in an acceleration of reprogramming kinetics and X-chromosome reactivation.

## RESULTS

### A genome-wide CRISPR KO screen reveals molecular networks involved in reprogramming and X-chromosome reactivation

To gain a comprehensive understanding of the pathways important for pluripotency acquisition and in particular for the less well-studied process of X-chromosome reactivation, we developed a cell line suitable for a genome-wide CRISPR screen during reprogramming. This approach, based on our PaX (pluripotency and X-chromosome reporter) reprogramming system (*24*), enables us to trace the pluripotency status by a *Nanog* promoter-RFP (red fluorescent protein) (P-RFP) reporter and the X-chromosome activity by an X-GFP (green fluorescent protein) reporter. Moreover, this cell line is of hybrid mouse strain origin, containing one X chromosome of *Mus castaneus* background, which is always active, and another X chromosome of *Mus musculus* background, which harbors a GFP reporter and undergoes inactivation during differentiation (due to a truncation of *Tsix*) and reactivation during reprogramming (see Materials and Methods).

**Fig. 1.**
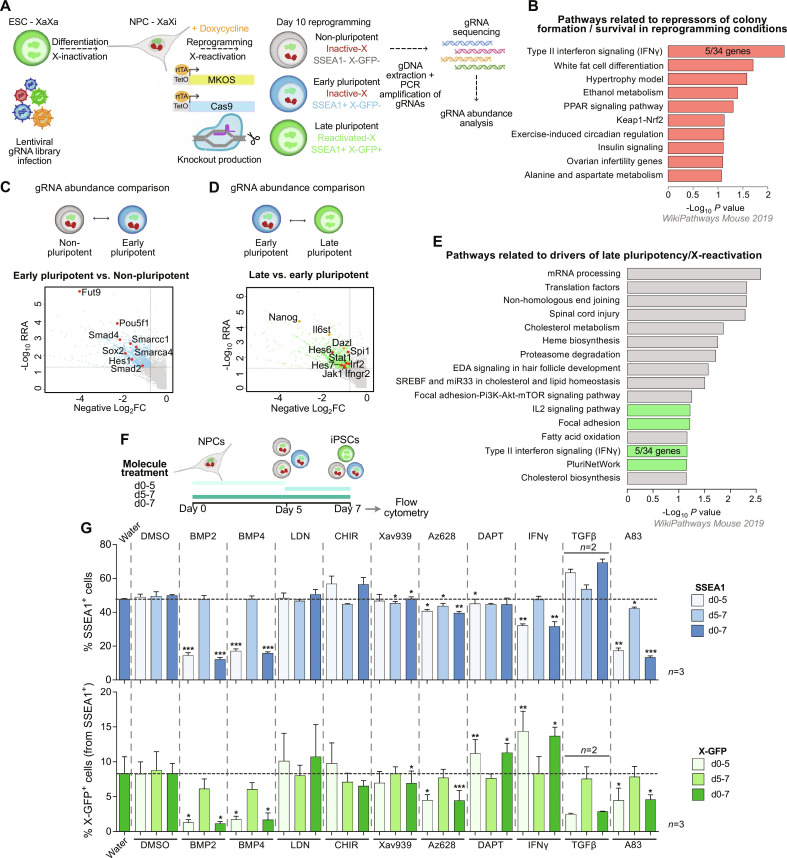
CRISPR screen reveals molecular networks involved in reprogramming and X-reactivation. (**A**) PaX-*iCas9* ESCs infected with a genome-wide lentiviral gRNA library were differentiated into NPCs, doxycycline-treated to activate reprogramming cassette and *iCas9* expression, producing KOs during reprogramming. At day 10, three populations were sorted: non-, early, and late pluripotent/X-reactivated. For these populations and NPCs, gDNA extraction, PCR amplification of gRNAs, sequencing, and gRNA abundance analysis were performed (*n* = 2 independent reprogramming rounds). (**B**) Pathways related to overrepresented genes in non-, early, and late pluripotent populations compared to NPCs. The top 250 genes from each individual comparison to NPCs ranked by RRA score with MAGeCK were used. For the IFNγ pathway, the number of genes (5 of 34) found for this GO term is indicated. (**C** and **D**) gRNA abundance comparisons of early versus nonpluripotent (C) or late versus early pluripotent populations (D) and representation of negative log_2_FC versus −log_10_ RRA (RRA cutoff = 0.05, log_2_FC cutoff = −0.75) [activators of early pluripotency in (C), where genes are highlighted in red; activators of late pluripotency/X-reactivation in (D), where pluripotency genes are shown in yellow; Notch or IFNγ signaling genes are shown in red]. (**E**) Pathways related to underrepresented genes in “late pluripotent versus early pluripotent” comparison (*n* = 1313 genes, RRA score < 0.05, log_2_FC < −0.8). Proliferation, differentiation, and metabolism pathways shown in gray; the rest of the pathways in green. (**F**) Experimental design for (G). Molecule treatments were done from day 0 to 5, day 5 to 7 or day 0 to 7; flow cytometry analysis was done at day 7. (**G**) Pathway validation: Flow cytometry analysis at day 7 of SSEA1 and X-GFP percentages (*n* = 3 reprogramming rounds; for TGFβ, *n* = 2). Data represented as mean ± SD. Statistics (paired *t* tests): where not specified = nonsignificant; *P < 0.05; **P < 0.01; ***P < 0.001.

We further modified this cell line by the introduction of a doxycycline-inducible *Cas9* (*iCas9*) transgene to mediate CRISPR-based target gene deletions (*47*). We then infected these ESCs with a gRNA library targeting all protein-coding genes in the mouse genome (*48*) and differentiated them into NPCs, leading to X-chromosome inactivation, as indicated by silencing of the X-GFP reporter (Fig. 1A). These NPCs provided the starting material for our screen. We then induced reprogramming by adding doxycycline, which activated the expression of an *MKOS* (*c-Myc*, *Klf4*, *Oct4* and *Sox2*) cassette (*49*) and *iCas9* at the same time, resulting in the production of KOs during the reprogramming process. After 10 days of doxycycline treatment, we used fluorescence-activated cell sorting (FACS) to separate three different populations, based on the SSEA1 pluripotency marker and X-GFP (indicative of a reactivated X chromosome). The three isolated populations were classified as nonpluripotent (SSEA1^−^ X-GFP^−^), early pluripotent (SSEA1^+^ X-GFP^−^), and late pluripotent (SSEA1^+^ X-GFP^+^). Although also detected, SSEA1^−^ X-GFP^+^ cells were not further analyzed in the screen as they are not represented in a faithful reprogramming and X-reactivation trajectory (*23*, *50*). The P-RFP reporter was not used in our screening strategy as it mostly mirrored the SSEA1^+^ X-GFP^+^ population and only few P-RFP^+^ X-GFP^−^ cells were detected, insufficient to maintain a proper gRNA representation for the screen. By comparing the abundance of gRNAs and their enrichment or depletion across populations, we finally identified genes with different roles for the reprogramming and X-chromosome reactivation processes.

Genes required for cell survival and normal growth (the essentialome) were depleted in all three final cell populations (nonpluripotent, early pluripotent, and late pluripotent) when compared to NPCs (fig. S1, C to I). On the other hand, overrepresented genes in the three reprogramming populations constituted repressors of iPSC colony formation and cell survival in reprogramming conditions. We found enrichment of pathways related to differentiation, metabolism, and inflammation (Fig. 1B). “Type II Interferon signaling”/IFNγ pathway showed the highest overrepresentation, suggesting a putative role of this pathway in repressing colony formation during reprogramming.

Next, to identify genes and pathways with a role early during pluripotency acquisition, we compared gRNA frequencies between the nonpluripotent and early pluripotent populations (Fig. 1C and fig. S1, J to L). As expected, we found genes with well-known roles in pluripotency such as *Smad2*, *Smad4* (related to BMP signaling), *Pou5f1*, *Sox2*, *Smarcc2*, *Smarca4* (related to pluripotency), *Hes1* (target of Notch pathway), and *Fut9* (that encodes the key enzyme necessary for SSEA1 synthesis), thereby validating our screening approach (Fig. 1C).

As our main aim was to identify previously unknown genes and pathways playing a role in naive pluripotency and in particular X-chromosome reactivation, we then focused on the comparison between the late and early pluripotent populations (Fig. 1, D and E, and fig. S1, M and N). Among the genes and pathways identified as drivers of naive pluripotency and X-reactivation, we found, as expected, the pluripotency network (with genes like *Nanog*, *Il6st*, and *Dazl*). We also identified other processes involved in cell proliferation (mRNA processing, translation), lipid metabolism, and the Notch pathway (represented by genes such as *Hes6* and *Hes7*). These have been previously described for their involvement in pluripotency acquisition but not investigated for a role in X-reactivation (*51*–*53*). Moreover, we identified the IFNγ pathway (including genes such as *Stat1*, *Jak1*, *Spi1*, *Irf2*, and *Ifngr2*) as a so far unknown putative regulator of these processes (Fig. 1, D and E).

Next, we validated our screening results by activating and/or repressing some of the identified pathways through the addition of signaling factors and small molecules during the reprogramming process focusing on potential regulators of colony formation, pluripotency acquisition, or X-chromosome reactivation. To identify an early or late contribution of the different pathways, treatment was performed at the beginning of reprogramming (from day 0 to day 5), at the end of reprogramming (from day 5 onward), or during the whole process (Fig. 1F). We tested the following pathways: bone morphogenetic protein (BMP) (activated by BMP2 and BMP4, repressed by LDN-212854), Wnt (activated by the GSK-3β inhibitor CHIR99021, repressed by the tankyrase1/2 inhibitor Xav939), mitogen-activated protein kinase (MAPK) (repressed by the pan-Raf kinase inhibitor Az628), Notch (inhibited by the γ-secretase inhibitor DAPT), IFNγ pathway (activated by IFNγ), and transforming growth factor β (TGFβ) (activated by TGFβ, repressed by the ALK5, ALK4, and ALK7 selective inhibitor A83-01). We measured the effects of the treatments on early pluripotency (SSEA1^+^) and X-chromosome reactivation (X-GFP^+^) by flow cytometry on day 7 of reprogramming, when we observed the onset of X-GFP reactivation, and therefore, the most marked change in X-chromosome status (from inactive to active) (Fig. 1G). Some molecules, such as BMP2, BMP4, or A83-01, caused a reduction of both SSEA1 and X-GFP percentages upon early or continuous treatment, indicating an early effect in the process of reprogramming. By contrast, the early or continuous treatment with IFNγ (activator of IFNγ pathway) and DAPT (inhibitor of Notch pathway) resulted in an increased percentage of X-GFP^+^ cells [around 1.76 ± 0.15 (SD)–fold and 1.37 ± 0.05–fold for early treatments, respectively] without increasing the percentage of SSEA1^+^ cells. This suggests a putative role of these molecules in the later stages of reprogramming.

Overall, our CRISPR screen validated already known pathways (BMP, MAPK, Wnt, TGFβ, and Notch signaling) (*53*–*57*) related to the different reprogramming stages. Here, we focused on the previously unidentified IFNγ signaling pathway, which plays contrasting roles during the iPSC reprogramming process: early on as a repressor of colony formation (Fig. 1B), but subsequently as a driver of late pluripotency and X-chromosome reactivation (Fig. 1, D and E). As IFNγ induced the highest increase in X-chromosome reactivation efficiency in the validation experiments (Fig. 1G) and has never been implicated in these processes before, we therefore from now on focused on characterizing its mechanism of action.

### IFNγ signaling modulates colony formation and X-chromosome reactivation during iPSC reprogramming

**Fig. 2.**
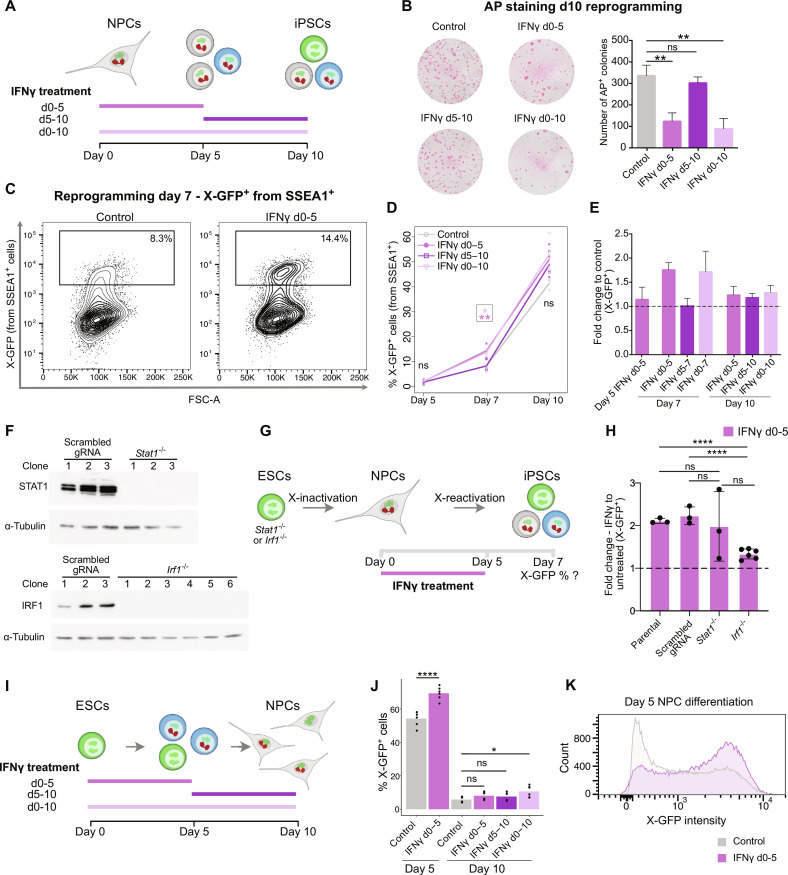
IFNγ modulates colony formation and X-reactivation during reprogramming. (**A**) For (B) to (E), IFNγ was added from day 0 to 5, day 5 to 10, day 0 to 10 (*n* = 3 reprogramming rounds). (**B**) AP stainings on day 10 of reprogramming (*n* = 3 for control, *n* = 4 for IFNγ treatments). Statistics (unpaired *t* tests): ns = nonsignificant; **P < 0.01. Error bars represent SD. (**C**) Flow cytometry plots of X-GFP^+^ (from SSEA1^+^) cells (gate) in control and IFNγ treatment (day 0 to 5) at day 7. (**D**) Flow cytometry analysis of X-GFP^+^ cells on reprogramming days 5, 7, and 10. Statistics (paired *t* tests): *P < 0.05; **P < 0.01. (**E**) Fold change of X-GFP percentages compared to each control. Error bars represent SD [calculations based on percentages in (D)]. (**F**) Western blot of STAT1 and IRF1 for three scrambled gRNA and three *Stat1*^−/−^ clones (top) or six *Irf1*^−/−^ clones (bottom). Loading control: ɑ-tubulin. (**G**) For (H), NPCs derived from three *Stat1*^−/−^, six *Irf1*^−/−^, three parental, and three scrambled gRNA ESC clones were reprogrammed into iPSCs ± IFNγ (day 0 to 5). X-GFP percentages measured by flow cytometry at day 7. (**H**) Fold change of X-GFP^+^ percentage (IFNγ versus untreated controls) on day 7 measured by flow cytometry. Clones with the same genotype listed in (G) are grouped; dots represent the mean of three technical replicates for each clone. Statistics (unpaired *t* tests): ****P < 0.0001. Error bars represent SD. (**I**) For (J) and (K), IFNγ treatment was done during NPC differentiation (day 0 to 5, day 5 to 10, and day 0 to 10) (*n* = 6 independent replicates). (**J**) X-GFP percentage on days 5 and 10 of NPC differentiation measured by flow cytometry. Statistics (paired *t* tests): *P < 0.05; ****P < 0.0001. (**K**) Flow cytometry histogram of X-GFP intensity in representative samples of control and IFNγ-treated day 5 NPCs.

In our CRISPR screen, the IFNγ pathway showed up as a putative repressor of iPSC colony formation and potential driver of X-chromosome reactivation. We explored the role of IFNγ signaling in these two scenarios after IFNγ treatment at different time points: early (day 0 to 5), late (day 5 to 10), and continuous (day 0 to 10) (Fig. 2A). To further investigate the implication of the IFNγ pathway activation in iPSC colony formation, we performed alkaline phosphatase (AP) staining after 10 days of reprogramming (Fig. 2B) upon different timings of IFNγ treatment. The early (day 0 to 5) and continuous (day 0 to 10) treatments induced a decrease in AP-positive colony number, validating the role of IFNγ signaling as a repressor of colony formation, while the late treatment (day 5 to 10) did not have any effect. This phenotype could be related to a slight increase in apoptosis observed after 48 hours from IFNγ treatment during the onset of reprogramming induction (fig. S2A). Next, we tested X-chromosome reactivation efficiency by measuring the percentage of X-GFP^+^ cells at days 5, 7, and 10 of reprogramming (Fig. 2, C to E). At day 7, the early and continuous treatment with IFNγ resulted in a significant increase in cells undergoing X-GFP reactivation (Fig. 2D), with average fold changes of 1.76 ± 0.15 and 1.71 ± 0.42 to the control, respectively (Fig. 2E), while the differences between IFNγ-treated and control samples were less prominent at day 10 (Fig. 2, D and E), suggesting that early IFNγ treatment accelerates X-reactivation. Moreover, by reseeding SSEA1^+^ X-GFP^−^ cells at day 7, we observed that they had the capacity to achieve X-reactivation late in reprogramming (day 12) at similar levels (fig. S3A) and form pluripotent AP-positive colonies at 3.3 ± 0.3–fold higher numbers in the IFNγ-treated condition than in controls (fig. S3B).

IFNγ signaling induces the activation of the transcription factors STAT1 and IRF1 (interferon regulatory factor 1), which in turn activate the expression of IFNγ-response genes. To determine the speed of activation of IFNγ target genes upon treatment, we analyzed the expression of *Irf1* and *Gbp2* by quantitative reverse transcription polymerase chain reaction (qRT-PCR) in NPCs during the first 9 hours of reprogramming induction. A strong increase in the expression of the IFNγ pathway genes *Irf1* and *Gbp2* was observed already after 3 to 6 hours of treatment (fig. S2B). Moreover, we detected an increased expression of STAT1 and phospho-STAT1 at the protein level at days 2 and 5 of reprogramming in the IFNγ-treated cells compared to the control, indicating activation of the pathway during reprogramming upon IFNγ treatment (fig. S2, C to E). To shed light on the mechanism behind the increased X-chromosome reactivation efficiency upon IFNγ treatment, we generated *Stat1*^−/−^ and *Irf1*^−/−^ ESC lines (Fig. 2F), induced reprogramming in NPCs generated from them with and without IFNγ treatment from day 0 to 5, and analyzed the percentages of cells undergoing X-GFP reactivation at day 7 of reprogramming by flow cytometry (Fig. 2, G and H, and fig. S2F). As in our previous experiments, IFNγ treatment resulted in an around 2-fold increase in the percentage of X-GFP^+^ cells in the parental (2.11 ± 0.06–fold) and scrambled gRNA (2.23 ± 0.2–fold) controls compared to untreated cells. In the *Stat1*^−/−^ cell lines, IFNγ treatment still induced an increase in X-GFP reactivation efficiency comparable to the controls in two of three clones (1.98± 0.82–fold), suggesting that STAT1 is unlikely to be the main responsible downstream factor for the observed phenotype. By contrast, all six *Irf1*^−/−^ clones analyzed showed less of an increase in X-GFP reactivation compared to the parental or scrambled control clones in response to IFNγ treatment (IFNγ versus control X-GFP fold changes varied from 1.18 to 1.46, *P* < 0.0001, average = 1.33 ± 0.12–fold). Together, these data suggest that IRF1 is a mediator of IFNγ signaling responsible for the increased efficiency of X-GFP reactivation observed upon IFNγ treatment.

Next, we explored the effects of IFNγ treatment in other contexts of reprogramming and cell differentiation. In the MEF (mouse embryonic fibroblast) reprogramming system (fig. S4), IFNγ caused a reduction in colony number as well but did not enhance X-reactivation, indicating that the IFNγ-mediated increase in X-reactivation efficiency is reprogramming context specific. Then, we wanted to know if IFNγ has the opposite effect on ESC differentiation into NPCs than during NPC reprogramming. For this, we treated cells undergoing differentiation with IFNγ from day 0 to 5, day 5 to 10, or throughout the whole process (Fig. 2I) and assessed the percentages of SSEA1^+^ and X-GFP^+^ cells by flow cytometry on days 5 and 10 (fig. S2G and Fig. 2, J and K). At day 5 of differentiation, no changes in SSEA1 percentage were detected between the control and the IFNγ-treated samples (fig. S2G), while the X-GFP percentage was elevated in the IFNγ-treated cells at day 5 of differentiation compared to the control (Fig. 2, J and K). In contrast, on day 10 of differentiation, substantial changes were no longer detected in X-GFP expression between control and treated samples, with a significant but relatively minor increase in the X-GFP percentage in IFNγ-treated samples from day 0 to 10 (Fig. 2J). Similarly, a significant albeit small increase in percentage of SSEA1^+^ cells was detected at day 10 of differentiation in IFNγ-treated samples from day 0 to 5 and day 0 to 10 (fig. S2G). These data indicate that IFNγ treatment during differentiation delays X-chromosome inactivation, which is opposite to its observed role in NPC to iPSC reprogramming, where IFNγ accelerates X-chromosome reactivation instead.

### IFNγ pathway activation accelerates the reprogramming process

**Fig. 3.**
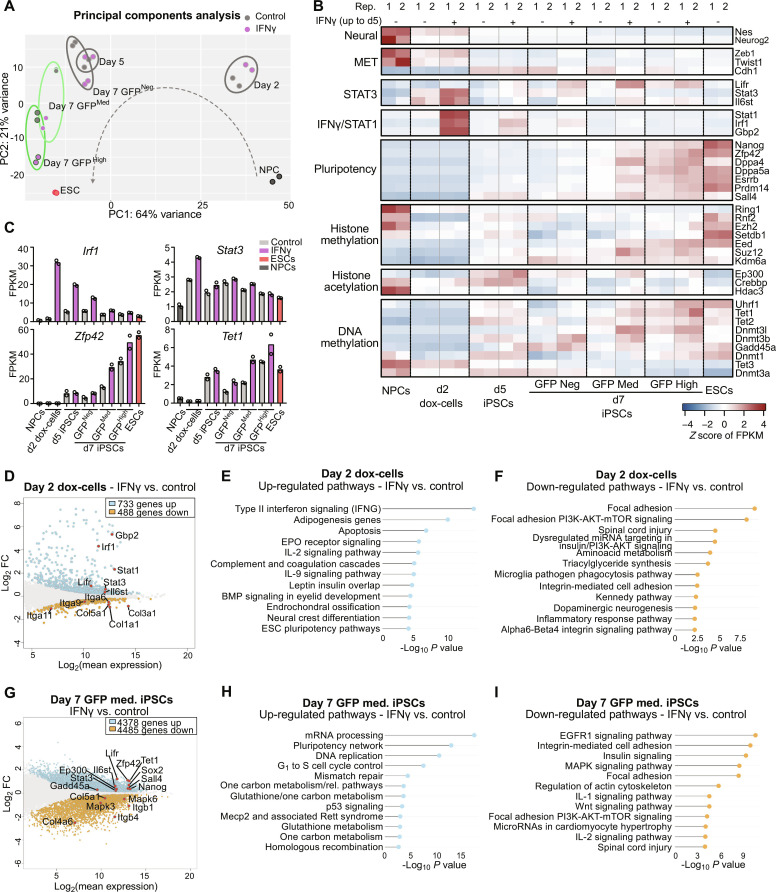
IFNγ pathway activation accelerates the reprogramming process. (**A**) PCA of RNA-seq of NPCs, day 2, day 5, and day 7 reprogramming populations, and ESCs, in control and IFNγ treatment (day 0 to 5), representing the top 500 most variable genes. (**B**) Heatmap representing expression (*z* score of FPKM) of neural genes, mesenchymal-to-epithelial transition (MET) genes, pluripotency genes, STAT3- and IFNγ/STAT1-related genes, histone methylation genes, histone acetylation genes, and DNA methylation genes. (**C**) Expression (FPKM) of selected genes (*Irf1, Stat3, Zfp42/Rex1*, and *Tet1*) in NPCs, ESCs, and day 2, day 5, and day 7 reprogramming populations ± IFNγ treatment (two RNA-seq replicates shown). (**D**) MA plot (log_2_FC vs log_2_ mean expression) displaying transcriptomic changes of IFNγ versus control day 2 reprogramming cells (adjusted *P* = 0.1). Up-regulated genes are highlighted in light blue; down-regulated genes are highlighted in orange. Selected genes are shown with points in red. (**E** and **F**) Up-regulated (E) and down-regulated (F) pathways in IFNγ versus control day 2 reprogramming cells (WikiPathways Mouse 2019) (adjusted *P* = 0.1). (**G**) MA plot displaying transcriptomic changes of IFNγ versus control day 7 X-GFP medium iPSCs (adjusted *P* = 0.1). Up-regulated genes are highlighted in light blue; down-regulated genes are highlighted in orange. Selected genes are shown with points in red. (**H** and **I**) Up-regulated (H) and down-regulated (I) pathways in IFNγ versus control day 7 X-GFP medium iPSCs (WikiPathways Mouse 2019) (adjusted *P* = 0.1).

To gain insight into the changes induced by early IFNγ treatment (day 0 to 5), we performed transcriptomic analyses of FACS-sorted cells at days 2, 5 (SSEA1^+^), and 7 (SSEA1^+^/X-GFP–negative, X-GFP–medium, and X-GFP–high) (fig. S5A) of reprogramming and compared them to untreated cells, and NPCs and ESCs as fully differentiated and pluripotent cell types, respectively. Principal components analysis (PCA) revealed a strong similarity between control and IFNγ-treated cells at days 2 and 5 of reprogramming (Fig. 3A). However, at day 7, IFNγ-treated iPSCs showed an accelerated reprogramming kinetics compared to the control, clustering closer to the ESCs. This trend was also observed when only autosomal genes were taken into account (fig. S5B).

Next, we explored the expression of several genes involved in the reprogramming process (Fig. 3, B and C, and fig. S5D). This analysis indicated an early activation of the IFNγ-related genes *Stat1*, *Irf1*, and *Gbp2* upon treatment, which peaked at day 2 of reprogramming. Of note, IFNγ-treated iPSCs also showed a higher expression of some genes related to the LIF/STAT3 pathway (*Stat3*, *Lifr*, *Il6st*), which is involved in the acquisition of pluripotency (*58*–*60*). Pluripotency genes showed a higher expression in the IFNγ-treated samples compared to the control, especially in the X-GFP–medium population, which is undergoing X-reactivation. Examples of these naive pluripotency genes are *Nanog*, *Zfp42/Rex1*, *Dppa4*, *Dppa5a*, *Esrrb*, *Prdm14*, and *Sall4*. This supports a more advanced reprogramming in the IFNγ-treated samples, consistent with what we have observed in the PCA (Fig. 3A), and is in line with studies showing an involvement of naive pluripotency factors such as *Nanog* and *Prdm14* in X-chromosome reactivation (*10*, *15*, *61*). We then focused on genes related to DNA demethylation dynamics, as demethylation of X-linked gene promoters is a key step in X-chromosome reactivation (*23*, *25*, *62*). While we did not observe differences in the expression of *Tet2* and *Tet3*, we saw a higher expression of *Tet1* and *Gadd45a* from day 5 onward in the IFNγ-treated cells in comparison to the control (Fig. 3, B and C). *Tet1* has previously been shown to be up-regulated during iPSC reprogramming and to demethylate and reactivate pluripotency genes (*63*). GADD45A is a member of the base excision repair pathway that was found to interact with TET1, promoting its activity and enhancing DNA demethylation (*64*). Thus, the up-regulation of these genes upon IFNγ treatment could potentially contribute to DNA demethylation, leading to a more rapid cell fate transition and more efficient or faster X-chromosome reactivation.

We then performed differential expression analysis between control and IFNγ-treated cells at days 2, 5, and 7 (Fig. 3, D to I; fig. S5, E to K; and table S3). As expected, day 2 and 5 cells undergoing reprogramming showed an up-regulation of IFNγ signaling pathway signature genes, including *Gbp2*, *Stat1*, and *Irf1* in the IFNγ-treated cells (Fig. 3, D and E, and fig. S5, F and G). Additionally, at day 2, we observed an activation of other inflammation pathways, like complement and coagulation cascades, interleukin-2 (IL-2), IL-9, and also apoptosis (Fig. 3E), fitting with the increased percentage of annexin V–positive cells observed upon IFNγ treatment early during reprogramming (fig. S2A). As mentioned above, some genes from the pluripotency-related STAT3 pathway showed an increased expression early upon IFNγ treatment, like *Lifr*, *Stat3*, and *Il6st* (Fig. 3, B to D), in line with the higher expression of genes related to pluripotency and/or DNA demethylation detected at day 5 (*Esrrb*, *Lifr*, *Tet1*, and *Gadd45a*) (Fig. 3, B and C, and fig. S5, D, F, and G). Focusing on the down-regulated pathways and genes upon IFNγ treatment, we found a reduction of focal adhesion genes on both days 2 and 5 (Fig. 3F and fig. S5H), predominantly represented by integrins and collagens (*Itga9*, *Col1a1*, *Col3a1*, *Col5a1*) (Fig. 3D and fig. S5F). Integrin-mediated cell adhesion has been shown to have an impact in colony number in reprogramming (*43*). Thus, the decreased expression of focal adhesion genes, together with the increased apoptosis observed upon IFNγ treatment (fig. S2A), could explain the lower colony number in the IFNγ-treated samples (Fig. 2B).

Next, we compared the transcriptome of day 7 IFNγ-treated X-GFP–negative, X-GFP–medium, and X-GFP–high populations with their respective untreated controls. Pairwise comparisons between these populations showed very similar results (Fig. 3, G to I; fig. S5, I to K; and table S3). In all cases, the early treatment with IFNγ showed an up-regulation of proliferation pathways (mRNA processing, G_1_ to S cell cycle control), metabolism-related pathways, and the pluripotency network, including genes such as *Nanog* and *Zfp42/Rex1* (Fig. 3, G and H, and fig. S5, I and J). Other genes found to be up-regulated in the IFNγ-treated iPSCs were the genes involved in DNA demethylation *Tet1* and *Gadd45a* (Fig. 3G and fig. S5I), as also observed at day 5 (fig. S5F). In addition, in the X-GFP–negative and X-GFP–medium populations, several genes belonging to the LIF-STAT3 pathway were found to be up-regulated, such as *Il6st*, *Lifr*, and *Stat3* (Fig. 3G and fig. S5I), consistent with the results of day 2 (Fig. 3D). Among the common down-regulated pathways in the IFNγ-treated day 7 iPSCs, we found the epidermal growth factor receptor 1 (EGFR1) signaling and MAPK pathways (that are linked to differentiation) (*65*, *66*), inflammation pathways (IL-1 and IL-2), and also focal adhesion (Fig. 3I and fig. S5K), consistent with our previous results on days 2 and 5. Overall, our transcriptomic analysis revealed that IFNγ early treatment accelerated the reprogramming process, as reflected by increased expression of STAT3-, DNA demethylation–, and pluripotency-related genes.

### IFNγ treatment during reprogramming enhances JAK-STAT3 signaling, pluripotency gene expression, and X-chromosome reactivation

**Fig. 4.**
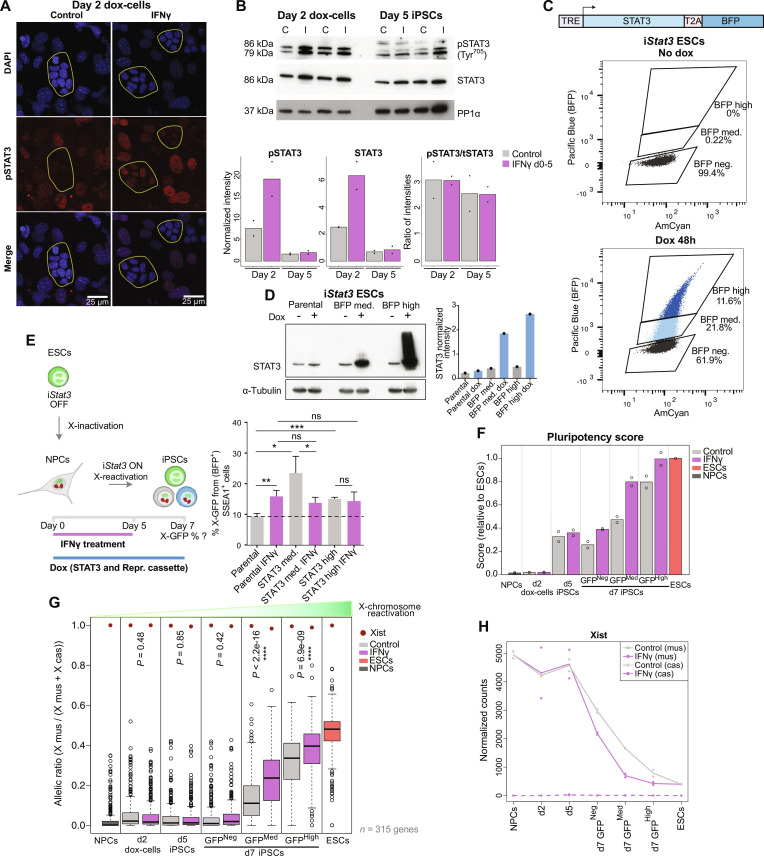
IFNγ treatment during reprogramming enhances JAK-STAT3 signaling, pluripotency gene expression, and X-reactivation. (**A**) Immunofluorescence of pSTAT3 (Tyr^705^) on day 2 doxycycline (dox)–treated cells ± IFNγ. Scale bar, 25 μm. Z projections of maximum intensity from six stacks are shown for all channels. Outlines highlight colonies of cells undergoing reprogramming (smaller nuclei, tight aggregation). (**B**) Western blotting of STAT3 and pSTAT3 (Tyr^705^) on days 2 and 5 of reprogramming ± IFNγ (C, control; I, IFNγ; loading control: PP1α). Normalized intensities to loading control for pSTAT3 and total STAT3, and pSTAT3/total STAT3 intensity ratios are shown. (**C**) STAT3 overexpression construct design and flow cytometry plots showing STAT3-BFP activation in untreated and 48-hour doxycycline–treated ESCs. (**D**) Western blotting of STAT3 in parental and STAT3-BFP–medium/high ESC sorted populations after 48 hours of doxycycline treatment, and intensities normalized to loading control (α-tubulin). (**E**) NPCs derived from parental, BFP-medium, and BFP-high ESCs (7 days of doxycycline withdrawal) were reprogrammed (± IFNγ day 0 to 5). Flow cytometry analysis of X-GFP expression [from (BFP^+^) SSEA1^+^ cells] was performed on day 7 of reprogramming (*n* = 3 technical replicates). Statistics (unpaired t tests): *P < 0.05; **P < 0.01; ***P < 0.001. Error bars represent SD. (**F**) Pluripotency score (relative to ESCs) in the two RNA-seq replicates during reprogramming, calculated from the expression levels of *Nanog*, *Zfp42/Rex1*, *Dppa4*, *Dppa5a*, *Esrrb*, *Prdm14*, and *Sall4*. (**G**) Allelic ratio of 315 genes expressed from X cas on NPCs, ESCs, and day 2, day 5, and day 7 reprogramming populations ± IFNγ treatment. Statistics (unpaired *t* tests): not specified = nonsignificant; ****P < 0.0001. (**H**) Expression (normalized counts) of *Xist* from X mus and X cas in NPCs, ESCs, and day 2, day 5, and day 7 reprogramming populations ± IFNγ treatment (two RNA-seq replicates shown).

To explore if the increased expression of LIF-STAT3 signaling–related genes (Fig. 3, B to D) correlated with a higher activation of the pathway, we determined the levels of phosphorylated (Tyr^705^) STAT3 protein by immunofluorescence in control and IFNγ-treated cells at day 2 of reprogramming (Fig. 4A). Although we observed nuclear staining of phospho-STAT3 in the control samples, the signal was more intense in the IFNγ-treated cells, indicating a higher activation of the pathway upon IFNγ treatment. We confirmed this quantitatively by Western blot, which showed increased levels of both total (2.52 ± 0.6–fold) and phospho-STAT3 (2.79 ± 1.7–fold) in the IFNγ-treated day 2 reprogramming cells compared to the control (Fig. 4B). However, this effect was no longer observed in IFNγ-treated day 5 iPSCs (Fig. 4B), indicating that IFNγ-mediated increase of JAK-STAT3 signaling activation occurs only transiently early during reprogramming. To examine if IFNγ signaling enhances X-reactivation via increasing STAT3 expression, we generated doxycycline-inducible *Stat3*-*BFP* overexpression ESC pools with medium or high expression of the transgene after 48 hours of doxycycline treatment (Fig. 4C). We confirmed STAT3 overexpression at medium and high levels in ESCs by Western blot (Fig. 4D). Then, we treated NPCs differentiated from parental and STAT3 [blue fluorescent protein (BFP)] medium and high cells with doxycycline to induce the expression of the reprogramming cassette and the *Stat3*-*BFP* transgene, in the presence or absence of IFNγ (day 0 to 5), and analyzed colony number (fig. S6A) and X-GFP reactivation from (BFP^+^) SSEA1^+^ cells by flow cytometry at day 7 of reprogramming (Fig. 4E). IFNγ treatment led to reduced colony numbers both with and without induction of the STAT3 transgene. On the other hand, STAT3 overexpression resulted in increased levels of X-GFP reactivation compared to the parental control, which were not further enhanced upon IFNγ treatment. We also wanted to test the effect of IFNγ during reprogramming of *Stat3*^−/−^ NPCs, although these could not be obtained due to precocious differentiation of the *Stat3*^−/−^ ESCs (fig. S7). In summary, the fact that IFNγ did not further enhance X-GFP reactivation upon STAT3 overexpression suggests that STAT3 is a downstream mediator of IFNγ signaling in this context.

To further characterize the effect of IFNγ on reprogramming speed, we next calculated a pluripotency score based on the mRNA expression of selected naive pluripotency genes for each time point in control and IFNγ-treated iPSCs (Fig. 4F). This score was higher in all day 7 IFNγ-treated iPSCs (X-GFP–negative, X-GFP–medium, and X-GFP–high) compared to their control counterparts. As pluripotency acquisition is linked to X-chromosome reactivation during reprogramming, we analyzed the level of X-chromosome reactivation based on X-linked gene expression in each of the populations. For this, we calculated the allelic ratio [X mus/(X mus + X cas)] reflecting the proportion of reads from the X mus chromosome in NPCs, day 2, day 5, and day 7 cells undergoing reprogramming, and ESCs (Fig. 4G). When comparing the allelic ratio of IFNγ-treated cells to their control counterparts, we observed a significantly increased X mus proportion in IFNγ-treated iPSCs on day 7, when they undergo X-GFP reactivation. These results showed that not only X-GFP reactivation is more efficient (Fig. 2, C to E) but also endogenous chromosome-wide X-linked gene reactivation is more advanced upon early activation of the IFNγ pathway. Then, we analyzed the expression of genes from the X-inactivation center, a complex locus containing several coding and noncoding genes that control the expression of *Xist*, the master regulator of X-chromosome inactivation (Fig. 4H and fig. S6C) (*67*). We observed that *Xist* expression from the X mus chromosome was consistently lower in the IFNγ-treated cells in the X-GFP–negative, X-GFP–medium, and X-GFP–high populations at day 7, in comparison to the control (Fig. 4H), while the expression of *Xist* regulators at the X-inactivation center did not show clear changes (fig. S6C). Therefore, it is likely that the accelerated expression of naive pluripotency genes such as *Prdm14* and *Nanog* (Fig. 3B and figs. S5D and S6, D to G), which are known to repress *Xist* (*13*, *15*), contribute to the more efficient and advanced X-chromosome reactivation induced by IFNγ, rather than the *Xist* regulators at the X-inactivation center. *Xist* down-regulation in the IFNγ-treated day 7 X-GFP^−^ iPSCs (Fig. 4H) was not sufficient to induce a higher X mus proportion in this cell population (Fig. 4G). This could be due to either the not yet complete *Xist* down-regulation or the presence of additional mechanisms that maintain the X chromosome in an inactive state, such as DNA methylation, histone methylation, or deacetylation, in day 7 X-GFP^−^ cells even after IFNγ-treatment (*23*, *25*, *62*, *68*). We confirmed a faster loss of the Xist cloud in the IFNγ-treated X-GFP^−^ cells at day 7 of reprogramming compared to the control by RNA-FISH (fluorescence in situ hybridization) (17% and 49.8% of cells maintained the Xist cloud in the IFNγ condition and control, respectively) (fig. S8A). However, a large proportion of these cells still maintained the H3K27me3 spot (46.4% of X-GFP^−^ IFNγ-treated cells and 60.7% of X-GFP^−^ control cells) (fig. S8B) and equal levels of 5mC on the X chromosome were detected when comparing IFNγ-treated to control X-GFP^−^ cells in any of the genomic regions analyzed (promoters, gene bodies, and distal regions) (fig. S8, C and D). This indicates that, despite *Xist* down-regulation (Fig. 4H), X-chromosomal gene silencing in day 7 IFNγ-treated X-GFP^−^ cells might be maintained by H3K27me3 and DNA methylation.

In summary, our data indicate that IFNγ treatment during reprogramming results in a higher activation of JAK-STAT3 signaling during early reprogramming, an increased expression of naive pluripotency genes, and accelerated X-chromosome reactivation.

### IFNγ treatment promotes TET-mediated DNA demethylation in cells undergoing reprogramming

Global DNA demethylation is a hallmark of reprogramming to pluripotency in particular in female cells (*69*, *70*), and demethylation of X-chromosomal gene promoters is a critical step required for X-reactivation, although the demethylation mechanism of the X chromosome during reprogramming remains elusive (*23*). To gain further insight, we took advantage of mouse methylation BeadChip arrays (*71*) to study genome-wide and X-chromosomal 5-methylcytosine (5mC) and 5-hydroxy-methylcytosine (5hmC) levels, as 5mC is converted into 5hmC during active DNA demethylation by TET enzymes (*72*). To assess the impact of IFNγ treatment (day 0 to 5) on DNA demethylation during reprogramming, we analyzed the levels of 5mC and 5hmC in day 5 SSEA1^+^ and day 7 SSEA1^+^ X-GFP^+^ iPSCs, which is before and during the occurrence of X-reactivation, respectively (Fig. 4, G and H).

**Fig. 5.**
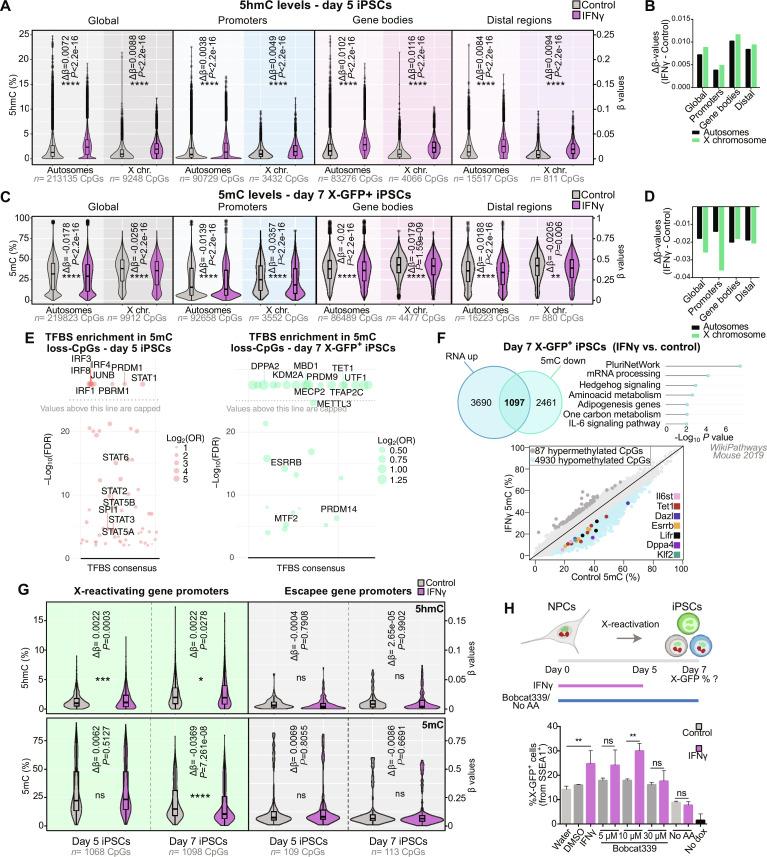
IFNγ promotes TET-mediated DNA demethylation during reprogramming. (**A** and **C**) Analysis of 5hmC in day 5 (A) or 5mC in day 7 X-GFP^+^ cells (C) (β values) of CpGs in autosomes and X chromosome for control and IFNγ (day 0 to 5) conditions, globally, in promoters (≤1 kb from transcription start site), gene bodies, and distal regions. Δβ-values (mean β-value IFNγ − mean β-value control) and *P* values (IFNγ versus control) are shown in the graphs. Statistics (unpaired *t* tests): **P < 0.01; ****P < 0.0001. (**B** and **D**) Δβ-values for 5hmC in day 5 [(B), corresponding to analysis in (A)] and 5mC in day 7 X-GFP^+^ [(D), corresponding to analysis in (C)] iPSCs. (**E**) TFBS enrichment analysis on IFNγ-5mC–hypomethylated CpGs [DMPs, logFC < (−0.1), *P* < 0.01] in day 5 (*n* = 360 CpGs) and day 7 X-GFP^+^ (*n* = 10,023 CpGs) iPSCs. −Log_10_(false discovery rate) capped values are above 25. (**F**) Venn diagram for up-regulated genes in the RNA-seq on IFNγ X-GFP^+^ day 7 iPSCs (*n* = 4787 genes) and genes associated with IFNγ-5mC–hypomethylated promoter CpGs [logFC < (−0.1), *P* < 0.01] (*n* = 3558 genes, 4930 CpGs), and pathway enrichment of common genes. Scatterplot representing 5mC levels from promoter CpGs in day 7 IFNγ versus control iPSCs [hypermethylated (dark gray) and hypomethylated (light blue) CpGs are shown, highlighting pluripotency genes with hypomethylated CpGs]. (**G**) Analysis of 5mC and 5hmC in X-reactivating (X-allelic ratio ≤ 0.135 in NPCs, *n* = 216 genes, 1068 to 1098 CpGs) and escapee (X-allelic ratio > 0.135 in NPCs, *n* = 20 genes, 109 to 113 CpGs) gene promoters in day 5 and 7 X-GFP^+^ iPSCs. Statistics (unpaired *t* tests): *P < 0.05; ***P < 0.001; ****P < 0.0001. (**H**) Treatment with IFNγ (day 0 to 5) was combined with addition of Bobcat339 or absence of ascorbic acid (AA) (day 0 to 7) during reprogramming. X-GFP expression was measured by flow cytometry at day 7 [*n* = 5 (control and IFNγ) and *n* = 3 (rest of conditions) technical replicates]. Statistics (unpaired t tests): **P < 0.01. Error bars represent SD.

We found that, in day 5 iPSC populations, IFNγ induced a general gain of the 5hmC mark on both autosomes and the X chromosome, globally and in all specific genomic regions analyzed (promoters, gene bodies, and distal regions) (Fig. 5, A and B), consistent with TET-mediated DNA demethylation promoted by IFNγ. However, this did not result in pronounced global differences in 5mC levels between control and IFNγ-treated iPSCs on day 5 (fig. S9, A and B). By contrast, in day 7 iPSCs, we observed a mild but significant 5hmC increase specifically on X chromosomes but not in autosomes (globally, in promoters, gene bodies, and distal regions) (fig. S9, C and D). Furthermore, we detected a global decrease of 5mC on day 7 IFNγ-treated iPSCs in all genomic regions analyzed (Fig. 5, C and D). The decrease in 5mC levels was stronger in X-chromosomal than in autosomal promoters. Together, these data suggest that IFNγ treatment early during reprogramming (day 0 to 5) results in enhanced DNA demethylation, indicated by increased 5hmC levels on day 5, and a subsequent more efficient loss of 5mC at day 7 in X-reactivating iPSCs, with the 5mC loss being predominant in X-chromosomal promoters.

Next, we analyzed transcription factor binding site (TFBS) enrichment (based on combined chromatin immunoprecipitation sequencing (ChIP-Seq) data from the Cistrome/ENCODE databases) in CpGs, which showed a loss of 5mC on both days 5 and 7 of reprogramming upon early IFNγ treatment (Fig. 5E). On day 5, we observed an enrichment of binding sites corresponding to STAT1 (and other proteins from the STAT family such as STAT2 and STAT3) and IRF transcription factors, in line with the ongoing IFNγ treatment. In day 7 X-GFP^+^ iPSCs treated with IFNγ, we found an enrichment of binding sites corresponding mostly to transcription factors related to pluripotency, such as DPPA2, TFAP2C, UTF1, ESRRB, and PRDM14, and epigenetic regulators (PRDM9, KDM2A, MBD1, TET1, MECP2, METTL3, and MTF2). TET1, likely recruited indirectly by pluripotency factors (*73*, *74*), appeared to be highly enriched in hypomethylated CpGs. Of note, TET1 oxidizes 5mC, leading to demethylation of DNA and showed increased expression upon IFNγ treatment in our RNA-sequencing (RNA-seq) dataset (Fig. 3C). In addition, we explored the overlap of the promoter CpGs that lost 5mC in IFNγ-treated day 7 X-GFP^+^ iPSCs with the genes gaining expression upon IFNγ treatment in the day 7 X-GFP^+^ populations (Fig. 5F). In total, up to 1097 common genes were found to lose 5mC in their promoter region and gain expression in this comparison. These genes were enriched in pathways such as the pluripotency network (*Tet1*, *Il6st*, *Dazl*, *Klf2*, *Esrrb*, *Lifr*, and *Dppa4*), mRNA processing, metabolism, and IL-6 signaling.

We then focused on the DNA methylation differences occurring on the X chromosome by analyzing the levels of 5hmC and 5mC in promoters of X-linked genes undergoing reactivation and in escapee genes, which are always active on the silenced X chromosome in differentiated cells (Fig. 5G). While we detected no changes for 5hmC or 5mC levels in escapee gene promoters, X-reactivating gene promoters showed a slight increase in 5hmC abundance in day 5 IFNγ-treated iPSCs, in line with the decreased levels of 5mC in day 7 X-GFP^+^ IFNγ-treated iPSCs. This increase in 5hmC and decrease in 5mC in X-reactivating gene promoters occurred specifically at X-linked genes that get reactivated later during reprogramming (“main”) (fig. S9F) (*24*). Of note, 468 of 470 differentially methylated X-chromosomal CpGs for 5mC showed a reduction in this mark (fig. S9G). Moreover, we performed allele-specific targeted amplicon sequencing (ASTA-Seq) of selected X-chromosomal loci in day 5 control and IFNγ-treated cells to quantify 5hmC and 5mC levels. For this, we selected single CpGs in promoters of X-reactivating genes (*Mtm1*, *Dlg3*, *Eda*, and *Zfp185*) found to be differentially (hydroxy)methylated in our DNA methylation arrays, and in promoters of two escapee genes (*Ddx3x* and *Eif2s3x*) as controls (fig. S10). As expected, escapee gene promoter CpGs displayed very low levels of 5mC and 5hmC (*75*). IFNγ treatment resulted in a gain of 5hmC in six of nine X-reactivating CpGs, either only on the inactive (four of six) or in both inactive and active X chromosomes (two of six). Moreover, IFNγ induced a reduction of 5mC levels on both X chromosomes in seven of nine X-reactivating CpGs. In line with the global DNA demethylation observed in our arrays (Fig. 5 and fig. S9), these results suggest that IFNγ is upstream of epigenetic reprogramming events that include X-chromosome reactivation.

TET enzymes play a key role in DNA demethylation (*72*) and are important for rewiring gene expression during pluripotency acquisition (*63*, *73*, *76*, *77*). As IFNγ treatment induces lower DNA methylation levels globally, and more pronouncedly on X-chromosomal promoters at day 7 of reprogramming, we wondered whether DNA demethylation catalyzed by TET enzymes was responsible for the higher efficiency in X-chromosome reactivation upon IFNγ treatment. To functionally test this hypothesis, we induced reprogramming with or without ascorbic acid/vitamin C (cofactor enhancing TET enzyme activity and thereby iPSC reprogramming) (*78*–*80*) and with or without Bobcat339 (a TET inhibitor) (*81*), and we analyzed the levels of X-GFP reactivation by flow cytometry on day 7 of reprogramming (Fig. 5H). In the presence of ascorbic acid, IFNγ induced a higher percentage of X-GFP in comparison to the no IFNγ control condition (*P* = 0.0026), consistent with our previous experiments (Fig. 2, C to E). Without addition of IFNγ, the X-GFP percentage did not change upon Bobcat339 treatment, suggesting that in control conditions, TET enzymes might be dispensable for X-GFP reactivation. While the addition of IFNγ together with low concentrations of Bobcat339 still induced a trend or significant increase in X-GFP percentage (5 μM: *P* = 0.16, 10 μM: *P* = 0.0026), this increase was no longer observed in the combination of IFNγ with a higher concentration of Bobcat339 (30 μM) (*P* = 0.92), nor in the absence of ascorbic acid (*P* = 0.27). These dose-dependent results are in line with the median inhibitory concentration (IC_50_) of Bobcat339 (33 and 73 μM for TET1 and TET2, respectively) (*81*). This shows that, upon TET inhibition by Bobcat339 or by the absence of the TET-cofactor ascorbic acid, IFNγ treatment loses its ability to enhance X-chromosome reactivation. As our gene expression analysis showed elevated *Tet1* levels upon IFNγ treatment on days 5 and 7 of reprogramming (Fig. 3, B, C, and G, and fig. S5, F and I), we generated *Tet1*^−/−^ ESCs and induced reprogramming after NPC differentiation (fig. S11, A to D). We observed that IFNγ treatment resulted in a 2.3 ± 0.77–fold increase in X-GFP percentage in *Tet1*^−/−^ cells, similarly as in the parental cells and scrambled gRNA controls (fig. S11D). This could be due to compensation by TET2, which is also expressed at this time of reprogramming (fig. S11E), since such a compensatory activity has previously been shown during iPSC reprogramming (*77*). Overall, this suggests that the enhancing effect of IFNγ on X-reactivation might be linked to the catalytic activity of TET enzymes (but not TET1 alone), indicating a potential mechanism of action.

## DISCUSSION

Here, we performed a genome-wide CRISPR KO screen to identify genes and pathways involved in pluripotency and X-chromosome reactivation, which revealed both activators and repressors of these processes. We uncovered a role of the IFNγ pathway, the early activation of which during NPC into iPSC reprogramming results in a reduced colony number, while accelerating pluripotency acquisition and enhancing X-chromosome reactivation later on.

The decreased colony number induced by early IFNγ treatment could be caused by a reduced expression of focal adhesion genes and increased apoptosis during the first 2 days of reprogramming. In line with this, IFNγ treatment has been previously reported to disrupt β1 integrin–mediated focal adhesions in intestinal epithelial cells (*82*). Moreover, ADAM (a disintegrin and metalloproteinase) proteins have been found to act as reprogramming barriers by antagonizing focal adhesion through inhibition of specific integrin dimers (*43*), indicating an important role of focal adhesion during reprogramming. On the other hand, the accelerated pluripotency acquisition upon early IFNγ treatment during iPSC induction could be related to the observed increased STAT3 activation. IFNγ has been reported to induce activation of the STAT3 protein (and not only its canonical target STAT1) (*83*). STAT3, which is activated by the LIF signaling pathway, plays a key role in self-renewal of pluripotent stem cells (*84*) and induces the expression of pluripotency genes by binding to their regulatory elements together with OCT4, SOX2, and NANOG (*85*). Therefore, the enhanced activation of STAT3 induced by IFNγ could result in the higher expression of the pluripotency network earlier as observed from day 5 onward, resulting in an acceleration of reprogramming. In line with this, a previous study demonstrated that constitutive activation of STAT3 induced a more efficient reprogramming, and inhibition of STAT3 signaling resulted in the absence of pluripotent colonies (*60*). In our study, we also demonstrated that STAT3 overexpression resulted in an increased X-GFP reactivation, which was not further enhanced when adding IFNγ, suggesting that IFNγ boosts X-reactivation through STAT3 signaling. Considering that IFNγ induces apoptosis and results in a lower colony number, a selection effect on cells undergoing reprogramming cannot be ruled out as a contributor to the enhanced pluripotency and X-reactivation observed upon treatment. However, in the context of STAT3 overexpression, IFNγ induced a lower colony number but did not enhance X-GFP reactivation, making a selection effect unlikely to be solely responsible for the observed phenotype.

Another mediator of IFNγ pathway activation to accelerated reprogramming and/or X-reactivation could be IRF1. Overexpression of this transcription factor in porcine embryonic fibroblasts has been found to increase the efficiency of reprogramming to iPSCs through higher activation of the LIF-STAT3 pathway (*86*). In our study, IFNγ-induced *Irf1* expression peaked on day 2 of reprogramming, which could contribute to an increased expression of the pluripotency network, directly or through an enhanced STAT3 activation. Furthermore, the IFNγ-mediated enhancement of X-reactivation efficiency was disrupted in *Irf1* KO cells. This shows that the increased and accelerated X-reactivation upon IFNγ pathway activation is, at least partially, dependent on IRF1.

The up-regulation of pluripotency network genes upon IFNγ treatment could also indirectly contribute to the observed enhanced X-chromosome reactivation. Pluripotency factors (e.g., OCT4, SOX2, NANOG, and PRDM14) act as *Xist* repressors directly by binding to its intron 1 (*13*, *15*, *87*, *88*) and indirectly by repressing the *Xist* activator *Rnf12/Rlim* (*14*, *15*) and by activating the *Xist* repressor *Tsix* (*87*, *89*). In our study, we observed a decreased *Xist* expression in IFNγ-treated cells on day 7 of reprogramming, which likely primed the cells for the enhanced X-reactivation. Even in day 7 X-GFP^−^ cells, *Xist* expression and X-chromosome coating were reduced after IFNγ treatment, but this was not sufficient to cause X-linked gene reactivation. This suggests the involvement of additional epigenetic silencing layers such as histone H3K27 methylation or DNA methylation of X-chromosomal promoters to be present, which need to be removed for X-reactivation to take place (*23*, *25*, *62*, *68*, *90*, *91*). We observed that day 7 IFNγ-treated X-GFP^−^ cells displayed similar X-chromosomal levels of DNA methylation than the control cells and still showed an H3K27me3 spot on the inactive X chromosome in a high proportion of cells, which could explain their maintained X-linked gene silencing.

DNA demethylation is a key step both for X-reactivation and for cellular reprogramming into iPSCs (*23*, *63*, *76*, *77*, *92*). Our DNA (hydroxy)methylation analyses revealed that IFNγ treatment induced increased levels of 5hmC on day 5 of reprogramming and decreased levels of 5mC at day 7 in cells undergoing X-reactivation. These results suggest that early treatment with IFNγ during reprogramming induces DNA demethylation, which was preferentially happening at promoters corresponding to and/or bound by pluripotency factors, reflecting an acceleration in reprogramming upon IFNγ treatment. The loss of 5mC levels was more pronounced in X-chromosomal than in autosomal promoters specifically at X-linked genes undergoing reactivation, while this effect was not observed in escapee gene promoters, which are always active including on the silent X chromosome. This is probably due to initially higher DNA methylation levels on the inactive X chromosome.

We found that IFNγ treatment induces the up-regulation of *Tet1* and *Gadd45a*, which are known to play important roles in DNA demethylation (*93*–*95*). The expression of these genes increased from day 5 of reprogramming onward, together with the up-regulation of the pluripotency network. Ten-eleven translocation (TET) enzymes (TET1, TET2, and TET3) oxidize 5mC into 5-hydroxymethylcytosine (5hmC) during DNA demethylation (*95*), and previous studies have demonstrated their importance in different reprogramming contexts. *Tet1* can replace *Oct4* in the OSKM reprogramming cocktail by demethylating and reactivating endogenous *Oct4* (*63*). Moreover, ablation of *Tet2* impaired iPSC generation from B cells (*77*) and MEFs (*76*), and TET1 and TET2 were shown to physically interact with NANOG and enhance neural stem cell into iPSC reprogramming (*73*). Here, we showed that in the presence of a TET inhibitor or in the absence of ascorbic acid [a TET cofactor that enhances TET activity (*79*, *96*) and is normally added to the medium in our reprogramming protocol], the IFNγ-driven effect on X-reactivation disappeared, suggesting that IFNγ-mediated epigenetic reprogramming on the X chromosome is related to TET activity. Although IFNγ treatment induced an up-regulation of *Tet1* expression from day 5 of reprogramming, even in the absence of TET1, addition of IFNγ still resulted in enhanced X-GFP reactivation levels, indicating that this mechanism is not only mediated by TET1. Alternatively, recruitment of TET enzymes by pluripotency factors such as NANOG and PRDM14 (*73*, *74*), which are up-regulated after IFNγ treatment, could mediate the enhanced DNA demethylation in our system. Our binding site analysis showed enrichment for pluripotency factors and TET1 in IFNγ-dependent hypomethylated CpGs. Of note, in line with a previous study in which *Tet1*, *Tet2*, and global 5hmC were reported to be dispensable for X-reactivation during reprogramming (*23*), we found that the use of the TET inhibitor did not result in a lower efficiency of X-reactivation in the absence of IFNγ treatment. This indicates that TET-mediated DNA demethylation is not needed for X-reactivation in a control reprogramming condition, but that enhanced demethylation after IFNγ treatment is TET dependent, boosting the efficiency and kinetics of the X-reactivation process. Furthermore, the absence of the TET-cofactor ascorbic acid during reprogramming decreased the efficiency of X-reactivation. This could be explained by the fact that ascorbic acid not only is a cofactor of TET enzymes but also induces H3K9me2 and H3K36me2/3 demethylation by enhancing the activity of histone demethylases (*97*, *98*). As these histone marks are erased during iPSC reprogramming (*97*, *99*, *100*), this might be the reason why the absence of ascorbic acid during reprogramming, but not the addition of the TET inhibitor, had a detrimental effect in X-reactivation efficiency in the absence of IFNγ treatment.

**Fig. 6.**
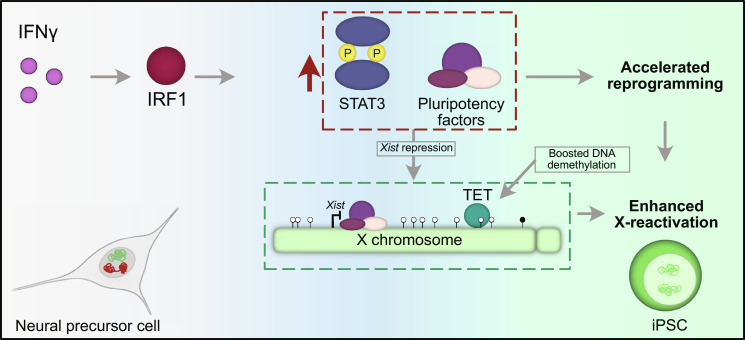
Model: Activation of the IFNγ pathway affects pluripotency acquisition and X-chromosome reactivation. The exposure to IFNγ in the early stages of NPC reprogramming into iPSCs induces the activation of IRF1 and a subsequent up-regulation and activation of STAT3 and the expression of pluripotency genes. This would lead to an accelerated reprogramming kinetics. Moreover, the higher expression of pluripotency factors would lead to *Xist* repression, and TET-mediated oxidation of methylated CpGs would enhance DNA demethylation globally and at X-chromosomal promoters of cells undergoing X-reactivation. This, together with the accelerated reprogramming, would explain the enhanced X-reactivation efficiency upon early IFNγ treatment during NPC reprogramming into iPSCs.

Overall, our study revealed the IFNγ pathway as a previously undescribed player in iPSC reprogramming and X-chromosome reactivation, and that early activation of the pathway results in accelerated reprogramming and enhanced X-reactivation in the NPC reprogramming system (Fig. 6). These findings provide mechanistic insight into the process of X-reactivation and have potential impact on the reprogramming field, with the possibility to improve the generation of iPSCs. A recent study demonstrated that IFNγ promotes stemness in cancer cells (*101*), supporting the idea that the IFNγ pathway might also be important for cellular dedifferentiation in other contexts, highlighting the broader relevance of our findings. Although our study has been performed in the mouse model system, the X-chromosome status has been shown to be a sensitive measure of stem cell quality and differentiation potential of human female pluripotent cells (*102*–*105*). Therefore, a comprehensive understanding of the mechanisms regulating the X-chromosome state in both mouse and human and its link to pluripotency will be needed to improve the generation of stem cell lines suitable for disease modeling and clinical applications.

### Limitations of the study

X-chromosome reactivation is tightly linked to pluripotency. The expression of a robust pluripotency network is correlated with *Xist* repression (*13*, *106*). One important limitation of our CRISPR screen is that we did not identify genes or pathways playing a role exclusively in X-chromosome reactivation and not affecting pluripotency. Although the pluripotency reporter (P-RFP) from the PaX system (*24*) allows us to distinguish cells that only acquire late pluripotency from cells that also undergo X-reactivation, these populations were not included in the CRISPR screen due to the limited cell number showing these features and the high number of cells needed for the screen to maintain a faithful gRNA representation. Therefore, we could identify pathways playing a role in both late pluripotency acquisition and X-reactivation, but we were not able to find genes or pathways that would uncouple these two processes. However, the fact that very few cells reached late pluripotency without undergoing X-reactivation indicates how closely related these two processes are. Finally, IFNγ did not enhance X-reactivation efficiency in the broadly used MEF reprogramming system. However, IFNγ treatment during NPC differentiation delayed X-chromosome inactivation (opposite to its role in NPC reprogramming) and was shown to influence cell fate changes in other studies (*101*, *107*–*109*), indicating its relevance in several cellular contexts.

## MATERIALS AND METHODS

### Cell lines used

#### PaX cell line

As our starting cell line, we used the PaX (pluripotency and X-chromosome reporter) reprogramming system (*24*). The PaX system consists of a hybrid *M. musculus*/*M. castaneus* ESC line (*110*), in which the X-chromosome activity can be traced by the expression of an X-GFP reporter introduced into the *Hprt* locus of the *Musculus* X chromosome (X mus), which undergoes preferential inactivation when differentiated due to a truncation of the *Tsix* gene (*111*, *112*). This cell line also contains a Tet-On inducible *MKOS* (*cMyc*-*Klf4*-*Oct4*-*Sox2*) reprogramming cassette and a reverse tetracycline–controlled transactivator (rtTA) inserted into the *Sp3* locus that allow iPSC induction from differentiated cells upon treatment with doxycycline (*49*). Moreover, it contains a pluripotency reporter (*Nanog* promoter-RFP or P-RFP), allowing the identification of cells that achieve a late pluripotent state during reprogramming.

#### PaX-iCas9 cell line

For the generation of the PaX-*iCas9* cell line, 5 million PaX ESCs were nucleofected with 3 μg of the Piggybac TRE-Cas9 plasmid, which was a gift from M. Calabrese (Addgene, plasmid #126029) (*47*), and 3 μg of a transposase plasmid provided by M. Saitou (*113*). The Amaxa Mouse Embryonic Stem Cell Nucleofector Kit was used (Lonza, VPH-1001), program A-24. Two days after transfection, cells were selected with Hygromycin B Gold (200 μg/ml) (Ibian Technologies, ant-hg-1) for 13 days, changing medium every day. Cells were single cell–sorted by FACS using a BD FACSAria II and replated on 0.2% gelatin–coated 96-well plates in serum-LIF medium with Hygromycin B Gold (200 μg/ml). Colonies were expanded for 9 days and genotyped to detect the presence of the *Cas9* sequence. Genomic DNA (gDNA) was isolated from *iCas9*-transfected ESC clones [incubation at 55°C overnight with lysis buffer: 10% of 1 M tris-HCl (pH 8), 5 mM EDTA, 0.1% SDS, 0.2 M NaCl in milliQ water]. DNA was precipitated with isopropanol 1:1 and washed with EtOH 70%. The lysates were diluted 1:10 in water. For PCR amplification, a DreamTaq PCR Master Mix was used (Thermo Fisher Scientific, K1082). To functionally test the KO production efficiency of selected clones, a gRNA targeting *GFP* was cloned into a Lenti-guide puro plasmid [a gift from F. Zhang (Addgene plasmid #52963; http://n2t.net/addgene:52963; RRID:Addgene_52963)] (*114*). 293T cells were transfected with the plasmids pCMVR8.74 [a gift from D. Trono (Addgene plasmid #22036; http://n2t.net/addgene:22036; RRID:Addgene_22036)] and pCMV-VSV-G [a gift from B. Weinberg (Addgene plasmid #8454; http://n2t.net/addgene:8454; RRID:Addgene_8454)] (*115*) and the Lenti-guide puro-*GFP* gRNA plasmid. Viral harvesting and concentration was performed 48 hours after transfection using the Lenti X Concentrator (Clontech, 631231), following the manufacturer’s instructions. The PaX-*iCas9* ESC clones were infected with lentiviruses containing the *GFP* gRNA, and the virus was removed after 24 hours. Forty-eight hours after infection, ESC medium containing puromycin (2 μg/ml) (Ibian Technologies, ant-pr-1) was added to the cells. Cells were exposed to puromycin for 4 days, before treatment with doxycycline for 6 days and measuring the percentage of X-GFP^+^ cells by flow cytometry every day, using a BD LSRFortessa Cell Analyzer.

#### Stat1^−/−^, Irf1^−/−^, Tet1^−/−^, and Stat3^−/−^ cell lines

gRNA pairs targeting the *Stat1*, *Irf1*, *Tet1*, or *Stat3* genes, or a scrambled gRNA, were cloned into a Lenti-guide puro [a gift from F. Zhang (Addgene plasmid #52963; http://n2t.net/addgene:52963; RRID:Addgene_52963)] or Lenti-guide blast [a gift from B. Stringer (Addgene plasmid #104993; http://n2t.net/addgene:104993; RRID:Addgene_104993)] plasmids (*114*, *116*). 293T cells were thawed and maintained in DMEM (Thermo Fisher Scientific, 31966021) for 5 days. The day before transfection, 20 million 293T cells were seeded on one 150-mm plate per gRNA. The next day, 293T cells were transfected with 7.5 μg of the plasmid pCMVR8.74 [a gift from D. Trono (Addgene plasmid #22036; http://n2t.net/addgene:22036; RRID:Addgene_22036)], 3 μg of the plasmid pCMV-VSV-G [a gift from B. Weinberg (Addgene plasmid #8454; http://n2t.net/addgene:8454; RRID:Addgene_8454)] (*115*), and 10 μg of the Lenti-guide puro/blast-gRNA plasmid using PEI transfection reagent (1 mg/ml) (Tocris, 7854). Incubation with the transfection mix was done for 5 hours at 37°C, and the medium was replaced for 25 ml of viral harvest medium per 150-mm plate [DMEM with 30% fetal bovine serum (FBS) and penicillin/streptomycin (100 U/ml)]. Viral harvesting was performed 48 hours after transfection, followed by filtering with 0.45-μm polyethersulfone (PES) filters. Viruses were concentrated by using the Lenti X Concentrator (Clontech, 631231), following the manufacturer’s instructions. The PaX-*iCas9* ESC line was infected in suspension with lentiviruses containing the gRNA pairs and Polybrene (8 μg/ml) (Merck, TR-1003-G). Plates were centrifuged at 2250 rpm for 30 min after 6 hours of infection (when cells were already attached to the well surface), and virus was removed after 24 hours. Forty-eight hours after infection, ESC medium containing puromycin (2 μg/ml) (Ibian Technologies, ant-pr-1) and/or blasticidin (5 μg/ml) (Ibian Technologies, ant-bl-1) was added to the cells. Cells were exposed to puromycin for 4 days and to blasticidin for 6 days. For *Stat1*, *Irf1*, and *Tet1* KOs, cells were treated with doxycycline for 7 days, followed by single-cell sorting with a BD FACSAria II SORP cytometer, PCR screening of clones to detect the presence of gRNA pairs and Western blot to detect the absence of IRF1 or STAT1 protein, or PCR to detect the deletion and Sanger sequencing for *Tet1*^−/−^ clones. For *Stat3* KO pools, PCR screening of ESCs untreated with doxycycline allowed the selection of clones containing the gRNAs, which were further treated with doxycycline for 8 days for KO induction and finally validated by Western blot to detect the reduction of the STAT3 protein.

#### Stat3-BFP overexpression ESC pools

Gibson cloning (*117*) was used to generate a Lenti-i*Stat3-BFP* containing the TRE (doxycycline inducible) promoter followed by the *Stat3* mouse cDNA, a T2A, and a BFP sequence (available from Addgene, plasmid #216870, https://www.addgene.org/216870/). For this, DNA fragments were amplified by PCR: *Stat3* sequence from cDNA from mouse E14 cells, the backbone containing a TRE promoter and a WPRE sequence from the pLV-TRE plasmid (A.B. laboratory), and the T2A-BFP sequence from the pKLV-U6gRNA(BbsI)-PGKpuro2ABFP plasmid [a gift from K. Yusa (Addgene plasmid #50946; http://n2t.net/addgene:50946; RRID:Addgene_50946)] (*118*). PCR was performed using a PrimeStar HS DNA Polymerase (Takara, R010A), adapting the annealing temperatures and extension time following the manufacturer’s instructions. DNA fragments were assembled using a Gibson Assembly Master Mix (NEB, E2611). Virus was produced as described in the section above. The PaX-*iCas9* ESC line was infected with the i*Stat3-BFP* lentivirus in suspension with Polybrene (8 μg/ml) (Merck, TR-1003-G), followed by centrifugation of the plates at 2250 rpm for 30 min after 6 hours of infection (when cells were already attached to the well surface), and virus was removed after 24 hours. Cells were expanded and treated with doxycycline for 48 hours before sorting the BFP-medium and BFP-high cell pools with a BD FACSAria II SORP cytometer. These cells were expanded and passaged for a week without doxycycline in the medium to recover endogenous STAT3 levels and used for NPC differentiation and reprogramming.

### Primary cell derivation and animal husbandry

MEFs were obtained from male E12.5 and female E14.5 mouse embryos for feeder derivation and for MEF reprogramming, respectively [details in “Feeders (irradiated MEFs)” and “Reprogramming of MEFs into iPSCs” sections]. Mouse care and procedures were conducted according to the protocols approved by the Ethics Committee on Animal Research of the Parc de Recerca Biomedica de Barcelona (PRBB) and by the Departament de Territori i Sostenibilitat of the Generalitat de Catalunya (reference no. 10469).

### Feeders (irradiated MEFs)

MEFs from E12.5 male embryos were expanded for 10 days at 37°C with 5% CO_2_ and 5% O_2_ in DMEM (Thermo Fisher Scientific, 31966021) supplemented with 10% FBS (Thermo Fisher Scientific, 10270106), 25 mM Hepes (Thermo Fisher Scientific, 15630056), 1 mM sodium pyruvate (Thermo Fisher Scientific, 11360070), 1× MEM nonessential amino acids (NEAAs) (Thermo Fisher Scientific, 11140050), penicillin/streptomycin (50 U/ml) (Ibian Technologies, P06-07100), and 0.1 mM 2-mercaptoethanol (Thermo Fisher Scientific, 31350010) before γ-irradiation (30 kGy) for inactivation.

### ESC culture

Mouse ESCs were cultured at 37°C with 5% CO_2_ on 0.2% gelatin-coated plates in serum/LIF medium: DMEM (Thermo Fisher Scientific, 31966021) supplemented with 10% FBS (Embryonic Stem (ES) pre-tested, Capricorn, FBS-ES-12A), LIF (1000 U/ml) (ORF Genetics, 01-A1140-0100), 25 mM Hepes (Thermo Fisher Scientific, 15630056), 1 mM sodium pyruvate (Thermo Fisher Scientific, 11360070), 1× MEM NEAAs (Thermo Fisher Scientific, 11140050), penicillin/streptomycin (50 U/ml) (Ibian Technologies, P06-07100), and 0.1 mM 2-mercaptoethanol (Thermo Fisher Scientific, 31350010). The medium was changed every day. Passaging of cells was done using 0.05% trypsin-EDTA (Thermo Fisher Scientific, 25300054). PCR mycoplasma tests were performed monthly.

### NPC differentiation

NPC differentiation and reprogramming were done similarly as in (*24*). Mouse ESCs were thawed on serum/LIF medium 5 days before induction and passaged for 3 consecutive days onto 0.2% gelatin–coated plates at 1.75 × 10^5^ cells per cm^2^. The day of induction, the medium was changed to 2i/LIF: 50% Neurobasal medium (Thermo Fisher Scientific, 12348017), 50% DMEM F12 (Thermo Fisher Scientific, 21041025), 1× N2 (Thermo Fisher Scientific, 17502048), 1× B27 (Thermo Fisher Scientific, 12587001), 3 μM CHIR99021 (Sigma-Aldrich, SML1046), 0.4 μM PD0325901 (Selleck Chemicals, S1036), and LIF (1000 U/ml) (ORF Genetics, 01-A1140-0100). After 6 hours, cells were dissociated with Accutase (Merck Millipore, SF006) and plated on 0.2% gelatin–coated T75 flasks at a density of 6.67 × 10^3^ cells/cm^2^ in RHBA medium (Takara Bio, Y40001). The medium was changed every 2 days. From day 6, the medium was supplemented with EGF (10 ng/ml) (R&D Systems, 236-EG-200) and basic fibroblast growth factor (bFGF) (10 ng/ml) (Thermo Fisher Scientific, 13256029). From day 8 onward, the medium was also supplemented with 10 μM ROCK inhibitor (Selleck Chemicals, S1049). On day 9 of differentiation, cells were dissociated with Accutase (Merck Millipore, SF006) and incubated with anti-SSEA1 microbeads (Miltenyi Biotec, 130-094-530) at 4°C for 15 min. Magnetic-activated cell sorting (MACS) separation was performed to enrich for SSEA1^−^ cells. Staining with SSEA1 eFluor 660 antibody 1:50 (Thermo Fisher Scientific, 50-8813-42) was performed at 4°C for 15 min. A BD FACSAria II SORP was used to sort the SSEA1^−^, P-RFP^−^, X-GFP^−^ cells. Sorted cells (1.5 × 10^6^) were plated on a 0.2% gelatin–coated well of a six-well plate in RHBA supplemented with EGF, bFGF, and ROCK inhibitor. The medium was changed every day until day 12.

### Reprogramming of NPCs into iPSCs

At day 12 of NPC differentiation, the NPC differentiation medium (RHBA with EGF, bFGF, and ROCK inhibitor) was supplemented with l-ascorbic acid (25 mg/ml) (Sigma-Aldrich, A7506) and doxycycline (1 mg/ml) (Tocris, 4090/50). One day later, cells were dissociated with Accutase (Merck Millipore, SF006) and seeded at different densities depending on day of analysis (49,100 cells per cm^2^ for day 5, 12,300 cells per cm^2^ for day 7, and 2850 cells per cm^2^ for day 10) on top of male irradiated MEFs (feeders) on 0.2% gelatin–coated plates in iPSC medium: DMEM (Thermo Fisher Scientific, 31966021), 15% FBS (ES pre-tested, Capricorn, FBS-ES-12A), 25 mM Hepes (Thermo Fisher Scientific, 15630056), 1 mM sodium pyruvate (Thermo Fisher Scientific, 11360070), 1× MEM NEAAs (Thermo Fisher Scientific, 11140050), penicillin/streptomycin (50 U/ml) (Ibian Technologies, P06-07100), and 0.1 mM 2-mercaptoethanol (Thermo Fisher Scientific, 31350010), supplemented with LIF (1000 U/ml), l-ascorbic acid (25 mg/ml), and doxycycline (1 mg/ml). The medium was changed on days 3, 5, 7, 8, and 9.

### Lentiviral CRISPR KO screen

#### gRNA library amplification

The gRNA library used for the screening was the Mouse Improved Genome-wide KO CRISPR Library v2 (a gift from K. Yusa, Addgene, #67988) (*48*), with 90,230 gRNAs targeting 18,424 genes (average of 5 gRNAs per gene). NEB 10-beta Electrocompetent *Escherichia coli* (NEB, C3020K) were electroporated in five concomitant reactions [each reaction containing 20 μl of bacteria and 1 μl of the gRNA library (20 ng/μl)]. After electroporation, 1 ml of SOC recovery medium was added to each reaction and bacteria were incubated at 37°C for 1-hour shaking. Bacteria were then grown overnight at 37°C shaking in 1 liter of 2xTY [NaCl (5 g/liter), tryptone (16 g/liter), yeast extract (10 g/liter)] + ampicillin (100 μg/ml). The plasmid gRNA library was purified by using the QIAfilter Plasmid Maxi Kit (Qiagen, 12263), following the manufacturer’s instructions. Concentration was measured with NanoDrop (Thermo Fisher Scientific, ND-1000).

#### Generation of lentiviral gRNA library

For the lentiviral library production, 293T cells were thawed in DMEM (Thermo Fisher Scientific, 31966021) supplemented with 10% FBS (Thermo Fisher Scientific, 10270106). After 2 and 4 days, cells were passaged into three and five T175 flasks, respectively (2.5 × 10^6^ cells and 40 ml of medium per flask). At day 7, cells were seeded on 10 T175 flasks for transfection at a density of 18 million cells and 25 ml of medium per T175 flask. After 24 hours, transfection was done by using 31 μg of the plasmid library, 38.8 μg of the plasmid pCMVR8.74 [a gift from D. Trono (Addgene plasmid #22036; http://n2t.net/addgene:22036; RRID:Addgene_22036)], 3.88 μg of the plasmid pCMV-VSV-G [a gift from B. Weinberg (Addgene plasmid #8454; http://n2t.net/addgene:8454; RRID:Addgene_8454)] (*115*), 6 ml of Opti-MEM (Thermo Fisher Scientific, 11058021), and 305 μl of TransIT-LT1 Transfection Reagent (Mirus Bio, MIR 2300) per T175 flask. Incubation with the transfection mix was done for 8 hours at 37°C, and the medium was replaced for 60 ml of viral harvest medium per T175 flask [DMEM with 30% FBS and penicillin/streptomycin (100 U/ml)]. Viral harvesting was performed 36 hours after transfection, followed by filtering with 0.45-μm PES filters. Viruses were concentrated by using the Lenti X Concentrator (Clontech, 631231), following the manufacturer’s instructions.

#### gRNA library lentiviral infection of ESCs

PaX-*iCas9* ESCs were thawed in serum/LIF medium and amplified for 3 days. Thirteen T175 flasks coated with 0.2% gelatin were seeded with 18.5 × 10^6^ ESCs per flask, in 27 ml of ESC medium with Polybrene (8 μg/ml) (Merck, TR-1003-G) and the lentiviral gRNA library. The next day, the medium was replaced with serum/LIF medium containing puromycin (2 μg/ml) (Ibian Technologies, ant-pr-1). The medium with antibiotics was replaced every other day for 1 week. In parallel, 72 hours after infection, the percentage of BFP^+^ cells was measured using a BD LSR Fortessa flow cytometer to calculate the multiplicity of infection (0.06) and the coverage (200 cells per gRNA). gRNA sequencing was performed to check gRNA representation.

#### NPC differentiation, reprogramming, and cell isolation by FACS

For the CRISPR screening, two independent biological replicates (each one with two technical replicates) were performed in different differentiation and reprogramming inductions. To this end, 1.2 × 10^8^ pooled lentiviral-infected ESCs were thawed on three 150-mm plates in serum/LIF medium 5 days before induction and passaged 3 days in a row onto 0.2% gelatin–coated plates at a density of 25 million cells per 150-mm plate (four plates). The day of induction, the medium was changed to 2i/LIF for 6 hours, and cells were then dissociated with Accutase (Merck Millipore, SF006) and seeded on 52 gelatin-coated T75 flasks at a density of 7.5 × 10^5^ cells per flask in RHBA medium. Differentiation was followed as previously described. Sorting of SSEA1^−^ P-RFP^−^ X-GFP^−^ NPCs was performed on day 9, as described above. NPCs (4 × 10^7^) were sorted in Replicate 1, and 8.4 × 10^7^ NPCs were sorted in Replicate 2. Each 1.5 × 10^6^ sorted cells were plated on a 0.2% gelatin–coated well of a six-well plate in RHBA supplemented with EGF, bFGF, and ROCK inhibitor. The medium was changed every day until day 12. Cell pellets of 2 × 10^6^ cells were collected for gRNA abundance analysis.

For reprogramming, mouse male feeders were thawed on gelatin-coated 150-mm plates (~10 million cells per plate, 46 plates for Replicate 1 and 60 plates per Replicate 2) 1 day before reprogramming induction in DMEM (Thermo Fisher Scientific, 31966021) supplemented with 10% FBS (Thermo Fisher Scientific, 10270106), 25 mM Hepes (Thermo Fisher Scientific, 15630056), 1 mM sodium pyruvate (Thermo Fisher Scientific, 11360070), 1× MEM NEAAs (Thermo Fisher Scientific, 11140050), penicillin/streptomycin (50 U/ml) (Ibian Technologies, P06-07100), and 0.1 mM 2-mercaptoethanol (Thermo Fisher Scientific, 31350010). At day 12 of NPC differentiation, the NPC differentiation medium (RHBA with EGF, bFGF, and ROCK inhibitor) was supplemented with l-ascorbic acid (25 mg/ml) and doxycycline (1 mg/ml). One day later, cells were dissociated with Accutase (Merck Millipore, SF006) and seeded on 46 (Replicate 1) and 60 (Replicate 2) 150-mm plates on top of feeders (3000 cells per cm^2^) in iPSC medium supplemented with LIF (1000 U/ml), l-ascorbic acid (25 mg/ml), and doxycycline (1 mg/ml). The medium was changed on days 3, 5, 7, 8, and 9. At day 10 of reprogramming, cells were dissociated with 0.25% trypsin-EDTA (Thermo Fisher Scientific, 25200056). Trypsinization was stopped with DMEM–10% FBS containing deoxyribonuclease (DNase) I (10 μg/ml) (Sigma-Aldrich, 11284932001). Cells were then stained with SSEA1 eFluor 660 antibody 1:100 (Thermo Fisher Scientific, 50-8813-42) at 4°C for 45 min in rotation. A BD FACSAria II SORP was used to sort three different populations, according to the BFP fluorescence (gRNA plasmid), SSEA1-eFluor 660 fluorescence (pluripotency marker), and X-GFP (X-chromosome status): nonpluripotent population (BFP^+^ SSEA1^−^ X-GFP^−^), early pluripotent population (BFP^+^ SSEA1^+^ X-GFP^−^), and late pluripotent, X-chromosome reactivated population (BFP^+^ SSEA1^+^ X-GFP^+^). Cell pellets were collected and frozen at −80°C until processed for gDNA extraction.

#### Sample preparation and gRNA sequencing

gDNA was extracted from cell pellets using the DNeasy Blood and Tissue Kit (Qiagen, 69504). NPCs (1.15 × 10^7^) and cells (1.4 × 10^7^) of each reprogramming population were processed for Replicate 1, and NPCs (2.64 × 10^7^) and cells (1.8 × 10^7^) of each reprogramming population were processed for Replicate 2. For amplification of the gRNAs and introduction of the Illumina-sequencing adapters, two consecutive PCRs were performed by using the Q5 High-Fidelity DNA Polymerase (NEB, M0491). For PCR1, all the extracted gDNA was used for amplification, in PCRs of 50 μl with 1 μg of gDNA as template. For this PCR1, all forward primers and all reverse primers were mixed together in the “Forward primer mix” and “Reverse primer mix” in equal amounts to have a final concentration of 10 μM (1.67 μM of each primer). Sequences can be found in table S5 and follow the International Union of Pure and Applied Chemistry (IUPAC) nucleotide code. After electrophoresis in a 2% agarose gel, the DNA was purified by using a QIAEX II Gel Extraction Kit (Qiagen, 20051). For PCR2, 16 reactions of 50 μl were performed per sample, by using 5 ng of the purified PCR1 product as template for each reaction. Independent PCRs for each sample were done with reverse primers containing different barcodes for sample identification. Electrophoresis in a 2% agarose gel was performed before DNA purification from gel. PCR components and quantities are indicated in Table 1. PCR conditions are specified in Table 2. Sequencing was performed using an Illumina HiSeq 2500 [50–base pair (bp) single-end].

**Table 1.** PCR components for gRNA library amplification.

Component	Volume for 50-μl reaction
*PCR1*	
5× Q5 buffer	10 μl
Deoxynucleotide triphosphate (dNTP) 10 mM	1 μl
F mix 10 μM (Stag0_F to Stag5_F, 1.67 μM each)	2.5 μl
R mix 10 μM (Stag0_R to Stag5_R, 1.67 μM each)	2.5 μl
H_2_O	To 50 μl
DMSO	3 μl
Q5 polymerase	0.5 μl
DNA	1 μg
*PCR2*	
5× Q5 buffer	10 μl
Deoxynucleotide triphosphate (dNTP) 10 mM	1 μl
F primer 10 μM (TS-HT-D5x-1-F)	2.5 μl
R primer 10 μM (different for each sample)	2.5 μl
H_2_O	To 50 μl
Q5 polymerase	0.5 μl
DNA (PCR1 product)	5 ng

**Table 2.** PCR conditions for gRNA library amplification.

Temperature	Time	Cycles
*PCR1*		
98°C	3 min	–
98°C	30 s	× 20
56.5°C	20 s	
72°C	60 s	
72°C	2 min	–
4°C	Hold	–
*PCR2*		
98°C	3 min	–
98°C	30 s	× 8
56.5°C	20 s	
72°C	60 s	
72°C	2 min	–
4°C	Hold	–

### CRISPR screening analysis

The gRNA sequencing from the CRISPR KO screening was analyzed with MAGeCK software (*119*). The gRNA abundance of each population was determined by taking into account the two biological replicates (with two technical replicates each). The gRNA abundance comparisons were performed pairwise. The list of overrepresented genes for the comparison of the reprogramming populations (nonpluripotent, early pluripotent, and late pluripotent) to NPCs was obtained by selecting the top 250 genes of each comparison ranked by positive score in the MAGeCK software and filtering for unique genes from the obtained list. The list of essential genes (underrepresented for the comparison of each reprogramming population to NPCs) was obtained by filtering common genes with an Robust Rank Aggregation (RRA) score of <0.05 and log_2_ fold change (log_2_FC) of <(−0.75). For the pairwise comparisons among the reprogramming populations (early pluripotent versus nonpluripotent, late pluripotent versus early pluripotent), the selection of hits was performed by using an RRA score of <0.05, a log_2_FC of <(−0.8)/>0.8, and a “goodsgrna” equal or higher than 3. Gene Ontology (GO) pathway enrichment analysis was performed with the obtained filtered genes using the library “WikiPathways Mouse 2019” in the Enrichr website (https://maayanlab.cloud/Enrichr/).

### CRISPR screening pathway validation—molecule screening

For the molecular screening, NPC differentiation was performed as previously described. At day 13 of NPC differentiation, cells were dissociated with Accutase (Merck Millipore, SF006) and seeded on top of male irradiated MEFs in iPSC medium supplemented with LIF (1000 U/ml), l-ascorbic acid (25 mg/ml), and doxycycline (1 mg/ml). Three seeding densities were used: 49,100 cells per cm^2^ for analysis at day 5 of reprogramming, 12,300 cells per cm^2^ for analysis at day 7, and 2850 cells per cm^2^ for analysis at day 10. The medium was changed on days 3, 5, 7, and 9. Molecules were added from day 0 to 5, from day 5 to 10, and from day 0 to 10 for all the conditions. At days 5, 7, and 10, cells were dissociated with 0.25% trypsin-EDTA (Thermo Fisher Scientific, 25200056) and stained with SSEA1 eFluor 660 antibody 1:100 (Thermo Fisher Scientific, 50-8813-42) at 4°C for 30 min in rotation. A BD LSRFortessa Cell Analyzer was used to check the SSEA1-eFluor 660 fluorescence (early pluripotency marker) and X-GFP (X-chromosome status).

### Reprogramming of MEFs into iPSCs

MEFs were derived from female mouse E14.5 embryos containing a doxycycline-inducible reprogramming cassette (*Oct4*–*Sox2*–*Klf4*–*c-Myc*) in the *Col1a1* locus and the reverse tetracycline–controlled transactivator (M2rtTA) in the *Rosa26* locus (*120*). These cells also contained a GFP reporter on one X chromosome (*121*) and a mutation in the *Hprt* locus on the other X chromosome (*122*). Female MEFs with an inactive X-GFP chromosome were isolated as previously described (*27*) and cultured at 37°C with 5% CO_2_ and 5% O_2_ in DMEM (Thermo Fisher Scientific, 31966021) supplemented with 10% FBS (Thermo Fisher Scientific, 10270106), 25 mM Hepes (Thermo Fisher Scientific, 15630056), 1 mM sodium pyruvate (Thermo Fisher Scientific, 11360070), 1× MEM NEAAs (Thermo Fisher Scientific, 11140050), penicillin/streptomycin (50 U/ml) (Ibian Technologies, P06-07100), and 0.1 mM 2-mercaptoethanol (Thermo Fisher Scientific, 31350010) for up to three passages.

One day before reprogramming induction, male irradiated feeders were thawed on 0.2% gelatin-coated plates (100,000 cells/cm^2^). The day of reprogramming induction, female reprogrammable MEFs were dissociated with 0.05% trypsin-EDTA (Thermo Fisher Scientific, 25300054) and seeded on feeders [7150 cells/cm^2^ or 28,570 cells/cm^2^ (4×)] in iPSC medium (previously described), supplemented with LIF (1000 U/ml), l-ascorbic acid (25 mg/ml), and doxycycline (1 mg/ml). The medium was changed on days 4, 6, 8, and 10 (doxycycline withdrawal). Recombinant Mouse IFNγ Protein (R&D Systems, 485-MI-100) was added to the iPSC medium at a concentration of 10 ng/ml from day 0 to 6 or day 2 to 6.

### Flow cytometry

A BD FACSAria II SORP was used for cell sorting. NPCs were sorted at around 3500 events per second, maximum flow rate of 4 with a 100-μm nozzle to increase cell viability after sorting. SSEA1^−^ P-RFP^−^ X-GFP^−^ cells were selected. iPSCs were sorted at around 8000 events per second, using the 85-μm nozzle, selecting the cell populations regarding SSEA1-eFluor 660 and X-GFP fluorescence. A BD LSRFortessa Cell Analyzer or a BD LSR II Flow Cytometer were used for flow cytometry analysis experiments. For CRISPR screening experiments, FVS780 (BD Horizon, 565388) was used as a viability dye at 1.1 ng/ml. For the rest of the experiments, DAPI (4′,6-diamidino-2-phenylindole) (Biotium, BT-40043) was used at 0.1 μg/ml. Flow cytometry analyses were done by using FlowJo v10.7.1 software (BD Life Sciences).

### IFNγ treatment during NPC to iPSC reprogramming

Recombinant Mouse IFNγ Protein (R&D Systems, 485-MI-100) was added to the iPSC medium at a concentration of 10 ng/ml from day 0 to 5, day 5 to 10, or day 0 to 10. Further analysis was performed by flow cytometry using a BD LSRFortessa Cell Analyzer, AP staining, immunofluorescence or cell sorting by a BD FACSAria II SORP for Western blotting, RNA-seq, or DNA methylation arrays.

### IFNγ treatment during NPC differentiation

Recombinant Mouse IFNγ Protein (R&D Systems, 485-MI-100) was added to the NPC medium at a concentration of 10 ng/ml from day 0 to 5, day 5 to 10 or day 0 to 10. At days 5 and 10, cells were dissociated with Accutase and stained with SSEA1 eFluor 660 antibody 1:100 (Thermo Fisher Scientific, 50-8813-42) at 4°C for 30 min on ice. A BD LSRFortessa Cell Analyzer was used to check the SSEA1-eFluor 660 fluorescence (early pluripotency marker) and X-GFP (X-chromosome status).

### RNA isolation, cDNA synthesis, and qRT-PCR

Total RNA isolation was performed with the RNeasy Plus Mini Kit (Qiagen, 74136) or RNeasy Micro Kit (Qiagen, 74004). Concentration was quantified with NanoDrop (Thermo Fisher Scientific, ND-1000). cDNA was synthesized using a High-Capacity RNA-to-cDNA Kit (Thermo Fisher Scientific, 4387406). qRT-PCR was performed in triplicates for each sample, using Power SYBR Green PCR Master Mix (Thermo Fisher Scientific, 4367659). Gene expression levels were calculated as 2^−∆CT^ normalized with the average cycle threshold (CT) of the housekeeping gene *Gapdh*.

### DNA methylation modifier experiments

Reprogramming was done as described previously, combining the IFNγ (10 ng/ml, day 0 to 5) (R&D Systems, 485-MI-100) with the addition of the TET inhibitor molecule Bobcat339 (concentration of 5, 10, or 30 μM, R&D Systems, 6977/10) or in the absence of ascorbic acid (normally added to the reprogramming medium at 25 mg/ml, Sigma-Aldrich, A7506) from day 0 to 7. NPCs (4.3 × 10^4^) were plated per well of a 12-well plate. On day 7 of reprogramming, cells were detached from the plates using 0.25% trypsin-EDTA (Thermo Fisher Scientific, 25300054) and stained with SSEA1-eFluor 660 antibody (1:100) and DAPI. Analysis was done using a BD LSRFortessa Cell Analyzer.

### Apoptosis assay

Day 13 NPCs (treated with doxycycline and ascorbic acid during 24 hours) were induced for reprogramming on carboxyfluorescein diacetate succinimidyl ester (CFSE)–stained feeders (to sort these cells out; 0.5 μM CFSE CellTrace, Thermo Fisher Scientific, C34554), stained, and plated the day before on gelatin-coated plates (1 × 10^6^ feeders seeded per well of a six-well plate). NPCs (2 × 10^5^) were seeded per well of a six-well plate, in iPSC medium in the absence or presence of IFNγ (R&D Systems, 485-MI-100) at 10 ng/ml. Three experimental replicates were done. After 48 hours, cells were detached from the plates using 0.05% trypsin-EDTA (Thermo Fisher Scientific, 25300054) and stained with annexin V–APC antibody and DAPI (0.1 μg/ml, Biotium, BT-40043) using the Annexin V Apoptosis Detection Kit (Thermo Fisher Scientific, 88-8007-72). Analysis was done using a BD LSR II Flow Cytometer.

### Western blotting

For protein extraction, cells were resuspended in Laemmli buffer and boiled at 95°C for 10 min. Protein extracts were loaded in a 10% acrylamide gel (Bio-Rad, 1610149), and electrophoresis was performed for protein separation. Transference was done into a polyvinylidene difluoride (PVDF) membrane (Sigma-Aldrich, P2938). Blocking of the membrane was performed using 4% milk in tris-buffered saline (TBS)–0.5% Tween 20 (Sigma-Aldrich, P7949) for 1 hour at room temperature. The membrane was incubated overnight at 4°C with the corresponding antibodies [rabbit anti-STAT1 1:1000 (Cell Signaling Technology, 14994S), rabbit anti–phospho-STAT1 Tyr^701^ 1:1000 (Cell Signaling Technology, 7649S), mouse anti-PP1α 1:1000 (Santa Cruz Biotechnology, sc-7482), rabbit anti-STAT3 1:1000 (Cell Signaling Technology, 12640S), rabbit anti-phospho–STAT3 Tyr^705^ 1:1000 (Cell Signaling Technology, 9145S), rabbit anti-IRF1 1:1000 (Cell Signaling Technology, 8478S), and mouse anti–α-tubulin 1:10,000 (Sigma-Aldrich, T6074)] in blocking solution. Secondary antibody incubation was performed in polyclonal rabbit anti-mouse–horseradish peroxidase (HRP) antibody 1:2000 (Dako, P0260) or polyclonal goat anti-rabbit–HRP antibody 1:2000 (Dako, P0448) in blocking solution for 1.5 hours at room temperature. For washes, TBS–0.5% Tween 20 was used. The membranes were developed by using an EZ-ECL kit (Reactiva, 120500120) and x-ray films (Rosex Medical, EWPJH).

Fiji (*123*) was used to calculate the relative intensities of STAT3 and pSTAT3 Tyr^705^. The “mean gray value” of each region of interest (ROI) with the same area was calculated for both loading controls and proteins of interest, followed by the inversion of the pixel density and the calculation of the ratio for each sample.

### AP staining

Cells were washed with phosphate-buffered saline (PBS) and fixed with 4% paraformaldehyde (PFA) for 10 min at room temperature. Washing with milliQ water was done before adding the AP staining solution [10 ml of milliQ water, 10 mg of Fast red TR salt hemi(zinc chloride) salt (Santa Cruz Biotechnology, sc-215025), and 400 μl of Naphthol AS-MX phosphate (Sigma-Aldrich, 855-20ML)] for 10 min. Cells were washed again with water, which was aspirated before scanning.

### Immunofluorescence

Cells were seeded on 0.2% gelatin-coated coverslips on 12-well plates, where the reprogramming experiments were performed. On the specific reprogramming day, coverslips were washed with PBS and fixed at room temperature for 10 min with 4% PFA (Electron Microscopy Sciences, 15713S). After fixation, samples were washed with PBS and permeabilized with PBS–0.5% Triton (Sigma-Aldrich, T8787) for 10 min at room temperature. Then, coverslips were washed once with 70% EtOH and kept in 70% EtOH at −20°C until staining was performed. For immunostaining, coverslips were washed in PBS and blocking was performed with PBS–2% bovine serum albumin (BSA) (Sigma-Aldrich, SLCK2178)–0.2% Triton for 1.5 hours at room temperature. Primary antibody incubation was done overnight at 4°C [rabbit anti-pSTAT1 Tyr^701^ (Cell Signaling Technology, 7649S), rabbit anti-pSTAT3 Tyr^705^ (Cell Signaling Technology, 9145S), mouse anti-SSEA1 (Sigma-Aldrich, MAB4301), rabbit anti-NANOG (Novus Bio, nb100-588), chicken anti-GFP (Abcam, ab13970), and mouse anti-H3K27me3 (Active Motif, 61017)]. Coverslips were washed three times with PBS for 5 min at room temperature, and secondary antibody incubation was performed for 2 hours at room temperature [goat anti-mouse A647 (Abcam, 150115), goat anti-rabbit A555 (Thermo Fisher Scientific, A21429), and goat anti-chicken A488 (Thermo Fisher Scientific, A11039)]. Coverslips were washed three times with PBS for 5 min at room temperature, adding DAPI (10 μg/ml) (Biotium, BT-40043) in the last wash. Mounting was done with Vectashield Antifade Mounting Medium (Vector Laboratories, H-1000-10). Images were taken with a Leica SP5 confocal/MP inverted microscope.

### RNA FISH

Cells were washed and resuspended in PBS. Two hundred microliters of cell suspension (120,000 cells) was loaded onto a cytospin funnel. Cytospin was done for 10 min at 1000 rpm. Slides were air-dried for 1 min and incubated in a coplin jar in ice-cold PBS + 2 mM RVC (Ribonucleoside Vanadyl Complex, NEB, S1402S) for 5 min, ice-cold CSK buffer (100 mM NaCl, 200 mM sucrose, 10 mM Pipes, 3 mM MgCl_2_ in milliQ water, pH 6.8) + 2 mM RVC for 30 s, ice-cold CSK buffer + 0.5% Triton + 2 mM RVC for 1 min, ice-cold CSK buffer + 2 mM RVC for 1 min, room temperature 4% PFA for 10 min, and ice-cold 70% EtOH for 2 min. Slides were stored in 70% EtOH at −20°C overnight before RNA FISH. Probe mix [10 ng/μl of Xist-Cy5 (Sx9) probe prepared by Nick Translation (Roche, 10976776001), 20 mM RVC, mouse Cot-1 DNA (0.1 μg/μl) (Thermo Fisher Scientific, 18440016), and yeast tRNA (0.5 μg/μl) (Life Technologies, 15401029) in hybridization buffer (10% dextran sulfate and 25% formamide in 2× Saline Sodium Citrate or SSC)] was incubated for 10 min at 80°C and for 30 min at 37°C. In parallel, slides were dehydrated in EtOH at room temperature [70%, 80%, 90%, and 100% EtOH (2 min each)] and air-dried completely. Fifteen microliters of pre-annealed probe was pipetted onto the cells and incubated overnight at 37°C in a humidified chamber. Slides were then washed [3 × 5-min washes in 50% (v/v) formamide in 2× SSC at 45°C and 3 × 5-min washes in 2× SSC at 37°C] and mounted using Vectashield with DAPI (Vector Laboratories, H-1200-10). Images were taken with a Zeiss Cell Observer fluorescence microscope.

### X-chromosome paint

Cells were washed twice with PBS for 2 min, fixed with 4% PFA for 10 min, washed twice for 2 min with PBS, incubated with PBS–0.5% Triton for 10 min, PBS 0.1% Tween for 2 min, and 0.1 N HCl for 5 min, washed twice with 2× SSCT (Saline Sodium Citrate, 0.1% Tween) for 1 min, and incubated with 2× SSCT–50% formamide for 10 min (all steps at room temperature). Hybridization mix [20% volume of XMP X Green mouse chromosome paint (MetaSystems Probes, D-1420-050-FI) in hybridization buffer (10% dextran sulfate and 25% formamide in 2× SSC)] was added to the cells, denatured at 80°C for 3 min, and incubated overnight at 37°C in a humidified chamber. Samples were washed [3 × 5 min in 2× SSCT 50% formamide at 45°C, 3 × 5 min in 2× SSCT at 45°C, 1 × 5 min in 2× SSCT at room temperature, and 1 × 5 min in 2× SSCT + DAPI (10 μg/ml) (Biotium, BT-40043) at room temperature] and mounted with Vectashield Antifade Mounting Medium (Vector Laboratories, H-1000-10). Images were taken with a Leica SP5 confocal/MP inverted microscope.

### RNA-seq experiments

For the RNA-seq of day 2 doxycycline-treated cells, male irradiated MEFs were stained with 0.5 μM CFSE CellTrace (Thermo Fisher Scientific, C34554) and seeded on 0.2% gelatin-coated plates upon thawing. One day after, day 13 NPCs were seeded on top of the CFSE-stained feeders in iPSC medium to induce reprogramming, in the presence or absence of IFNγ (R&D Systems, 485-MI-100, 10 ng/ml). On day 2 of reprogramming, CFSE-negative cells were sorted by using a BD FACSAria II SORP, and cell pellets were kept at −80°C until RNA extraction.

For the RNA-seq of iPSCs on days 5 and 7, day 13 NPCs were seeded on top of feeders in iPSC medium to induce reprogramming, in the presence or absence of IFNγ (R&D Systems, 485-MI-100, 10 ng/ml) for the first 5 days. On days 5 and 7, cells were dissociated with 0.25% trypsin-EDTA (Thermo Fisher Scientific, 25200056). Trypsinization was stopped with DMEM–10% FBS containing DNase I (10 μg/ml) (Sigma-Aldrich, 11284932001). Cells were then incubated with anti-SSEA1 microbeads (Miltenyi Biotec, 130-094-530) at 4°C for 15 min. MACS separation was performed to enrich for SSEA1^+^ cells. Staining with SSEA1 eFluor 660 antibody 1:100 (Thermo Fisher Scientific, 50-8813-42) was performed at 4°C for 45 min. Sorting was performed by using a BD FACSAria II SORP. For iPSCs at day 5, SSEA1^+^ cells were sorted. For iPSCs at day 7, SSEA1^+^ cells were separated into three populations: X-GFP–negative, X-GFP–medium, and X-GFP–high cells. Cell pellets were kept at −80°C until RNA extraction.

RNA was extracted from cell pellets by using an RNeasy Plus Mini Kit (Qiagen, 74136) or RNeasy Micro Kit (Qiagen, 74004). Concentration was quantified with NanoDrop (Thermo Fisher Scientific, ND-1000).

### RNA-seq analysis

The RNA library preparation was performed by ribosomal RNA depletion using the TruSeq Stranded Total RNA Library Preparation Kit (Illumina, 20020596). Sequencing was performed by an Illumina HiSeq 2500 (50 bp paired-end or 125 bp paired-end, merged and trimmed to 50 bp for further analysis). RNA-seq analysis was done similarly as in (*11*). FastQ files passing the quality control were aligned to the mm10 reference genome, which contained CAST/EiJ and 129S1/SvImJ SNP positions masked. The SNP positions of the mouse strains were obtained from https://www.sanger.ac.uk/data/mouse-genomes-project/. A VCF file containing only the SNP positions from CAST/EiJ and 129S1/SvImJ strains was generated. Alignment of reads to the reference genome was done using STAR (*124*) with implementation of the WASP method (*125*) to filter allele-specific alignments. The output BAM files were used to obtain the read counts using the HTseq tool (v0.6.1) (*126*). These steps were performed using a published Nextflow pipeline (*127*) and following the workflow described in https://github.com/biocorecrg/allele_specific_RNAseq. Around 75 to 85% of reads aligned to the reference genome, corresponding to 3.5 × 10^7^ to 5 × 10^7^ mapped reads. Differential expression analysis was done with the R package DESeq2 (v1.32.0) (*128*). Differentially expressed genes were identified performing pairwise comparisons. Read counts were normalized by library size and filtered for having a mean across the samples >10. Log_2_FC shrinking was done with the “normal” parameter. Up-regulated and down-regulated genes were selected by filtering for a positive or negative log_2_FC (respectively) and an adjusted *P* value of <0.1 (for control versus IFNγ comparisons in all time points). GO pathway enrichment analysis was performed with the obtained filtered genes using the library “WikiPathways Mouse 2019” in the Enrichr website (https://maayanlab.cloud/Enrichr/). To run the PCA, we used the top 500 genes showing highest variability. ggplot2 R package (v3.3.5, https://ggplot2.tidyverse.org) was used for generating the heatmap (representing the *z* score of Fragments Per Kilobase Million (FPKM) of selected genes in the different cell populations). To calculate the pluripotency score, the expression levels of *Nanog*, *Zfp42*, *Dppa4*, *Dppa5a*, *Esrrb*, *Prdm14*, and *Sall4* in each time point were normalized to the expression of these genes in the ESCs, and the average of these values was represented for two independent replicates. For the allelic ratio analysis, 315 protein-coding genes that showed over 25% of total X-linked gene expression in the X cas in all the populations were selected. To calculate the X mus proportion, we divided the X mus expression to the sum of X mus and X cas expression [X mus/(X mus + X cas)] in the selected genes.

### DNA (hydroxy)methylation experiments and analyses

Reprogramming was induced in the presence or absence of IFNγ (10 ng/ml) (from day 0 to 5) (R&D Systems, 485-MI-100). SSEA1^+^ day 5 iPSCs (four replicates from different reprogramming rounds) and SSEA1^+^ X-GFP^−/+^ day 7 iPSCs (two replicates from different reprogramming rounds) were sorted with a BD FACSAria II SORP. DNA was extracted using Wizard Genomic DNA Purification Kit (Promega, A1120). After measuring DNA quantity by Qubit (Thermo Fisher Scientific), 2 μg of each sample was evenly split for the oxidation reaction [oxidative bisulfite (OxBS)–treated samples] and the mock-oxidation reaction [bisulfite (BS)–treated samples], where the oxidant solution was replaced by water following the TrueMethyl oxBS Module manufacturer’s instructions (NuGEN-Tecan, 0414). Both aliquots were then processed in parallel for all stages of the protocol. After the oxidation reaction where 5-hydroxymethylcytosine is oxidized to 5-formylcytosine (5fC) and 5mC stays unchanged, the BS treatment converts 5fC and all nonmethylated cytosines to uracil, while 5mC is not altered.

For samples to be run on the Illumina Infinium Mouse Methylation BeadChip Array (Illumina, 20041558), 7 μl of recovered TrueMethyl template was mixed with 1 μl of 0.4 N NaOH following the manufacturer’s instructions. All subsequent steps were completed following the Infinium HD Assay Methylation protocol (https://emea.support.illumina.com/content/dam/illumina-support/documents/documentation/chemistry_documentation/infinium_assays/infinium_hd_methylation/infinium-hd-methylation-guide-15019519-01.pdf).

The DNA methylation status of the studied samples was obtainedusing the Infinium Mouse Methylation BeadChip Array (~285,000 methylation sites). GenomeStudio Software 2011 (Illumina) was used to process raw signal intensities. The mm10 mouse genome manifest from Illumina was used as reference, as described in the Illumina manifest file associated with the Infinium Mouse Methylation BeadChip. DNA methylation β values were obtained from raw IDAT files using the software’s default normalization with control probes and background subtraction. The 5mC signal was extracted from the β values of the OxBS samples, while the 5hmC signal was obtained by subtracting the β values of the BS samples from those of the OxBS samples. All further analyses were performed using the R environment (v4.2.3). To remove erratic probe signals, quality control steps were applied. Probes that did not pass the intensity threshold were removed (intensity <1000), as well as those with detection *P* value >0.01. 5mC and 5hmC levels were then batch-corrected using the Limma R package (v3.50.3) (*129*).

Differentially (hydroxy)methylated positions [(h)DMPs] were extracted using the function topTable from the limma package (v3.50.3), adjusting by Benjamini-Hochberg method. CpGs with *P* values <0.01 were selected, and further filtering with log fold change (logFC) was also performed (logFC ± 0.1). The package gplots (v3.1.3, https://cran.r-project.org/web/packages/gplots/index.html) was used to create the heatmap of the X-chromosomal DMPs. To assign the genomic features corresponding to each CpG, ChIPseeker package (v1.30.3) (*130*) together with org.Mm.eg.db (v3.14.0) for annotation was used. The distribution violin and box plots were generated with ggplot2. GO pathway enrichment analysis was performed with the obtained filtered genes using the library “WikiPathways Mouse 2019” in the Enrichr website (https://maayanlab.cloud/Enrichr/). The overlap analysis of DMPs and RNA-seq differentially expressed genes was done using Venny 2.1.0 (https://bioinfogp.cnb.csic.es/tools/venny/).

For selection of X-linked reactivating or escapee genes, protein-coding genes that showed over 25% of total X-linked gene expression in the X cas in all the populations analyzed from the RNA-seq dataset and showed an allelic ratio equal or under 0.135 in NPCs (X-reactivating genes) or above 0.135 in NPCs (escapee genes) were selected [similarly as in (*11*, *24*)]. The lists of “early” and “main” X-reactivating genes were obtained from (*24*).

TFBS enrichment was analyzed with the SeSAMe R package (v1.16.1) (*131*), using the “KYCG.MM285.TFBSconsensus.20220116” database based on ChIP-Seq data from all available cell types/tissues and factors at Cistrome/ENCODE.

### Allele-specific (hydroxy)methylation analysis by amplicon oxidative BS sequencing

An allele-specific targeted amplicon oxidative BS–sequencing (ASTA-Seq) protocol was conceived to assess the allele-specific (hydroxy)methylation status of regions surrounding several differentially (hydroxy)methylated CpGs [D(h)MP] in X-reactivating gene promoters identified at day 5 or 7 of reprogramming by DNA methylation arrays, including escapee gene controls, similarly as in (*132*, *133*). Genes were classified as X-reactivating based on a previous study (*24*) and validated according to our RNA-seq dataset. Regions to be analyzed were selected to include species-specific SNPs and at least one D(h)MP while maintaining an amplicon size of 200 to 500 bp. Paired OxBS and the mock-oxidation reaction (BS)–treated DNA from day 5 reprogramming samples were used as templates for a two-step PCR amplification protocol before library preparation for high-throughput sequencing. In the first PCR, 0.5 ng of DNA was used to amplify the ROIs. In the second PCR, 1 to 3 μl of the first-step products were amplified using staggered forward and reverse primers designed to contain partial P5 and P7 adaptor sequences, respectively, at the 5′ and two to five random nucleotides (N) before the gene-specific sequence. Staggered forward and reverse primers were pooled together equimolarly before amplification. The amplicons from the different ROIs were gel-purified, quantified, and pooled together for library preparation. Four libraries corresponding to control and IFNγ samples (BS/OxBS) at day 5 were prepared for sequencing, and subsequent steps were completed following the MiSeq System protocol (MiSeq Reagent Kit v3, Illumina). Next-generation sequencing was performed using MiSeq (300 bp, single-end). Primer design for PCR amplification was done with the Meth Primer web tool (*134*) (https://www.urogene.org/cgi-bin/methprimer/methprimer.cgi), avoiding any CpG residues within the primer sequence or at an SNP position (table S5). All the PCR amplifications were performed with IMMOLASE DNA Polymerase (BIO-21046) with a custom program (Table 3) adapted to each primer pair annealing temperature and resulting amplicon size.

**Table 3.** PCR conditions for allele-specific (hydroxy)methylation analysis.

Temperature	Time	Cycles
96°C	10 min	
96°C	30 s	× 48
58°C	30 s	
72°C	30 s	
72°C	10 min	

Analysis of 5mC and 5hmC percentages was done independently for each CpG contained in the PCR amplicons. Custom Perl scripts for the allele-specific (hydroxy)methylation analysis are available at https://github.com/eblancoga/ASTA-Seq and https://zenodo.org/records/10676879. Amplicons that contained the SNP and at least one CpG in the same read were analyzed, with coverages ranging from around 14,000 to 600,000 reads. First, reads were assigned to their corresponding genes by identification of the primer used for PCR2. Then, a 15- to 20-nucleotide sequence (BS-converted) upstream of the SNP was used to classify the reads in Mus or Cas. Next, a 15- to 20-nucleotide sequence (BS-converted) upstream of the CpG was used to determine the presence of CG or TG. Percentages of CG were calculated independently for Mus and Cas in control or IFNγ-treated samples and in BS or OxBS samples. 5mC percentages were calculated by analyzing the percentage of CG in OxBS samples, while 5hmC percentages were calculated as %CG (BS) − %CG (OxBS). In BS samples, unmethylated Cs are converted to Ts, while methylated Cs (5mC and 5hmC) stay as Cs. In OxBS samples, 5hmC is first oxidized and then converted into Ts, together with unmethylated Cs, while 5mC stays as C. Because of this, in this analysis, we only took into account CpGs in which percentages of CG in BS samples were higher than percentages of CG in OxBS samples, resulting in a value corresponding to 5hmC percentage, which was positive for all samples and conditions. CpGs with a negative value for 5hmC <(−1)% in any of the conditions (Mus or Cas, control, or IFNγ), were discarded as they could not be biologically explained, while the ones in which the negative value was between 0% and −1% were considered as 0%, as this was an acceptable variability range. Finally, 5mC and 5hmC percentages for Mus and Cas CpGs were compared between control and IFNγ samples. For escapee gene controls, 5mC and 5hmC levels were included in the same graph due to similarity in methylation levels, while for the rest of X-reactivating genes, CpGs were represented independently due to high variability of methylation levels.

### Statistical analyses

For experiments with technical replicates, unpaired *t* tests were performed. For experiments with independent reprogramming rounds, paired *t* tests were done. A confidence interval of 95% was used. In the molecule validation experiments, paired *t* tests were performed. Each molecule was compared to their diluent control: BMP2, BMP4, TGFβ, and IFNγ were compared to water, while the rest of the molecules were compared to dimethyl sulfoxide (DMSO) controls. Each treatment was compared to its specific time point (day 0 to 5, day 5 to 10, day 0 to 10). For allelic ratio and DNA methylation data comparisons, unpaired *t* tests were performed. When means of fold changes are specified in the text, ±SD values are indicated. Statistical analyses were performed with R (v. 4.2.3) or GraphPad Prism (v. 6).

## Supplementary Materials

### This PDF file includes:

Figs. S1 to S11 Legends for tables S1 to S6 References
