# Supplementary material for: The interferon γ pathway enhances pluripotency and X-chromosome reactivation in iPSC reprogramming
Source: Sci Adv. 2024 Aug 7;10(32):eadj8862. doi: 10.1126/sciadv.adj8862 (PMC11305397; doi:10.1126/sciadv.adj8862)
Supplement: Supplementary file 1 — Figs. S1 to S11 Legends for tables S1 to S6 References [file sciadv.adj8862_sm.pdf]

Science Advances
AAAS

Supplementary Materials for

The interferon γ pathway enhances pluripotency and X-chromosome reactivation in iPSC reprogramming

Mercedes Barrero *et al.*

Corresponding author: Bernhard Payer, bernhard.payer@crg.eu

Sci. Adv. **10**, eadj8862 (2024)
DOI: 10.1126/sciadv.adj8862

The PDF file includes:

Figs. S1 to S11
Legends for tables S1 to S6
References

Other Supplementary Material for this manuscript includes the following:

Tables S1 to S6

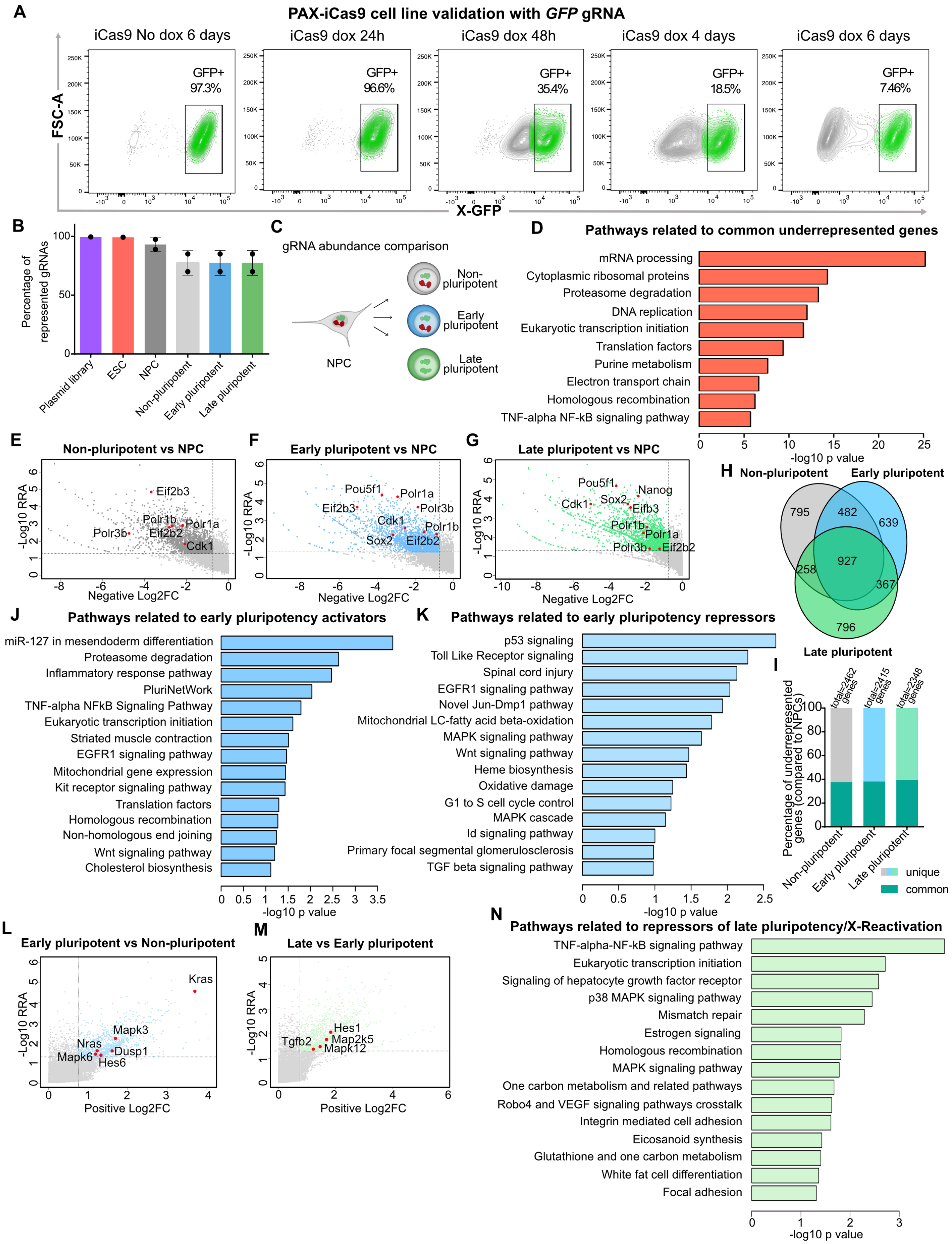

Fig. S1. *Legend on the next page.*

Fig. S1. CRISPR screen reveals molecular networks involved in reprogramming and X-chromosome reactivation. Related to **Fig. 1**. (**A**) Validation of knockout efficiency by flow cytometry. Flow cytometry analysis during 6 days of doxycycline treatment in the X-GFP *iCas9* ESC line was done to measure the X-GFP percentage decay in cells containing a gRNA targeting the *GFP* gene. Gating shows the X-GFP+ population. (**B**) Percentage of gRNA representation in the plasmid library, infected ESCs and the 4 populations analyzed in two independent screening rounds: NPCs and day 10 reprogramming populations (non-pluripotent, early pluripotent, late pluripotent). Error bars represent SD. (**C**) gRNA abundance comparisons (related to D-I): NPCs to non-pluripotent, early pluripotent and late pluripotent populations. (**D**) Pathways related to common underrepresented genes (n=927 genes) in the three reprogramming populations compared to NPCs (WikiPathways Mouse 2019). For all comparisons, an RRA score < 0.05 and Log2FC < -0.75 (underrepresented) filtering was applied. (**E-G**) Representation of genes with negative Log2FC (underrepresented) vs -log10 RRA in the non-pluripotent (E), early pluripotent (F) and late pluripotent (G) populations compared to NPCs (RRA cutoff = 0.05, Log2FC cutoff = -0.75). (**H**) Venn diagram (using Venny 2.1.0) representing overlap of underrepresented genes (compared to NPCs) in each of the sorted populations at day 10 of reprogramming. (**I**) Bar plot showing percentages of common and unique underrepresented genes (compared to NPCs) in each of the sorted populations at day 10 of reprogramming. (**J**) Pathways (WikiPathways Mouse 2019) related to underrepresented genes in the "early pluripotent vs non-pluripotent" comparison (activators of early pluripotency, n=1361 genes) (RRA score < 0.05 and Log2FC < -0.8 filtering was applied). (**K**) Pathways (WikiPathways Mouse 2019) related to overrepresented genes in the "early pluripotent vs non-pluripotent" comparison (repressors of early pluripotency, n=693 genes) (RRA score < 0.05 and Log2FC > 0.8 filtering was applied). (**L**) Representation of genes with positive Log2FC (overrepresented) vs -log10 RRA (RRA cutoff = 0.05, Log2FC cutoff = 0.75) in the "early pluripotent vs non-pluripotent" comparison (repressors of early pluripotency). (**M**) Representation of genes with positive Log2FC (overrepresented) vs -log10 RRA (RRA cutoff = 0.05, Log2FC cutoff = 0.75) in the "late pluripotent vs early pluripotent" comparison (repressors of late pluripotency, X-reactivation). (**N**) Pathways (WikiPathways Mouse 2019) related to overrepresented genes in the "late pluripotent vs early pluripotent" comparison (repressors of late pluripotency, X-reactivation, n=839 genes) (RRA score < 0.05 and Log2FC > 0.8 filtering was applied).

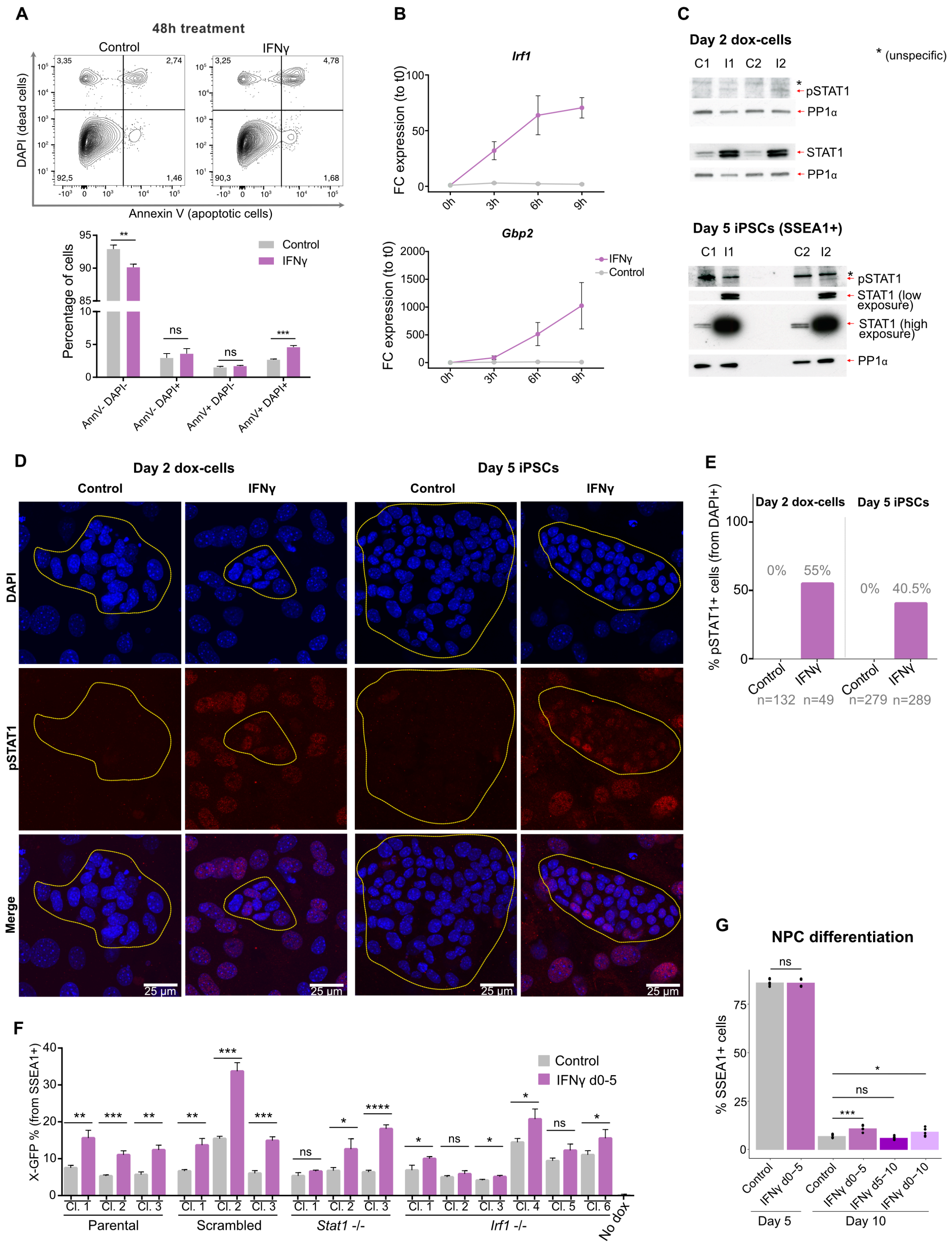

Fig. S2. *Legend on the next page.*

Fig. S2. Interferon γ pathway activation during iPSC reprogramming. Related to **Fig. 2**. (**A**) Analysis of apoptosis by annexin V and DAPI staining with flow cytometry after 48h of reprogramming induction +/- IFNγ treatment (n=3 technical replicates). Statistics (unpaired t-tests): ns = non significant; ** = $p<0.01$; *** = $p<0.001$. Error bars represent SD. (**B**) RT-qPCR on mRNA for *Irf1* and *Gbp2* expression at 0h, 3h, 6h and 9h from reprogramming induction +/- IFNγ treatment (relative to t0). Error bars represent SD (n=3 technical replicates). (**C**) Western blotting of STAT1 and pSTAT1 (Tyr701) on day 2 and day 5 reprogramming cells +/- IFNγ treatment (loading control: PP1α). (**D**) Immunofluorescence of pSTAT1 (Tyr701) on day 2 and day 5 reprogramming cells +/- IFNγ treatment. Scale bar = 25 μm. Outlines highlight colonies of cells undergoing reprogramming, characterized by smaller nuclei and tight aggregation. (**E**) Percentage of pSTAT1-positive cells from immunofluorescence in (D). Numbers of counted cells are indicated on the bottom of the graph. (**F**) (Related to Fig. 2F-H). Flow cytometry quantification of total X-GFP percentages (from SSEA1+ cells) on day 7 of reprogramming for 3 clones from the parental cell line, 3 clones containing a scrambled gRNA, 3 *Stat1 -/-* clones and 6 *Irf1 -/-* clones, including three technical replicates for each clone, in IFNγ-treated cells and untreated controls. Statistics (unpaired t-tests): ns = non significant; * = $p<0.05$; ** = $p<0.01$; *** = $p<0.001$; **** = $p<0.0001$. Error bars represent SD. (**G**) (Related to Fig. 2I-K). Quantification of SSEA1 percentage on days 5 and 10 of NPC differentiation by flow cytometry in control and IFNγ treatment conditions (n=6 independent replicates). Statistics (paired t-tests): ns = non significant, * = $p<0.05$; *** = $p<0.001$.

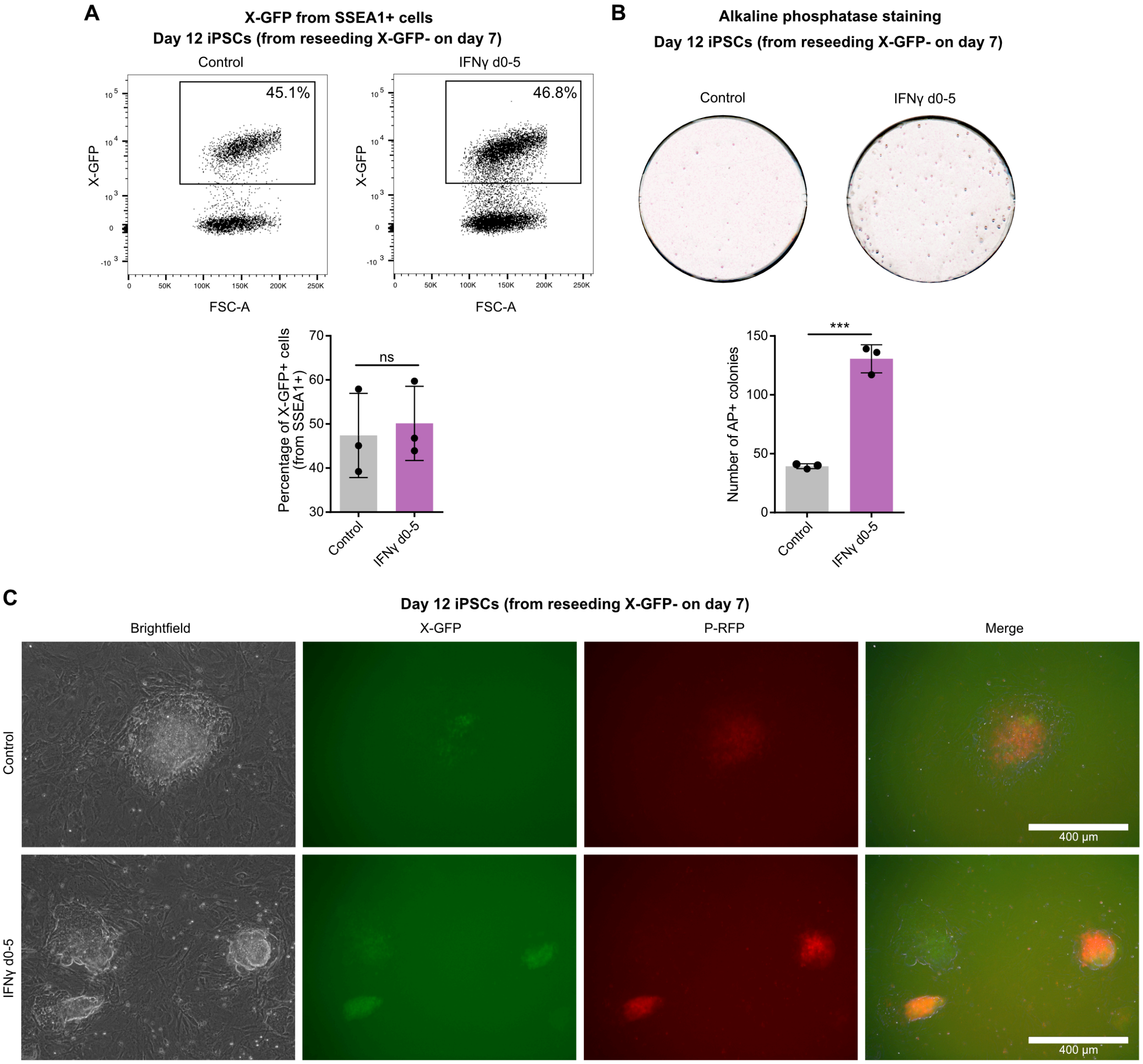

Fig. S3. Reseeding of IFNγ-treated day 7 X-GFP negative cells results in higher colony formation and equal X-GFP reactivation at day 12. Related to **Fig. 2.** (**A**) Flow cytometry plots of X-GFP expression (from SSEA1+ cells) in control and IFNγ treatment (day 0-5) on day 12 iPSCs after reseeding SSEA1+ X-GFP- cells on day 7 of reprogramming (gating shows the X-GFP+ population), and bar plot representation of X-GFP percentages (from SSEA1+ cells) (n=3 technical replicates). Statistics (unpaired t-tests): ns (non significant). Error bars represent SD. (**B**) Alkaline Phosphatase (AP) stainings on day 12 of reprogramming after reseeding SSEA1+ X-GFP- cells on day 7 of reprogramming, in control and IFNγ treatment (d0-5) and counting of AP+ colonies (n=3 technical replicates). Statistics (unpaired t-tests): *** = $p<0.001$. Error bars represent SD. (**C**) Brightfield and fluorescent images (X-GFP and P-RFP) of live cells at day 12 of reprogramming after reseeding SSEA1+ X-GFP- cells on day 7, in control and IFNγ treatment (d0-5). Scale bar = 400 µm.

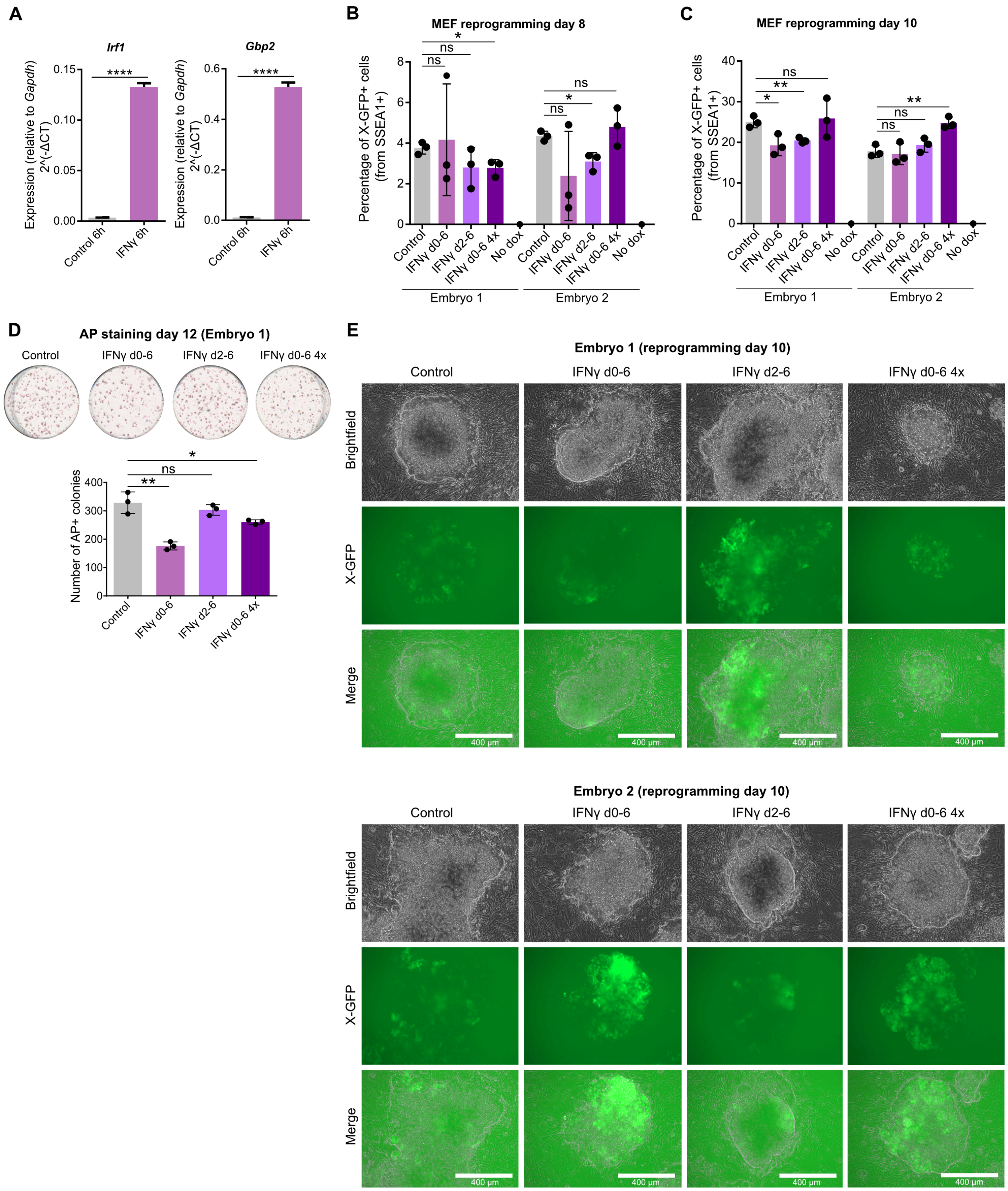

Fig. S4. *Legend on the next page.*

Fig. S4. Early activation of the IFNγ pathway during MEF reprogramming reduces colony number and does not enhance X-GFP reactivation. (**A**) RT-qPCR on mRNA for *Irf1* and *Gbp2* expression in control and IFNγ-treated reprogrammable female MEFs after 6 hours since reprogramming induction. Expression levels are normalized to *Gapdh* ($2^{-\Delta CT}$) (n = 3 technical replicates). Statistics (unpaired t-tests): **** = p<0.0001. Error bars represent SD. (**B,C**) X-GFP percentages from SSEA1+ cells at days 8 (B) and 10 (C) of female MEF reprogramming, in control and different IFNγ treatment conditions (d0-6, d2-6 and d0-6 with 4 times more cells seeded) (n = 3 technical replicates). MEFs from two different embryos were used. Statistics (unpaired t-tests): ns = non-significant; * = p<0.05; ** = p<0.01. Error bars represent SD. (**D**) Alkaline Phosphatase (AP) stainings on day 12 of reprogramming of MEFs derived from female embryo 1 in control and IFNγ treatment conditions, and counting of AP+ colonies (n=3 technical replicates). Statistics (unpaired t-tests): ns = non-significant; * = p<0.05; ** = p<0.01. Error bars represent SD. (**E**) Brightfield and fluorescent images (X-GFP) of live cells at day 10 of reprogramming (embryos 1 and 2) in control and different IFNγ-treatment conditions. Scale bar = 400 µm.

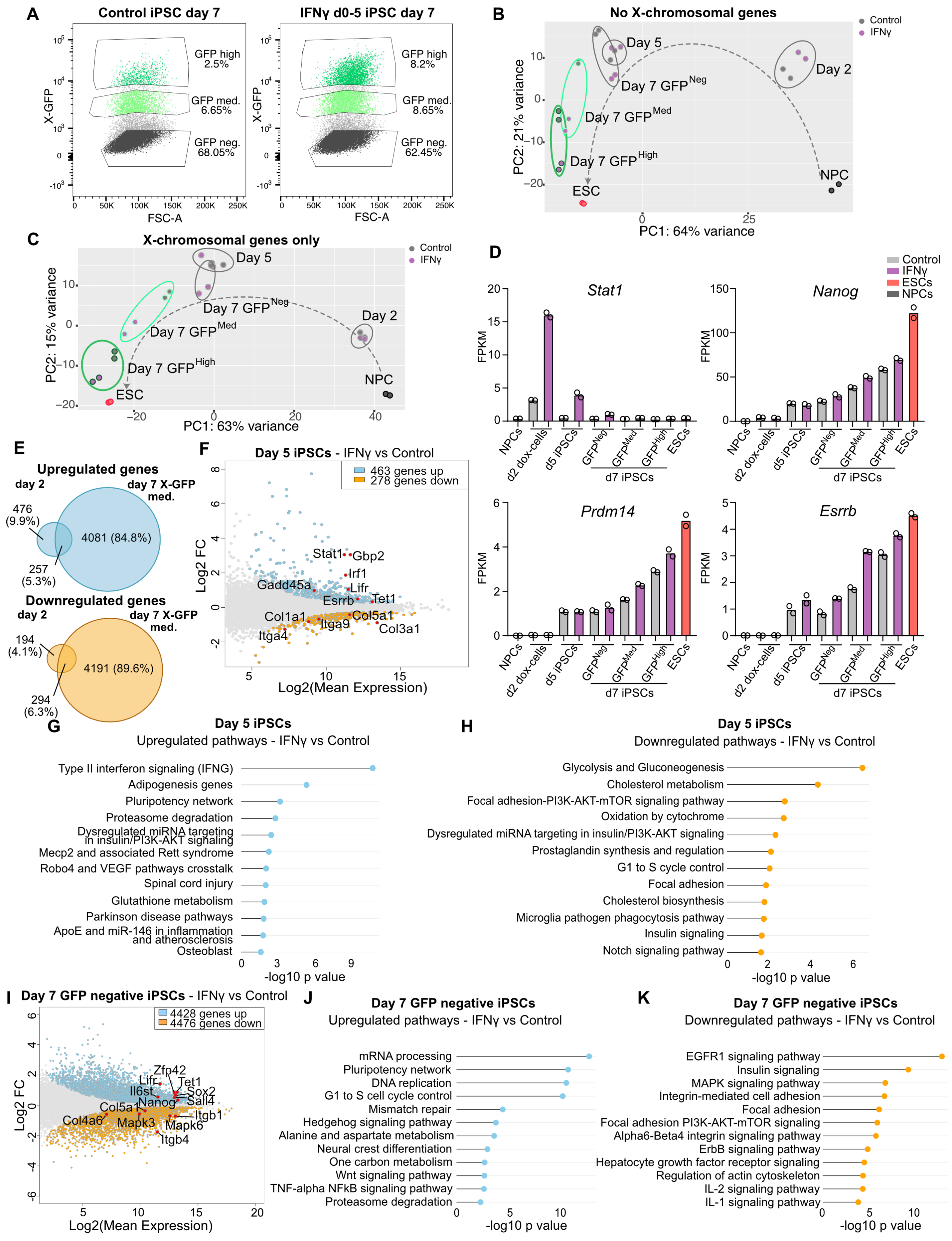

Fig. S5. *Legend on the next page.*

Fig. S5. Transcriptomic analysis of interferon γ pathway activation during iPSC reprogramming. Related to **Fig. 3**. (**A**) Flow cytometry plots of X-GFP expression (from SSEA1+ cells) in control and IFNγ-treated day 7 iPSCs. Gating shows sorted populations for RNA-sequencing. Average percentages between two independent reprogramming inductions are indicated for each population. (**B, C**) Principal component analysis of RNA-sequencing of NPCs, day 2, day 5, day 7 reprogramming populations and ESCs, in control and IFNγ treatment (day 0-5), representing the top 500 most variable autosomal genes only (B) and X-chromosomal genes only (C). (**D**) Expression (FPKM) of selected genes (*Stat1, Nanog, Prdm14* and *Esrrb*) in NPCs, ESCs, day 2, day 5 and day 7 reprogramming populations +/- IFNγ treatment (two RNA-sequencing replicates shown). (**E**) Venn diagram (using Venny 2.1.0) representing overlapping of upregulated and downregulated genes upon IFNγ treatment between day 2 dox-treated cells and day 7 X-GFP medium cells. (**F**) MA plot displaying transcriptomic changes of IFNγ vs control day 5 iPSCs (adjusted p value = 0.1). Upregulated genes are highlighted in light blue, downregulated genes are highlighted in orange. Selected genes are shown with points in red. (**G, H**) Upregulated (G) and downregulated (H) pathways in IFNγ vs control day 5 iPSCs (WikiPathways Mouse 2019) (adjusted p value = 0.1). (**I**) MA plot displaying transcriptomic changes of IFNγ vs control day 7 X-GFP negative iPSCs (adjusted p value threshold = 0.1). Upregulated genes are highlighted in light blue, downregulated genes are highlighted in orange. Selected genes are shown with points in red. (**J, K**) Upregulated (J) and downregulated (K) pathways in IFNγ vs control day 7 X-GFP negative iPSCs (WikiPathways Mouse 2019) (adjusted p value = 0.1).

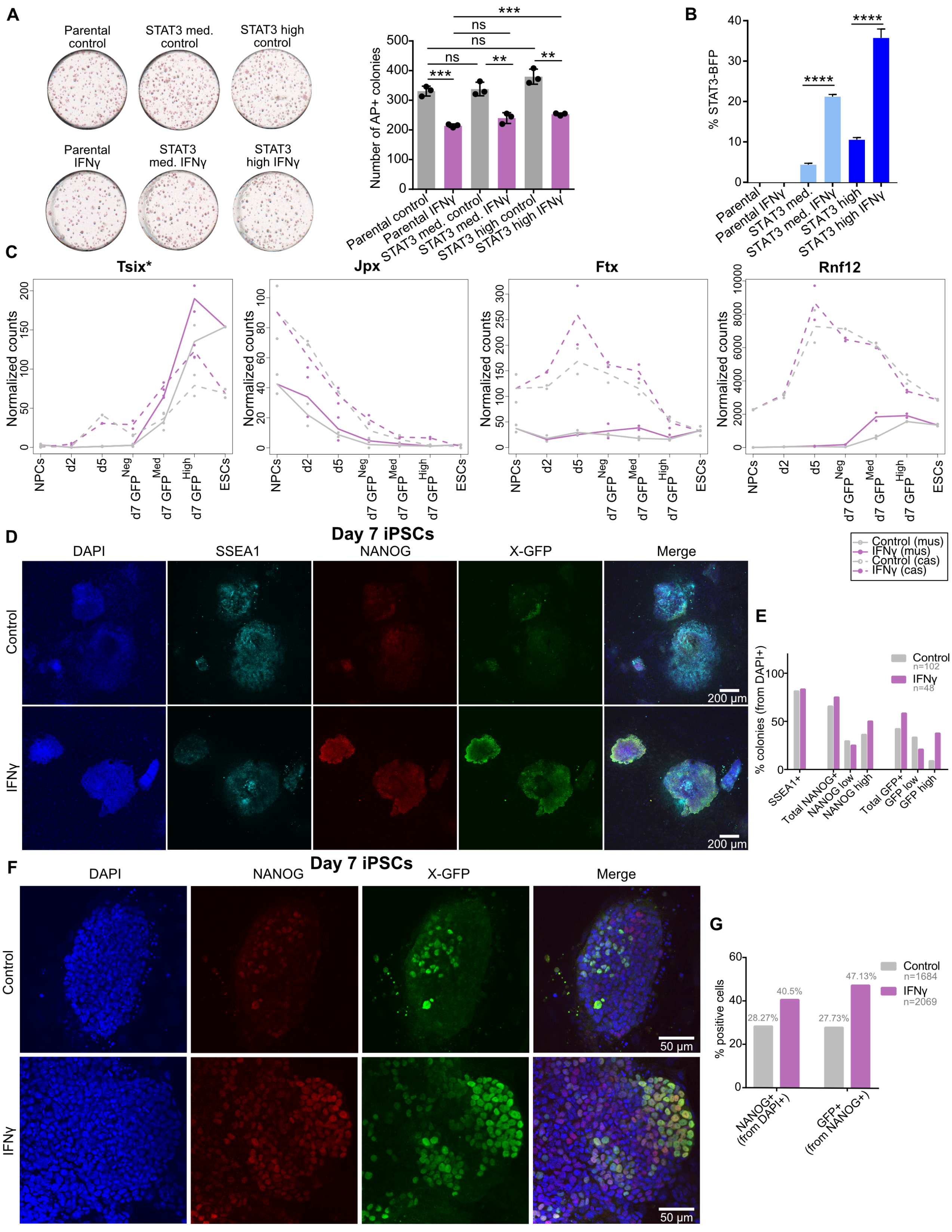

Fig. S6. *Legend on the next page.*

Fig. S6. Increased expression of NANOG and X-GFP in iPSC colonies upon early interferon γ treatment. Related to **Fig. 4**. (**A**) Alkaline Phosphatase (AP) stainings on day 10 of reprogramming in parental, STAT3-BFP medium and STAT3-BFP high cells +/- IFNγ treatment (d0-5) and counting of AP+ colonies (n=3 technical replicates). Statistics (unpaired t-tests): ns = non-significant; ** = $p<0.01$; *** = $p<0.001$. Error bars represent SD. (**B**) Percentages of STAT3-BFP+ cells at day 7 of reprogramming in parental, STAT3-BFP medium and STAT3-BFP high cells +/- IFNγ treatment (d0-5) (n=3 technical replicates). Statistics (unpaired t-tests): **** = $p<0.0001$. Error bars represent SD. (**C**) Expression (normalized counts) of genes of the X-inactivation center (*Tsix*, *Jpx*, *Ftx*, *Rnf12*) from X mus and X cas on NPCs, ESCs, day 2, day 5 and day 7 reprogramming populations +/- IFNγ treatment (two RNA-sequencing replicates shown). The * at *Tsix* indicates that the gene contains a truncation on the X-mus (*112*) and therefore cannot regulate *Xist* expression *in cis*. (**D**) Immunofluorescence (low magnification, 4x) for SSEA1, NANOG and X-GFP (active X chromosome) of day 7 reprogramming colonies +/- IFNγ treatment. Scale bar = 200 µm. (**E**) Percentages of SSEA1+, NANOG+ (low/high) and X-GFP+ (low/high) colonies from immunofluorescence in (D). The number (n) of counted colonies is indicated in the graph. NANOG+ or X-GFP+ colonies were scored as low or high if approximately less or more than half of the cells in the colony were positive for these markers, respectively. (**F**) Immunofluorescence (high magnification, 63x) for NANOG and X-GFP (active X chromosome) of day 7 reprogramming colonies +/- IFNγ treatment. Scale bar = 50 µm. (**G**) Percentages of NANOG+ and X-GFP+ (from NANOG+) cells from immunofluorescence in (F). The number (n) of counted cells is indicated in the graph.

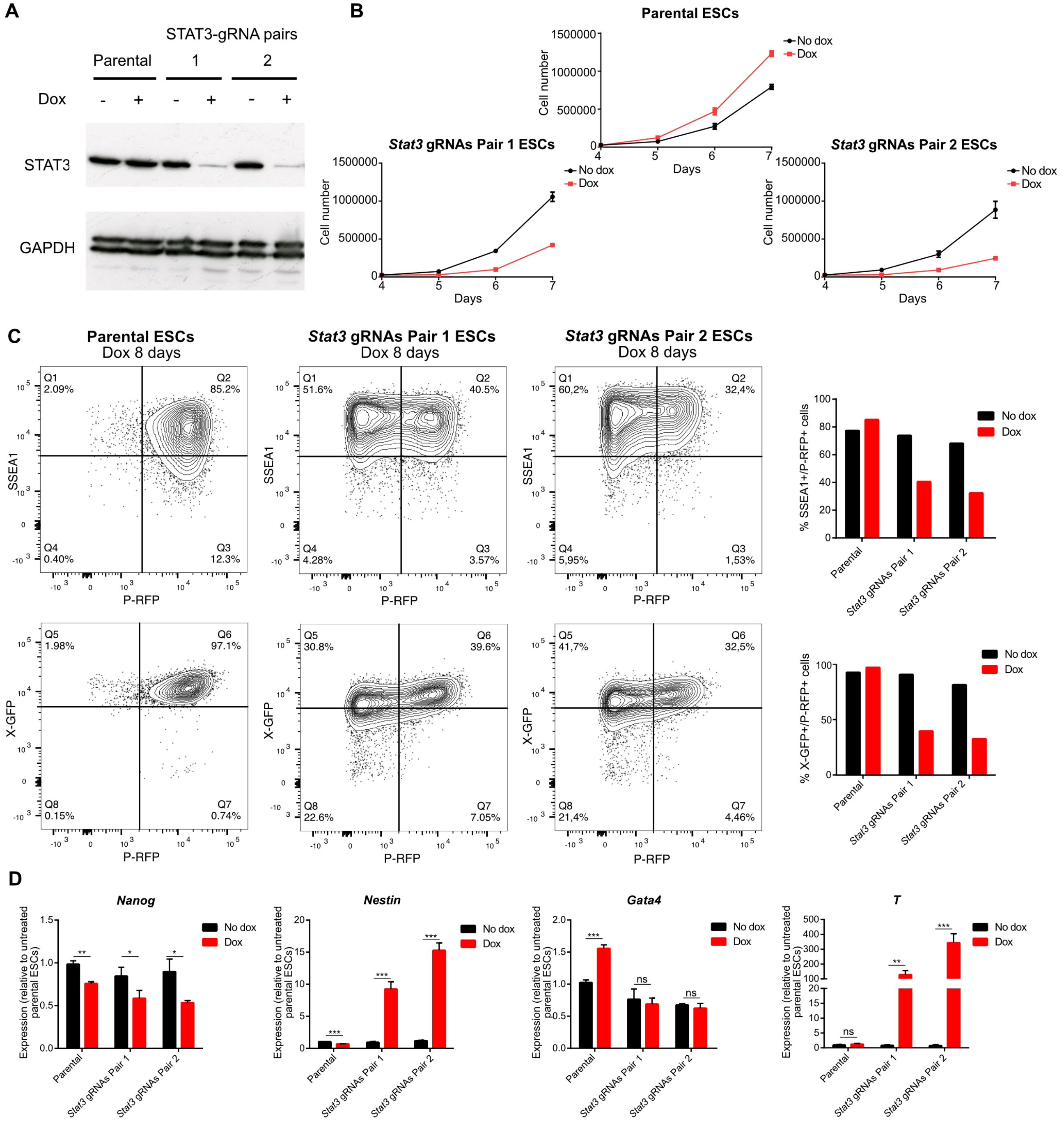

Fig. S7. Generation and characterization of *Stat3*-/- knockout ESC pools. Related to **Fig. 4**. (**A**) Western blotting of STAT3 in parental ESCs and ESCs infected with *Stat3*-targeting gRNAs (Pairs 1 and 2) +/- doxycycline treatment for 7 days (loading control: GAPDH). (**B**) Cell number measurement for days 4-7 +/- doxycycline treatment in parental ESCs and ESCs infected with *Stat3*-targeting gRNAs (Pairs 1 and 2) (25.000 cells seeded on day 4). (**C**) Flow cytometry plots on parental ESCs and ESCs infected with *Stat3*-targeting gRNAs (Pairs 1 and 2) upon doxycycline treatment for 8 days, showing expression of SSEA1/P-RFP (top) and X-GFP/P-RFP (bottom) and bar plots showing percentages of these double-positive populations in +/- doxycycline treatment conditions. (**D**) RT-qPCR on mRNA for *Nanog*, *Nestin*, *Gata4* and *T* on parental ESCs and ESCs infected with *Stat3*-targeting gRNAs (Pairs 1 and 2) +/- doxycycline treatment for 7 days (n=3 technical replicates). Statistics (unpaired t-tests): ns = non significant; * = p<0.05; ** = p<0.01; *** = p<0.001. Error bars represent SD.

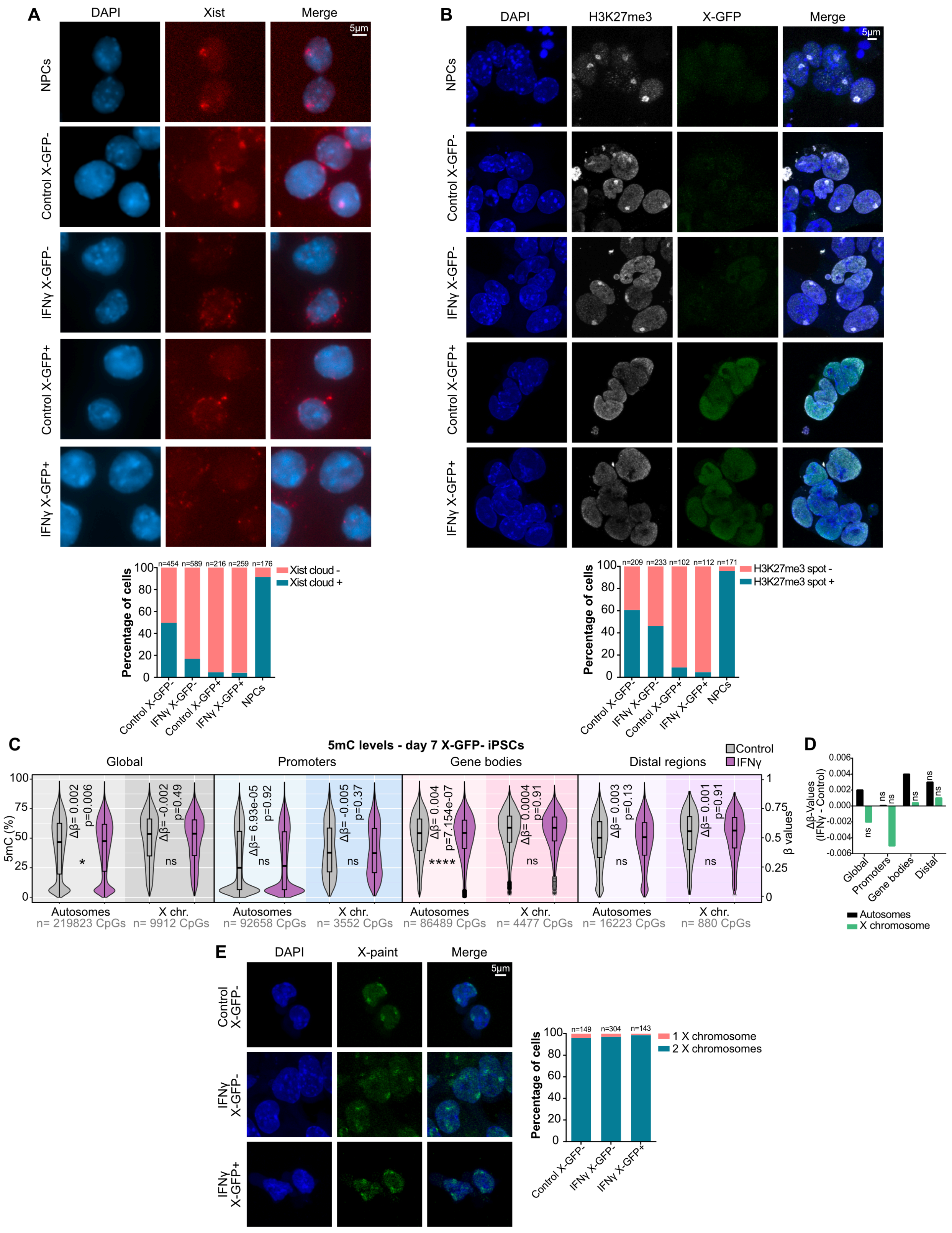

Fig. S8. *Legend on the next page.*

Fig. S8. Day 7 X-GFP- reprogramming cells show a reduction in Xist clouds and in H3K27me3 spots, and equal X-chromosomal DNA methylation levels. Related to **Fig. 4**. (**A**) RNA FISH showing Xist clouds on the inactive X chromosome (Sx9 probe) in NPCs and day 7 SSEA1+ X-GFP-negative/positive control/IFNγ iPSCs (scale bar = 5 μm) and quantification of Xist cloud-positive and -negative cells in all conditions. (**B**) H3K27me3 immunofluorescence in NPCs and day 7 SSEA1+ X-GFP negative/positive control/IFNγ iPSCs (scale bar = 5 μm) and quantification of H3K27me3 spot-positive and -negative cells in all conditions. (**C**) Analysis of 5mC levels (β-values) of CpGs on autosomes and X chromosome in day 7 X-GFP-negative iPSCs for control and IFNγ conditions, globally and divided by genomic distribution: promoters (<= 1kb from TSS), gene bodies and distal regions (number (n) of detected CpGs from each category is indicated on the bottom of the graphs). Δβ-values (mean β-value IFNγ - mean β-value control) and p values (comparison IFNγ vs control) are shown in the graphs. Statistics (unpaired t-tests): ns = non-significant; * = $p<0.05$; **** = $p<0.0001$. (**D**) Δβ-values for 5mC in day 7 X-GFP-negative iPSCs for each genomic region in autosomes and X chromosome (corresponding to analysis in (C)). Bars marked with “ns” correspond to non-significant changes from analysis in (C). (**E**) X-chromosome paint DNA FISH in control X-GFP-negative and IFNγ-treated (d0-5) X-GFP-negative and -positive day 7 iPSCs (scale bar = 5 μm) and quantification of cells with 1 or 2 X chromosomes.

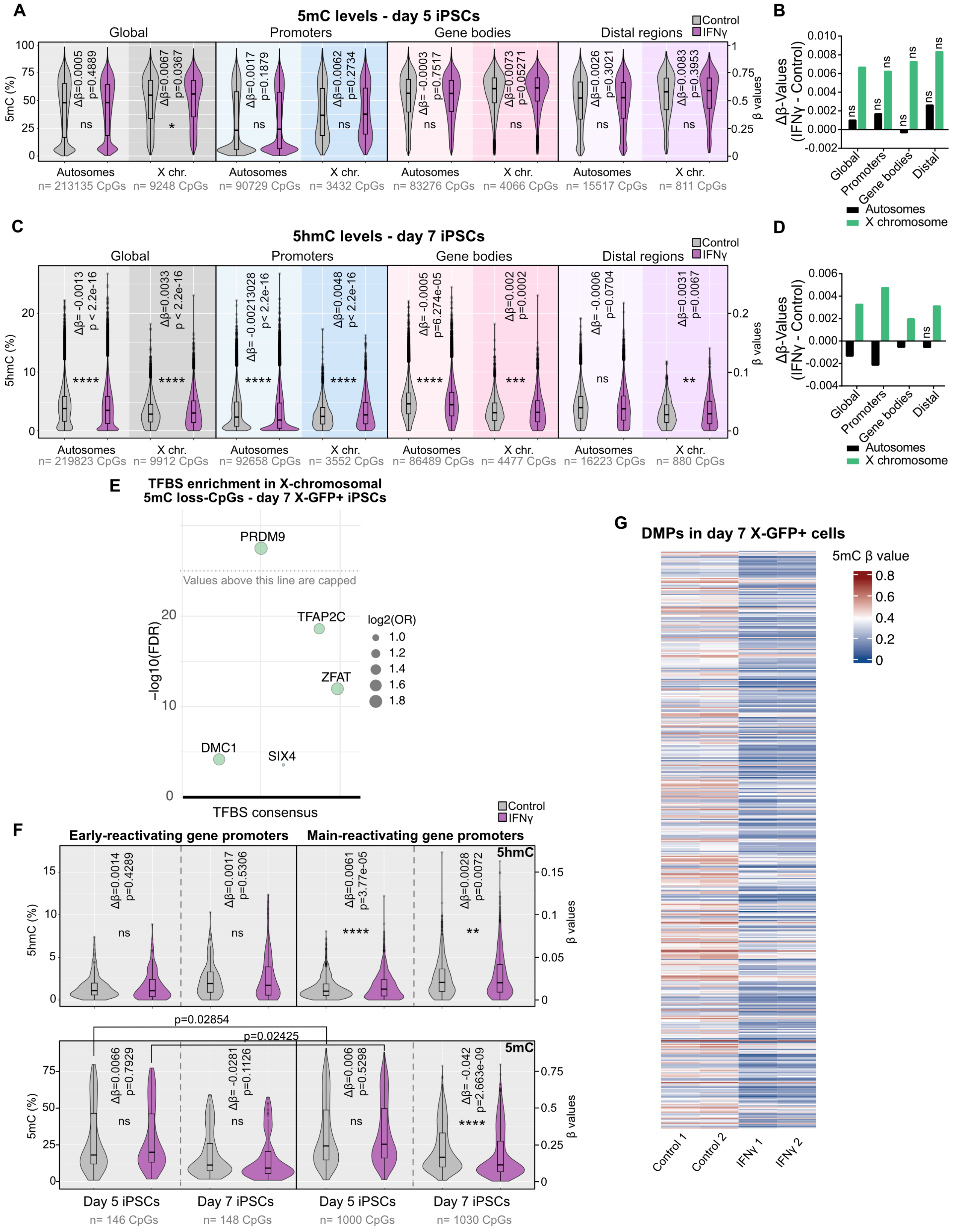

Fig. S9. *Legend on the next page.*

Fig. S9. Interferon γ treatment promotes TET-mediated DNA demethylation in cells undergoing reprogramming. Related to **Fig. 5**. (**A, C**) Analysis of 5mC levels in day 5 (A) or 5hmC levels in day 7 X-GFP+ cells (C) (β-values) of CpGs in autosomes and X chromosome for control and IFNγ (d0-5) conditions, globally and divided by genomic distribution: promoters (<= 1kb from TSS), gene bodies and distal regions (number (n) of detected CpGs from each category is indicated on the bottom of the graphs). Δβ-values (mean β-value IFNγ - mean β-value control) and p values (comparison IFNγ vs control) are shown in the graphs. Statistics: (unpaired t-tests): ns = non-significant; * = $p<0.05$; ** = $p<0.001$; **** = $p<0.0001$. (**B, D**) Δβ-values for 5mC in day 5 (B, corresponding to analysis in A) or 5hmC in day 7 X-GFP+ iPSCs (D, corresponding to analysis in C) for each genomic region in autosomes and X chromosomes. Bars marked with "ns" correspond to non-significant changes from analysis in (A) or (C). (**E**) Transcription factor binding site (TFBS) enrichment analysis on differentially methylated X-chromosomal CpGs (DMPs, logFC<(-0.1), $p<0.01$, n=468 CpGs) which lose methylation upon IFNγ treatment compared to control in day 7 X-GFP+ iPSCs. -log10(FDR) capped values are above 25. (**F**) Analysis of 5mC and 5hmC levels (β-values) of CpGs in early and main X-reactivating gene promoters at day 5 and day 7 X-GFP+ iPSCs for control and IFNγ conditions (gene lists were obtained from (*24*)). Number (n) of detected CpGs for each category and time point is indicated on the bottom of the graphs. Δβ-values and p values (comparison IFNγ vs control) are shown in the graphs. Statistics (unpaired t-tests): ns = non-significant; ** = $p<0.001$; **** = $p<0.0001$. (**G**) Heatmap showing 5mC levels (β-values) of all X-chromosomal differentially methylated CpGs (n=470 DMPs, logFC cutoff = +/-0.1, $p<0.01$) sorted by chromosome position.

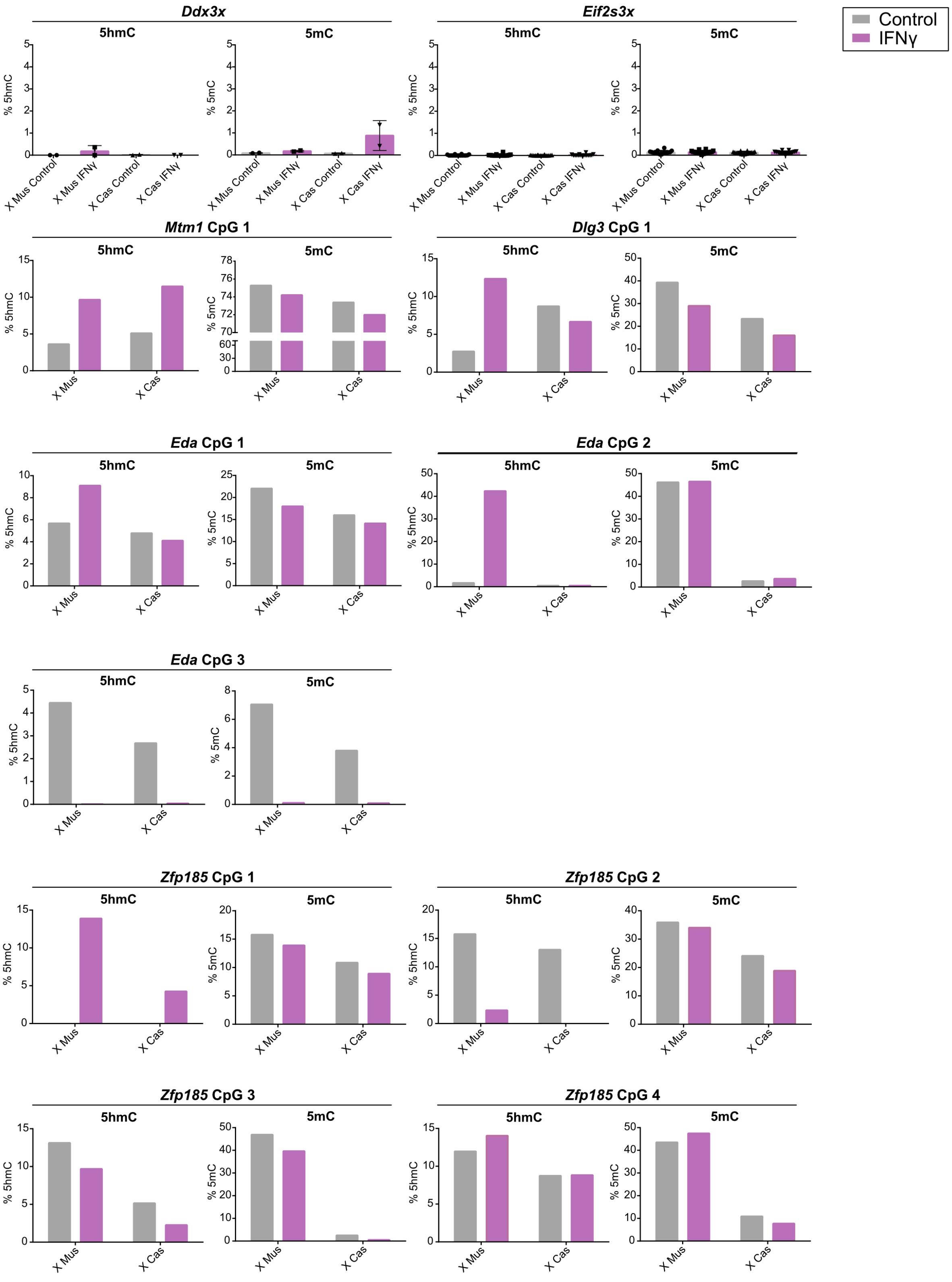

Fig. S10. *Legend on the next page.*

Fig. S10. Allele-specific (hydroxy)methylation analysis by targeted amplicon oxidative BS-sequencing. Related to **Fig. 5**. Allele-specific analysis of 5mC and 5hmC percentages (relative to total C) at day 5 of reprogramming in control and IFNγ-treated samples, in specific promoter *loci* surrounding CpGs from X-reactivating genes that were found as differentially hydroxymethylated on day 5 or 7 in Fig. 5, including escapee gene controls. Analysed loci contained promoter regions of the escapee genes *Ddx3x* (2 CpGs) and *Eif2s3x* (16 CpGs) (plotted together because of low variability), and the X-reactivating genes *Mtm1* (1 CpG), *Dlg3* (1 CpG), *Eda* (3 CpGs) and *Zfp185* (4 CpGs) (plotted separately because of high variability).

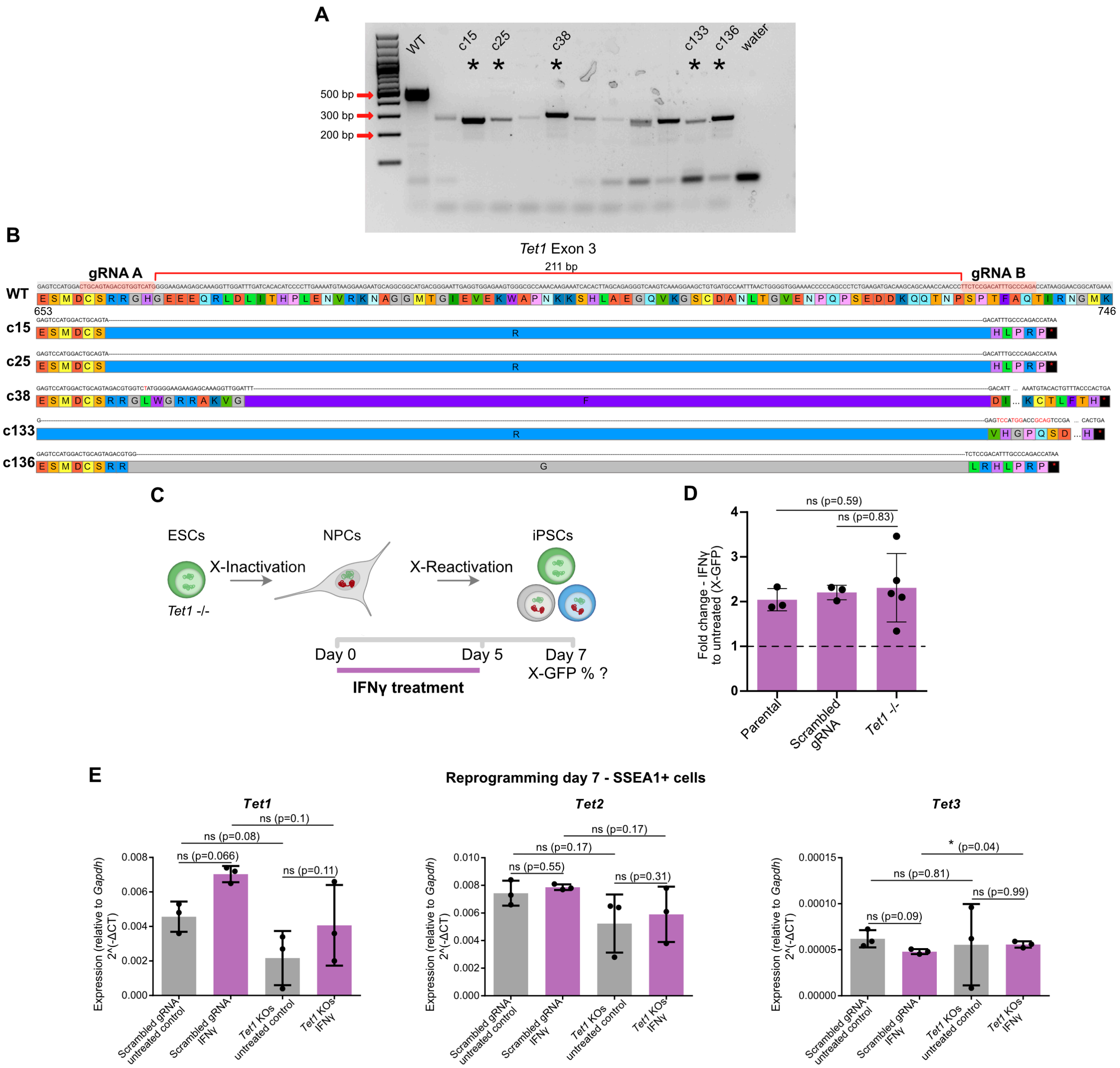

Fig. S11. *Legend on the next page.*

Fig. S11. Absence of TET1 does not impede IFNγ-mediated enhanced X-GFP reactivation at day 7 of reprogramming. Related to **Fig. 5**. (**A**) PCR on genomic DNA of parental (WT) and *Tet1*-/- clones showing an around 200 bp deletion in exon 3. The asterisk marks the clones used for further experiments. (**B**) Schematic representation of Sanger sequencing (and amino acid equivalence) of PCR products from genomic DNA in the parental clone (WT) and 5 *Tet1*-/- clones used for the experiment, which showed a premature STOP codon (represented in black). (**C**) Experimental design for (D): *Tet1-/-*, parental and scrambled gRNA control ESCs were differentiated into NPCs and then reprogrammed into iPSCs in the presence or absence of IFNγ (day 0-5). X-GFP percentages (from SSEA1+ cells) were measured by flow cytometry at day 7 of reprogramming. 3 clones from the parental cell line, 3 clones containing a scrambled gRNA and 5 *Tet1 -/-* clones were used, including three technical replicates for each clone. (**D**) Fold change of percentage of X-GFP+ cells (from SSEA1+ cells) in IFNγ-treated cells compared to untreated controls on day 7 of reprogramming, measured by flow cytometry. Bars represent the average X-GFP fold change (IFNγ vs control) for clones with the same genotype, listed in (C). Each dot represents the mean of three technical replicates for each clone. Statistics (unpaired t-tests): ns = non-significant. Error bars represent SD. (**E**) RT-qPCR on mRNA for *Tet1*, *Tet2* and *Tet3* expression in day 7 SSEA1+ cells (+/- IFNγ treatment day 0-5) from 3 scrambled and 3 *Tet1*-/- clones. Expression levels are normalized to *Gapdh* ($2^{-\Delta CT}$). Statistics (paired t-tests between control and treatment within clones, unpaired t-tests for comparisons between different clones): ns = non-significant; * = $p<0.05$. Error bars represent SD.

Supplemental Tables Description

Supplemental Table S1. MAGeGK gene summary for CRISPR screen comparisons. Related to **Fig. 1** and **fig. S1**. Statistical comparisons for each gene in "non-pluripotent vs NPCs", "early pluripotent vs NPCs", "late pluripotent vs NPCs", "early vs non-pluripotent" and "late vs early pluripotent".

Supplemental Table S2. Lists of genes and pathways for CRISPR screen comparisons. Related to **Fig. 1** and **fig. S1**. Gene lists for each category and pathways ("WikiPathways mouse 2019") corresponding to each of them: essentialome, repressors of colony formation, drivers and repressors of early pluripotency, drivers and repressors of late pluripotency and X-reactivation.

Supplemental Table S3. **DESeq2 and pathway analysis from RNA-sequencing experiments.** Related to **Fig. 3** and **fig. S5**. Differential gene expression analysis for each reprogramming timepoint (IFNγ vs control) and pathways ("WikiPathways mouse 2019") associated to them (day 2, day 5, day 7 X-GFP negative, day 7 X-GFP medium, day 7 X-GFP high), and allelic ratio for X-linked genes in ESCs, NPCs and each reprogramming population.

Supplemental Table S4. DNA methylation: DMPs, TFBS enrichment, gene lists and pathway analysis. Related to **Fig. 5, fig. S8** and **fig**. **S9**. Differentially methylated CpGs (DMPs) for 5mC at days 5 and day 7 (X-GFP+) iPSCs (IFNγ vs control); overlap of upregulated genes in day 7 X-GFP+ cells by RNA-seq and genes with lower promoter 5mC levels in day 7 X-GFP+ cells and pathways (WikiPathways mouse 2019) associated to them; lists of X-reactivating, "early" and "main" X-reactivating, and escapee genes obtained from (24), SeSAMe TFBS enrichment (based on ChIP-seq data from Cistrome/ENCODE databases) at days 5 and 7 for CpGs losing 5mC globally, and for CpGs losing 5mC on the X chromosome at day 7.

Supplemental Table S5. Resources: oligonucleotides, antibodies, molecules for pathway validation, cell lines and softwares used in this study.

Supplemental Table S6. Source data for figure panels.

REFERENCES AND NOTES

1. K. Hochedlinger, R. Jaenisch, Induced pluripotency and epigenetic reprogramming. *Cold Spring Harb. Perspect. Biol.* **7**, a019448 (2015).

2. W. Reik, M. A. Surani, Germline and pluripotent stem cells. *Cold Spring Harb. Perspect. Biol.* **7**, a019422 (2015).

3. A. Panda, J. J. Zylicz, V. Pasque, New insights into x-chromosome reactivation during reprogramming to pluripotency. *Cells* **9**, 2706 (2020).

4. T. Mattimoe, B. Payer, The compleX balancing act of controlling X-chromosome dosage and how it impacts mammalian germline development. *Biochem. J.* **480**, 521–537 (2023).

5. W. Mak, T. B. Nesterova, M. de Napoles, R. Appanah, S. Yamanaka, A. P. Otte, N. Brockdorff, Reactivation of the paternal X chromosome in early mouse embryos. *Science* **303**, 666–669 (2004).

6. I. Okamoto, A. P. Otte, C. D. Allis, D. Reinberg, E. Heard, Epigenetic dynamics of imprinted X inactivation during early mouse development. *Science* **303**, 644–649 (2004).

7. M. Borensztein, I. Okamoto, L. Syx, G. Guilbaud, C. Picard, K. Ancelin, R. Galupa, P. Diabangouaya, N. Servant, E. Barillot, A. Surani, M. Saitou, C.-J. Chen, K. Anastassiadis, E. Heard, Contribution of epigenetic landscapes and transcription factors to X-chromosome reactivation in the inner cell mass. *Nat. Commun.* **8**, 1297 (2017).

8. M. Sugimoto, K. Abe, X chromosome reactivation initiates in nascent primordial germ cells in mice. *PLOS Genet.* **3**, e116 (2007).

9. S. M. Chuva de Sousa Lopes, K. Hayashi, T. C. Shovlin, W. Mifsud, M. A. Surani, A. McLaren, X chromosome activity in mouse XX primordial germ cells. *PLOS Genet.* **4**, e30 (2008).

10. A. Mallol, M. Guirola, B. Payer, PRDM14 controls X-chromosomal and global epigenetic reprogramming of H3K27me3 in migrating mouse primordial germ cells. *Epigenetics Chromatin* **12**, 38 (2019).

11. J. Severino, M. Bauer, T. Mattimoe, N. Arecco, L. Cozzuto, P. Lorden, N. Hamada, Y. Nosaka, S. I. Nagaoka, P. Audergon, A. Tarruell, H. Heyn, K. Hayashi, M. Saitou, B. Payer, Controlled X-chromosome dynamics defines meiotic potential of female mouse in vitro germ cells. *EMBO J.* **41**, e109457 (2022).

12. C. Roidor, L. Syx, E. Beyne, D. Zielinski, A. Teissandier, C. Lee, M. Walter, N. Servant, K. Chebli, D. Bourc'his, M. Azim Surani, M. Borensztein, Spatio-temporal X-linked gene reactivation and site-specific retention of epigenetic silencing in the mouse germline. bioRxiv 2023.04.25.532252 [Preprint] (2023). https://doi.org/10.1101/2023.04.25.532252.

13. P. Navarro, I. Chambers, V. Karwacki-Neisius, C. Chureau, C. Morey, C. Rougeulle, P. Avner, Molecular coupling of Xist regulation and pluripotency. *Science* **321**, 1693–1695 (2008).

14. P. Navarro, M. Moffat, N. P. Mullin, I. Chambers, The X-inactivation trans-activator Rnf12 is negatively regulated by pluripotency factors in embryonic stem cells. *Hum. Genet.* **130**, 255–264 (2011).

15. B. Payer, M. Rosenberg, M. Yamaji, Y. Yabuta, M. Koyanagi-Aoi, K. Hayashi, S. Yamanaka, M. Saitou, J. T. Lee, Tsix RNA and the germline factor, PRDM14, link X reactivation and stem cell reprogramming. *Mol. Cell* **52**, 805–818 (2013).

16. B. Payer, J. T. Lee, Coupling of X-chromosome reactivation with the pluripotent stem cell state. *RNA Biol.* **11**, 798–807 (2014).

17. I. Jonkers, T. S. Barakat, E. M. Achame, K. Monkhorst, A. Kenter, E. Rentmeester, F. Grosveld, J. A. Grootegoed, J. Gribnau, RNF12 is an X-Encoded dose-dependent activator of X chromosome inactivation. *Cell* **139**, 999–1011 (2009).

18. D. Tian, S. Sun, J. T. Lee, The long noncoding RNA, Jpx, is a molecular switch for X chromosome inactivation. *Cell* **143**, 390–403 (2010).

19. C. Gontan, E. M. Achame, J. Demmers, T. S. Barakat, E. Rentmeester, W. van IJcken, J. A. Grootegoed, J. Gribnau, RNF12 initiates X-chromosome inactivation by targeting REX1 for degradation. *Nature* **485**, 386–390 (2012).

20. S. Sun, B. C. Del Rosario, A. Szanto, Y. Ogawa, Y. Jeon, J. T. Lee, Jpx RNA activates Xist by evicting CTCF. *Cell* **153**, 1537–1551 (2013).

21. R. A. F. Gjaltema, T. Schwämmle, P. Kautz, M. Robson, R. Schöpflin, L. Ravid Lustig, L. Brandenburg, I. Dunkel, C. Vechiatto, E. Ntini, V. Mutzel, V. Schmiedel, A. Marsico, S. Mundlos, E. G. Schulz, Distal and proximal cis-regulatory elements sense X chromosome dosage and developmental state at the Xist locus. *Mol. Cell* **82**, 190–208.e17 (2022).

22. N. Maherali, R. Sridharan, W. Xie, J. Utikal, S. Eminli, K. Arnold, M. Stadtfeld, R. Yachechko, J. Tchieu, R. Jaenisch, K. Plath, K. Hochedlinger, Directly reprogrammed fibroblasts show global epigenetic remodeling and widespread tissue contribution. *Cell Stem Cell* **1**, 55–70 (2007).

23. V. Pasque, J. Tchieu, R. Karnik, M. Uyeda, A. Sadhu Dimashkie, D. Case, B. Papp, G. Bonora, S. Patel, R. Ho, R. Schmidt, R. McKee, T. Sado, T. Tada, A. Meissner, K. Plath, X chromosome reactivation dynamics reveal stages of reprogramming to pluripotency. *Cell* **159**, 1681–1697 (2014).

24. M. Bauer, E. Vidal, E. Zorita, N. Üresin, S. F. Pinter, G. J. Filion, B. Payer, Chromosome compartments on the inactive X guide TAD formation independently of transcription during X-reactivation. *Nat. Commun.* **12**, 3499 (2021).

25. A. Janiszewski, I. Talon, J. Chappell, S. Collombet, J. Song, N. de Geest, S. K. To, G. Bervoets, O. Marin-Bejar, C. Provenzano, L. Vanheer, J.-C. Marine, F. Rambow, V. Pasque, Dynamic reversal of random X-Chromosome inactivation during iPSC reprogramming. *Genome Res.* **29**, 1659–1672 (2019).

26. I. Talon, A. Janiszewski, B. Theeuwes, T. Lefevre, J. Song, G. Bervoets, L. Vanheer, N. De Geest, S. Poovathingal, R. Allsop, J.-C. Marine, F. Rambow, T. Voet, V. Pasque, Enhanced chromatin accessibility contributes to X chromosome dosage compensation in mammals. *Genome Biol.* **22**, 302 (2021).

27. S. F. Generoso, M. V. Neguembor, E. A. Hershberg, R. I. Sadreyev, K. Kurimoto, Y. Yabuta, R. Ricci, P. Audergon, M. Bauer, M. Saitou, K. Hochedlinger, B. J. Beliveau, M. P. Cosma, J. T. Lee, B. Payer, Cohesin controls X chromosome structure remodeling and X-reactivation during mouse iPSC-reprogramming. *Proc. Natl. Acad. Sci. U.S.A.* **120**, e2213810120 (2023).

28. C. Bock, P. Datlinger, F. Chardon, M. A. Coelho, M. B. Dong, K. A. Lawson, T. Lu, L. Maroc, T. M. Norman, B. Song, G. Stanley, S. Chen, M. Garnett, W. Li, J. Moffat, L. S. Qi, R. S. Shapiro, J. Shendure, J. S. Weissman, X. Zhuang, High-content CRISPR screening. *Nat. Rev. Methods Primers* **2**, 8 (2022).

29. R. M. J. Genga, E. M. Kernfeld, K. M. Parsi, T. J. Parsons, M. J. Ziller, R. Maehr, Single-cell RNA-sequencing-based CRISPRi screening resolves molecular drivers of early human endoderm development. *Cell Rep.* **27**, 708–718.e10 (2019).

30. Q. V. Li, G. Dixon, N. Verma, B. P. Rosen, M. Gordillo, R. Luo, C. Xu, Q. Wang, C.-L. Soh, D. Yang, M. Crespo, A. Shukla, Q. Xiang, F. Dündar, P. Zumbo, M. Witkin, R. Koche, D. Betel, S. Chen, J. Massagué, R. Garippa, T. Evans, M. A. Beer, D. Huangfu, Genome-scale screens identify JNK-JUN signaling as a barrier for pluripotency exit and endoderm differentiation. *Nat. Genet.* **51**, 999–1010 (2019).

31. M. Li, J. S. L. Yu, K. Tilgner, S. H. Ong, H. Koike-Yusa, K. Yusa, Genome-wide CRISPR-KO screen uncovers mTORC1-mediated Gsk3 regulation in naive pluripotency maintenance and dissolution. *Cell Rep.* **24**, 489–502 (2018).

32. J. A. Hackett, Y. Huang, U. Günesdogan, K. A. Gretarsson, T. Kobayashi, M. A. Surani, Tracing the transitions from pluripotency to germ cell fate with CRISPR screening. *Nat. Commun.* **9**, 4292 (2018).

33. A. Yilmaz, M. Peretz, A. Aharony, I. Sagi, N. Benvenisty, Defining essential genes for human pluripotent stem cells by CRISPR-Cas9 screening in haploid cells. *Nat. Cell Biol.* **20**, 610–619 (2018).

34. R. J. Ihry, M. R. Salick, D. J. Ho, M. Sondey, S. Kommineni, S. Paula, J. Raymond, B. Henry, E. Frias, Q. Wang, K. A. Worringer, C. Ye, C. Russ, J. S. Reece-Hoyes, R. C. Altshuler, R. Randhawa, Z. Yang, G. McAllister, G. R. Hoffman, R. Dolmetsch, A. Kaykas, Genome-scale CRISPR screens identify human pluripotency-specific genes. *Cell Rep.* **27**, 616–630.e6 (2019).

35. B. Mair, J. Tomic, S. N. Masud, P. Tonge, A. Weiss, M. Usaj, A. H. Y. Tong, J. J. Kwan, K. R. Brown, E. Titus, M. Atkins, K. S. K. Chan, L. Munsie, A. Habsid, H. Han, M. Kennedy, B. Cohen, G.

Keller, J. Moffat, Essential gene profiles for human pluripotent stem cells identify uncharacterized genes and substrate dependencies. *Cell Rep.* **27**, 599–615.e12 (2019).

36. D. Seruggia, M. Oti, P. Tripathi, M. C. Canver, L. LeBlanc, D. C. Di Giammartino, M. J. Bullen, C. M. Nefzger, Y. B. Y. Sun, R. Farouni, J. M. Polo, L. Pinello, E. Apostolou, J. Kim, S. H. Orkin, P. P. Das, TAF5L and TAF6L maintain self-renewal of embryonic stem cells via the MYC regulatory network. *Mol. Cell* **74**, 1148–1163.e7 (2019).

37. A. J. Collier, A. Bendall, C. Fabian, A. A. Malcolm, K. Tilgner, C. I. Semprich, K. Wojdyla, P. S. Nisi, K. Kishore, V. N. Roamio Franklin, B. Mirshekar-Syahkal, C. D'Santos, K. Plath, K. Yusa, P. J. Rugg-Gunn, Genome-wide screening identifies Polycomb repressive complex 1.3 as an essential regulator of human naïve pluripotent cell reprogramming. *Sci. Adv.* **8**, eabk0013 (2022).

38. J. Yang, S. S. Rajan, M. J. Friedrich, G. Lan, X. Zou, H. Ponstingl, D. A. Garyfallos, P. Liu, A. Bradley, E. Metzakopian, Genome-scale CRISPRa screen identifies novel factors for cellular reprogramming. *Stem Cell Rep.* **12**, 757–771 (2019).

39. D. F. Kaemena, M. Yoshihara, M. Beniazza, J. Ashmore, S. Zhao, M. Bertenstam, V. Olariu, S. Katayama, K. Okita, S. R. Tomlinson, K. Yusa, K. Kaji, B1 SINE-binding ZFP266 impedes mouse iPSC generation through suppression of chromatin opening mediated by reprogramming factors. *Nat. Commun.* **14**, 488 (2023).

40. O. Genolet, A. A. Monaco, I. Dunkel, M. Boettcher, E. G. Schulz, Identification of X-chromosomal genes that drive sex differences in embryonic stem cells through a hierarchical CRISPR screening approach. *Genome Biol.* **22**, 110 (2021).

41. L. R. Lustig, A. S. Kumar, T. Schwämmle, I. Dunkel, G. Noviello, R. Weigert, G. Pacini, R. Buschow, A. Ghauri, M. Stötzel, L. Wittler, A. Meissner, E. G. Schulz, GATA transcription factors drive initial Xist upregulation after fertilization through direct activation of a distal enhancer element. bioRxiv 2022.08.02.502458 [Preprint] (2022). https://doi.org/10.1101/2022.08.02.502458.

42. K. Sakurai, I. Talukdar, V. S. Patil, J. Dang, Z. Li, K.-Y. Chang, C.-C. Lu, V. Delorme-Walker, C. Dermardirossian, K. Anderson, D. Hanein, C.-S. Yang, D. Wu, Y. Liu, T. M. Rana, Kinome-wide

functional analysis highlights the role of cytoskeletal remodeling in somatic cell reprogramming. *Cell Stem Cell* **14**, 523–534 (2014).

43. H. Qin, A. Diaz, L. Blouin, R. J. Lebbink, W. Patena, P. Tanbun, E. M. LeProust, M. T. McManus, J. S. Song, M. Ramalho-Santos, Systematic identification of barriers to human iPSC generation. *Cell* **158**, 449–461 (2014).

44. S. Cheloufi, U. Elling, B. Hopfgartner, Y. L. Jung, J. Murn, M. Ninova, M. Hubmann, A. I. Badeaux, C. Euong Ang, D. Tenen, D. J. Wesche, N. Abazova, M. Hogue, N. Tasdemir, J. Brumbaugh, P. Rathert, J. Jude, F. Ferrari, A. Blanco, M. Fellner, D. Wenzel, M. Zinner, S. E. Vidal, O. Bell, M. Stadtfeld, H. Y. Chang, G. Almouzni, S. W. Lowe, J. Rinn, M. Wernig, A. Aravin, Y. Shi, P. J. Park, J. M. Penninger, J. Zuber, K. Hochedlinger, The histone chaperone CAF-1 safeguards somatic cell identity. *Nature* **528**, 218–224 (2015).

45. M. Borkent, B. D. Bennett, B. Lackford, O. Bar-Nur, J. Brumbaugh, L. Wang, Y. Du, D. C. Fargo, E. Apostolou, S. Cheloufi, N. Maherali, S. J. Elledge, G. Hu, K. Hochedlinger, A serial shRNA screen for roadblocks to reprogramming identifies the protein modifier SUMO2. *Stem Cell Rep.* **6**, 704–716 (2016).

46. H. Kagawa, R. Shimamoto, S.-I. Kim, F. Oceguera-Yanez, T. Yamamoto, T. Schroeder, K. Woltjen, OVOL1 influences the determination and expansion of iPSC reprogramming intermediates. *Stem Cell Rep.* **12**, 319–332 (2019).

47. M. D. Schertzer, E. Thulson, K. C. A. Braceros, D. M. Lee, E. R. Hinkle, R. M. Murphy, S. O. Kim, E. C. M. Vitucci, J. M. Calabrese, A piggyBac-based toolkit for inducible genome editing in mammalian cells. *RNA* **25**, 1047–1058 (2019).

48. K. Tzelepis, H. Koike-Yusa, E. De Braekeleer, Y. Li, E. Metzakopian, O. M. Dovey, A. Mupo, V. Grinkevich, M. Li, M. Mazan, M. Gozdecka, S. Ohnishi, J. Cooper, M. Patel, T. McKerrell, B. Chen, A. F. Domingues, P. Gallipoli, S. Teichmann, H. Ponstingl, U. McDermott, J. Saez-Rodriguez, B. J. P. Huntly, F. Iorio, C. Pina, G. S. Vassiliou, K. Yusa, A CRISPR dropout screen identifies genetic vulnerabilities and therapeutic targets in acute myeloid leukemia. *Cell Rep.* **17**, 1193–1205 (2016).

49. E. Chantzoura, S. Skylaki, S. Menendez, S.-I. Kim, A. Johnsson, S. Linnarsson, K. Woltjen, I. Chambers, K. Kaji, Reprogramming roadblocks are system dependent. *Stem Cell Rep.* **5**, 350–364 (2015).

50. G. Schiebinger, J. Shu, M. Tabaka, B. Cleary, V. Subramanian, A. Solomon, J. Gould, S. Liu, S. Lin, P. Berube, L. Lee, J. Chen, J. Brumbaugh, P. Rigollet, K. Hochedlinger, R. Jaenisch, A. Regev, E. S. Lander, Optimal-transport analysis of single-cell gene expression identifies developmental trajectories in reprogramming. *Cell* **176**, 928–943.e22 (2019).

51. S. Ruiz, A. D. Panopoulos, A. Herrerías, K.-D. Bissig, M. Lutz, W. T. Berggren, I. M. Verma, J. C. Izpisua Belmonte, A high proliferation rate is required for cell reprogramming and maintenance of human embryonic stem cell identity. *Curr. Biol.* **21**, 45–52 (2011).

52. Y. Pei, L. Yue, W. Zhang, Y. Wang, B. Wen, L. Zhong, J. Xiang, J. Li, S. Zhang, H. Wang, H. Mu, Q. Wei, J. Han, Improvement in mouse iPSC induction by Rab32 reveals the importance of lipid metabolism during reprogramming. *Sci. Rep.* **5**, 16539 (2015).

53. J. K. Ichida, J. Tcw, L. A. Williams, A. C. Carter, Y. Shi, M. T. Moura, M. Ziller, S. Singh, G. Amabile, C. Bock, A. Umezawa, L. L. Rubin, J. E. Bradner, H. Akutsu, A. Meissner, K. Eggan, Notch inhibition allows oncogene-independent generation of iPS cells. *Nat. Chem. Biol.* **10**, 632–639 (2014).

54. J. Chen, J. Liu, J. Yang, Y. Chen, J. Chen, S. Ni, H. Song, L. Zeng, K. Ding, D. Pei, BMPs functionally replace Klf4 and support efficient reprogramming of mouse fibroblasts by Oct4 alone. *Cell Res.* **21**, 205–212 (2011).

55. I. Neganova, V. Chichagova, L. Armstrong, M. Lako, A critical role for p38MAPK signalling pathway during reprogramming of human fibroblasts to iPSCs. *Sci. Rep.* **7**, 41693 (2017).

56. A. Marson, R. Foreman, B. Chevalier, S. Bilodeau, M. Kahn, R. A. Young, R. Jaenisch, Wnt signaling promotes reprogramming of somatic cells to pluripotency. *Cell Stem Cell* **3**, 132–135 (2008).

57. J. K. Ichida, J. Blanchard, K. Lam, E. Y. Son, J. E. Chung, D. Egli, K. M. Loh, A. C. Carter, F. P. Di Giorgio, K. Koszka, D. Huangfu, H. Akutsu, D. R. Liu, L. L. Rubin, K. Eggan, A small-molecule

inhibitor of tgf-Beta signaling replaces sox2 in reprogramming by inducing nanog. *Cell Stem Cell* **5**, 491–503 (2009).

58. J. Yang, A. L. van Oosten, T. W. Theunissen, G. Guo, J. C. R. Silva, A. Smith, Stat3 activation is limiting for reprogramming to ground state pluripotency. *Cell Stem Cell* **7**, 319–328 (2010).

59. A. L. van Oosten, Y. Costa, A. Smith, J. C. R. Silva, JAK/STAT3 signalling is sufficient and dominant over antagonistic cues for the establishment of naive pluripotency. *Nat. Commun.* **3**, 817 (2012).

60. Y. Tang, Y. Luo, Z. Jiang, Y. Ma, C.-J. Lin, C. Kim, M. G. Carter, T. Amano, J. Park, S. Kish, X. C. Tian, Jak/Stat3 signaling promotes somatic cell reprogramming by epigenetic regulation. *Stem Cells* **30**, 2645–2656 (2012).

61. J. Silva, J. Nichols, T. W. Theunissen, G. Guo, A. L. van Oosten, O. Barrandon, J. Wray, S. Yamanaka, I. Chambers, A. Smith, Nanog is the gateway to the pluripotent ground state. *Cell* **138**, 722–737 (2009).

62. G. Csankovszki, A. Nagy, R. Jaenisch, Synergism of Xist RNA, DNA methylation, and histone hypoacetylation in maintaining X chromosome inactivation. *J. Cell Biol.* **153**, 773–784 (2001).

63. Y. Gao, J. Chen, K. Li, T. Wu, B. Huang, W. Liu, X. Kou, Y. Zhang, H. Huang, Y. Jiang, C. Yao, X. Liu, Z. Lu, Z. Xu, L. Kang, J. Chen, H. Wang, T. Cai, S. Gao, Replacement of Oct4 by Tet1 during iPSC induction reveals an important role of DNA methylation and hydroxymethylation in reprogramming. *Cell Stem Cell* **12**, 453–469 (2013).

64. S. Kienhöfer, M. U. Musheev, U. Stapf, M. Helm, L. Schomacher, C. Niehrs, A. Schäfer, GADD45a physically and functionally interacts with TET1. *Differentiation* **90**, 59–68 (2015).

65. B. R. Voldborg, L. Damstrup, M. Spang-Thomsen, H. Skovgaard Poulsen, Epidermal growth factor receptor (EGFR) and EGFR mutations, function and possible role in clinical trials. *Ann. Oncol.* **8**, 1197–1206 (1997).

66. S.-H. Yang, T. Kalkan, C. Morrisroe, A. Smith, A. D. Sharrocks, A genome-wide RNAi screen reveals MAP kinase phosphatases as key ERK pathway regulators during embryonic stem cell differentiation. *PLOS Genet.* **8**, e1003112 (2012).

67. S. Augui, E. P. Nora, E. Heard, Regulation of X-chromosome inactivation by the X-inactivation centre. *Nat. Rev. Genet.* **12**, 429–442 (2011).

68. J. Boeren, J. Gribnau, Xist-mediated chromatin changes that establish silencing of an entire X chromosome in mammals. *Curr. Opin. Cell Biol.* **70**, 44–50 (2021).

69. I. Zvetkova, A. Apedaile, B. Ramsahoye, J. E. Mermoud, L. A. Crompton, R. John, R. Feil, N. Brockdorff, Global hypomethylation of the genome in XX embryonic stem cells. *Nat. Genet.* **37**, 1274–1279 (2005).

70. V. Pasque, R. Karnik, C. Chronis, P. Petrella, J. Langerman, G. Bonora, J. Song, L. Vanheer, A. Sadhu Dimashkie, A. Meissner, K. Plath, X chromosome dosage influences DNA methylation dynamics during reprogramming to mouse iPSCs. *Stem Cell Rep.* **10**, 1537–1550 (2018).

71. W. Zhou, T. Hinoue, B. Barnes, O. Mitchell, W. Iqbal, S. M. Lee, K. K. Foy, K.-H. Lee, E. J. Moyer, A. VanderArk, J. M. Koeman, W. Ding, M. Kalkat, N. J. Spix, B. Eagleson, J. A. Pospisilik, P. E. Szabó, M. S. Bartolomei, N. A. Vander Schaaf, L. Kang, A. K. Wiseman, P. A. Jones, C. M. Krawczyk, M. Adams, R. Porecha, B. H. Chen, H. Shen, P. W. Laird, DNA methylation dynamics and dysregulation delineated by high-throughput profiling in the mouse. *Cell Genom.* **2** 100144 (2022).

72. X. Wu, Y. Zhang, TET-mediated active DNA demethylation: Mechanism, function and beyond. *Nat. Rev. Genet.* **18**, 517–534 (2017).

73. Y. Costa, J. Ding, T. W. Theunissen, F. Faiola, T. A. Hore, P. V. Shliaha, M. Fidalgo, A. Saunders, M. Lawrence, S. Dietmann, S. Das, D. N. Levasseur, Z. Li, M. Xu, W. Reik, J. C. R. Silva, J. Wang, NANOG-dependent function of TET1 and TET2 in establishment of pluripotency. *Nature* **495**, 370–374 (2013).

74. N. Okashita, Y. Kumaki, K. Ebi, M. Nishi, Y. Okamoto, M. Nakayama, S. Hashimoto, T. Nakamura, K. Sugasawa, N. Kojima, T. Takada, M. Okano, Y. Seki, PRDM14 promotes active DNA demethylation

through the ten-eleven translocation (TET)-mediated base excision repair pathway in embryonic stem cells. *Development* **141**, 269–280 (2014).

75. M. Weber, I. Hellmann, M. B. Stadler, L. Ramos, S. Pääbo, M. Rebhan, D. Schübeler, Distribution, silencing potential and evolutionary impact of promoter DNA methylation in the human genome. *Nat. Genet.* **39**, 457–466 (2007).

76. C. A. Doege, K. Inoue, T. Yamashita, D. B. Rhee, S. Travis, R. Fujita, P. Guarnieri, G. Bhagat, W. B. Vanti, A. Shih, R. L. Levine, S. Nik, E. I. Chen, A. Abeliovich, Early-stage epigenetic modification during somatic cell reprogramming by Parp1 and Tet2. *Nature* **488**, 652–655 (2012).

77. J. L. Sardina, S. Collombet, T. V. Tian, A. Gómez, B. Di Stefano, C. Berenguer, J. Brumbaugh, R. Stadhouders, C. Segura-Morales, M. Gut, I. G. Gut, S. Heath, S. Aranda, L. Di Croce, K. Hochedlinger, D. Thieffry, T. Graf, Transcription factors drive Tet2-mediated enhancer demethylation to reprogram cell fate. *Cell Stem Cell* **23**, 905–906 (2018).

78. M. A. Esteban, T. Wang, B. Qin, J. Yang, D. Qin, J. Cai, W. Li, Z. Weng, J. Chen, S. Ni, K. Chen, Y. Li, X. Liu, J. Xu, S. Zhang, F. Li, W. He, K. Labuda, Y. Song, A. Peterbauer, S. Wolbank, H. Redl, M. Zhong, D. Cai, L. Zeng, D. Pei, Vitamin C enhances the generation of mouse and human induced pluripotent stem cells. *Cell Stem Cell* **6**, 71–79 (2010).

79. R. Yin, S.-Q. Mao, B. Zhao, Z. Chong, Y. Yang, C. Zhao, D. Zhang, H. Huang, J. Gao, Z. Li, Y. Jiao, C. Li, S. Liu, D. Wu, W. Gu, Y.-G. Yang, G.-L. Xu, H. Wang, Ascorbic acid enhances Tet-mediated 5-methylcytosine oxidation and promotes DNA demethylation in mammals. *J. Am. Chem. Soc.* **135**, 10396–10403 (2013).

80. J. Chen, L. Guo, L. Zhang, H. Wu, J. Yang, H. Liu, X. Wang, X. Hu, T. Gu, Z. Zhou, J. Liu, J. Liu, H. Wu, S.-Q. Mao, K. Mo, Y. Li, K. Lai, J. Qi, H. Yao, G. Pan, G.-L. Xu, D. Pei, Vitamin C modulates TET1 function during somatic cell reprogramming. *Nat. Genet.* **45**, 1504–1509 (2013).

81. G. N. L. Chua, K. L. Wassarman, H. Sun, J. A. Alp, E. I. Jarczyk, N. J. Kuzio, M. J. Bennett, B. G. Malachowsky, M. Kruse, A. J. Kennedy, Cytosine-based TET enzyme inhibitors. *ACS Med. Chem. Lett.* **10**, 180–185 (2019).

82. Q. Tong, E. V. Vassilieva, A. I. Ivanov, Z. Wang, G. T. Brown, C. A. Parkos, A. Nusrat, Interferon-gamma inhibits T84 epithelial cell migration by redirecting transcytosis of beta1 integrin from the migrating leading edge. *J. Immunol.* **175**, 4030–4038 (2005).

83. Y. Qing, G. R. Stark, Alternative activation of STAT1 and STAT3 in response to interferon-gamma. *J. Biol. Chem.* **279**, 41679–41685 (2004).

84. H. Niwa, T. Burdon, I. Chambers, A. Smith, Self-renewal of pluripotent embryonic stem cells is mediated via activation of STAT3. *Genes Dev.* **12**, 2048–2060 (1998).

85. X. Chen, H. Xu, P. Yuan, F. Fang, M. Huss, V. B. Vega, E. Wong, Y. L. Orlov, W. Zhang, J. Jiang, Y.-H. Loh, H. C. Yeo, Z. X. Yeo, V. Narang, K. R. Govindarajan, B. Leong, A. Shahab, Y. Ruan, G. Bourque, W.-K. Sung, N. D. Clarke, C.-L. Wei, H.-H. Ng, Integration of external signaling pathways with the core transcriptional network in embryonic stem cells. *Cell* **133**, 1106–1117 (2008).

86. B. Shi, D. Gao, L. Zhong, M. Zhi, X. Weng, J. Xu, J. Li, X. Du, Y. Xin, J. Gao, Q. Zhu, S. Cao, Z. Liu, J. Han, IRF-1 expressed in the inner cell mass of the porcine early blastocyst enhances the pluripotency of induced pluripotent stem cells. *Stem Cell Res. Ther.* **11**, 505 (2020).

87. M. E. Donohoe, S. S. Silva, S. F. Pinter, N. Xu, J. T. Lee, The pluripotency factor Oct4 interacts with Ctcf and also controls X-chromosome pairing and counting. *Nature* **460**, 128–132 (2009).

88. Z. Ma, T. Swigut, A. Valouev, A. Rada-Iglesias, J. Wysocka, Sequence-specific regulator Prdm14 safeguards mouse ESCs from entering extraembryonic endoderm fates. *Nat. Struct. Mol. Biol.* **18**, 120–127 (2011).

89. P. Navarro, A. Oldfield, J. Legoupi, N. Festuccia, A. Dubois, M. Attia, J. Schoorlemmer, C. Rougeulle, I. Chambers, P. Avner, Molecular coupling of Tsix regulation and pluripotency. *Nature* **468**, 457–460 (2010).

90. A. Minkovsky, A. Sahakyan, G. Bonora, R. Damoiseaux, E. Dimitrova, L. Rubbi, M. Pellegrini, C. G. Radu, K. Plath, A high-throughput screen of inactive X chromosome reactivation identifies the enhancement of DNA demethylation by 5-aza-2’-dC upon inhibition of ribonucleotide reductase. *Epigenetics Chromatin* **8**, 42 (2015).

91. D. Lessing, T. O. Dial, C. Wei, B. Payer, L. L. G. Carrette, B. Kesner, A. Szanto, A. Jadhav, D. J. Maloney, A. Simeonov, J. Theriault, T. Hasaka, A. Bedalov, M. S. Bartolomei, J. T. Lee, A high-throughput small molecule screen identifies synergism between DNA methylation and Aurora kinase pathways for X reactivation. *Proc. Natl. Acad. Sci. U.S.A.* **113**, 14366–14371 (2016).

92. T. A. Hore, F. von Meyenn, M. Ravichandran, M. Bachman, G. Ficz, D. Oxley, F. Santos, S. Balasubramanian, T. P. Jurkowski, W. Reik, Retinol and ascorbate drive erasure of epigenetic memory and enhance reprogramming to naïve pluripotency by complementary mechanisms. *Proc. Natl. Acad. Sci. U.S.A.* **113**, 12202–12207 (2016).

93. S. Kienhöfer, M. U. Musheev, U. Stapf, M. Helm, L. Schomacher, C. Niehrs, A. Schäfer, GADD45a physically and functionally interacts with TET1. *Differentiation* **90**, 59–68 (2015).

94. K. Arab, E. Karaulanov, M. Musheev, P. Trnka, A. Schäfer, I. Grummt, C. Niehrs, GADD45A binds R-loops and recruits TET1 to CpG island promoters. *Nat. Genet.* **51**, 217–223 (2019).

95. S. Ito, L. Shen, Q. Dai, S. C. Wu, L. B. Collins, J. A. Swenberg, C. He, Y. Zhang, Tet proteins can convert 5-methylcytosine to 5-formylcytosine and 5-carboxylcytosine. *Science* **333**, 1300–1303 (2011).

96. K. Blaschke, K. T. Ebata, M. M. Karimi, J. A. Zepeda-Martínez, P. Goyal, S. Mahapatra, A. Tam, D. J. Laird, M. Hirst, A. Rao, M. C. Lorincz, M. Ramalho-Santos, Vitamin C induces Tet-dependent DNA demethylation and a blastocyst-like state in ES cells. *Nature* **500**, 222–226 (2013).

97. T. Wang, K. Chen, X. Zeng, J. Yang, Y. Wu, X. Shi, B. Qin, L. Zeng, M. A. Esteban, G. Pan, D. Pei, The histone demethylases Jhdm1a/1b enhance somatic cell reprogramming in a vitamin-C-dependent manner. *Cell Stem Cell* **9**, 575–587 (2011).

98. K. T. Ebata, K. Mesh, S. Liu, M. Bilenky, A. Fekete, M. G. Acker, M. Hirst, B. A. Garcia, M. Ramalho-Santos, Vitamin C induces specific demethylation of H3K9me2 in mouse embryonic stem cells via Kdm3a/b. *Epigenetics Chromatin* **10**, 36 (2017).

99. J. Chen, H. Liu, J. Liu, J. Qi, B. Wei, J. Yang, H. Liang, Y. Chen, J. Chen, Y. Wu, L. Guo, J. Zhu, X. Zhao, T. Peng, Y. Zhang, S. Chen, X. Li, D. Li, T. Wang, D. Pei, H3K9 methylation is a barrier during somatic cell reprogramming into iPSCs. *Nat. Genet.* **45**, 34–42 (2013).

100. G. Liang, J. He, Y. Zhang, Kdm2b promotes induced pluripotent stem cell generation by facilitating gene activation early in reprogramming. *Nat. Cell Biol.* **14**, 457–466 (2012).

101. L. Beziaud, C. M. Young, A. M. Alonso, M. Norkin, A. R. Minafra, J. Huelsken, IFNγ-induced stem-like state of cancer cells as a driver of metastatic progression following immunotherapy. *Cell Stem Cell* **30**, 818–831.e6 (2023).

102. S. Mekhoubad, C. Bock, A. S. de Boer, E. Kiskinis, A. Meissner, K. Eggan, Erosion of dosage compensation impacts human iPSC disease modeling. *Cell Stem Cell* **10**, 595–609 (2012).

103. M. C. Anguera, R. Sadreyev, Z. Zhang, A. Szanto, B. Payer, S. D. Sheridan, S. Kwok, S. J. Haggarty, M. Sur, J. Alvarez, A. Gimelbrant, M. Mitalipova, J. E. Kirby, J. T. Lee, Molecular signatures of human induced pluripotent stem cells highlight sex differences and cancer genes. *Cell Stem Cell* **11**, 75–90 (2012).

104. S. Bar, L. R. Seaton, U. Weissbein, T. Eldar-Geva, N. Benvenisty, Global characterization of X chromosome inactivation in human pluripotent stem cells. *Cell Rep.* **27**, 20–29.e3 (2019).

105. M. Cloutier, S. Kumar, E. Buttigieg, L. Keller, B. Lee, A. Williams, S. Mojica-Perez, I. Erliandri, A. M. D. Rocha, K. Cadigan, G. D. Smith, S. Kalantry, Preventing erosion of X-chromosome inactivation in human embryonic stem cells. *Nat. Commun.* **13**, 2516 (2022).

106. E. J. Sousa, H. T. Stuart, L. E. Bates, M. Ghorbani, J. Nichols, S. Dietmann, J. C. R. Silva, Exit from naive pluripotency induces a transient X chromosome inactivation-like state in males. *Cell Stem Cell* **22**, 919–928.e6 (2018).

107. S. Sawamiphak, Z. Kontarakis, D. Y. R. Stainier, Interferon gamma signaling positively regulates hematopoietic stem cell emergence. *Dev. Cell* **31**, 640–653 (2014).

108. M. H. Lexberg, A. Taubner, I. Albrecht, I. Lepenies, A. Richter, T. Kamradt, A. Radbruch, H.-D. Chang, IFN-γ and IL-12 synergize to convert in vivo generated Th17 into Th1/Th17 cells. *Eur. J. Immunol.* **40**, 3017–3027 (2010).

109. Y. Delneste, P. Charbonnier, N. Herbault, G. Magistrelli, G. Caron, J.-Y. Bonnefoy, P. Jeannin, Interferon-gamma switches monocyte differentiation from dendritic cells to macrophages. *Blood* **101**, 143–150 (2003).

110. J. T. Lee, N. Lu, Targeted mutagenesis of Tsix leads to nonrandom X inactivation. *Cell* **99**, 47–57 (1999).

111. S. Luikenhuis, A. Wutz, R. Jaenisch, Antisense transcription through the Xist locus mediates Tsix function in embryonic stem cells. *Mol. Cell. Biol.* **21**, 8512–8520 (2001).

112. Y. Ogawa, B. K. Sun, J. T. Lee, Intersection of the RNA interference and X-inactivation pathways. *Science* **320**, 1336–1341 (2008).

113. F. Nakaki, K. Hayashi, H. Ohta, K. Kurimoto, Y. Yabuta, M. Saitou, Induction of mouse germ-cell fate by transcription factors in vitro. *Nature* **501**, 222–226 (2013).

114. N. E. Sanjana, O. Shalem, F. Zhang, Improved vectors and genome-wide libraries for CRISPR screening. *Nat. Methods* **11**, 783–784 (2014).

115. S. A. Stewart, D. M. Dykxhoorn, D. Palliser, H. Mizuno, E. Y. Yu, D. S. An, D. M. Sabatini, I. S. Y. Chen, W. C. Hahn, P. A. Sharp, R. A. Weinberg, C. D. Novina, Lentivirus-delivered stable gene silencing by RNAi in primary cells. *RNA* **9**, 493–501 (2003).

116. B. W. Stringer, B. W. Day, R. C. J. D'Souza, P. R. Jamieson, K. S. Ensbey, Z. C. Bruce, Y. C. Lim, K. Goasdoué, C. Offenhäuser, S. Akgül, S. Allan, T. Robertson, P. Lucas, G. Tollesson, S. Campbell, C. Winter, H. Do, A. Dobrovic, P.-L. Inglis, R. L. Jeffree, T. G. Johns, A. W. Boyd, A reference collection of patient-derived cell line and xenograft models of proneural, classical and mesenchymal glioblastoma. *Sci. Rep.* **9**, 4902 (2019).

117. D. G. Gibson, L. Young, R.-Y. Chuang, J. C. Venter, C. A. Hutchison 3rd, H. O. Smith, Enzymatic assembly of DNA molecules up to several hundred kilobases. *Nat. Methods* **6**, 343–345 (2009).

118. H. Koike-Yusa, Y. Li, E.-P. Tan, M. D. C. Velasco-Herrera, K. Yusa, Genome-wide recessive genetic screening in mammalian cells with a lentiviral CRISPR-guide RNA library. *Nat. Biotechnol.* **32**, 267–273 (2014).

119. W. Li, H. Xu, T. Xiao, L. Cong, M. I. Love, F. Zhang, R. A. Irizarry, J. S. Liu, M. Brown, X. S. Liu, MAGeCK enables robust identification of essential genes from genome-scale CRISPR/Cas9 knockout screens. *Genome Biol.* **15**, 554 (2014).

120. M. Stadtfeld, N. Maherali, M. Borkent, K. Hochedlinger, A reprogrammable mouse strain from gene-targeted embryonic stem cells. *Nat. Methods* **7**, 53–55 (2010).

121. A. K. Hadjantonakis, L. L. Cox, P. P. Tam, A. Nagy, An X-linked GFP transgene reveals unexpected paternal X-chromosome activity in trophoblastic giant cells of the mouse placenta. *Genesis* **29**, 133–140 (2001).

122. M. Hooper, K. Hardy, A. Handyside, S. Hunter, M. Monk, HPRT-deficient (Lesch-Nyhan) mouse embryos derived from germline colonization by cultured cells. *Nature* **326**, 292–295 (1987).

123. J. Schindelin, I. Arganda-Carreras, E. Frise, V. Kaynig, M. Longair, T. Pietzsch, S. Preibisch, C. Rueden, S. Saalfeld, B. Schmid, J.-Y. Tinevez, D. J. White, V. Hartenstein, K. Eliceiri, P. Tomancak, A. Cardona, Fiji: An open-source platform for biological-image analysis. *Nat. Methods* **9**, 676–682 (2012).

124. A. Dobin, C. A. Davis, F. Schlesinger, J. Drenkow, C. Zaleski, S. Jha, P. Batut, M. Chaisson, T. R. Gingeras, STAR: Ultrafast universal RNA-seq aligner. *Bioinformatics* **29**, 15–21 (2013).

125. B. van de Geijn, G. McVicker, Y. Gilad, J. K. Pritchard, WASP: Allele-specific software for robust molecular quantitative trait locus discovery. *Nat. Methods* **12**, 1061–1063 (2015).

126. S. Anders, P. T. Pyl, W. Huber, HTSeq—A Python framework to work with high-throughput sequencing data. *Bioinformatics* **31**, 166–169 (2015).

127. P. Di Tommaso, M. Chatzou, E. W. Floden, P. P. Barja, E. Palumbo, C. Notredame, Nextflow enables reproducible computational workflows. *Nat. Biotechnol.* **35**, 316–319 (2017).

128. M. I. Love, W. Huber, S. Anders, Moderated estimation of fold change and dispersion for RNA-seq data with DESeq2. *Genome Biol.* **15**, 550 (2014).

129. M. E. Ritchie, B. Phipson, D. Wu, Y. Hu, C. W. Law, W. Shi, G. K. Smyth, limma powers differential expression analyses for RNA-sequencing and microarray studies. *Nucleic Acids Res.* **43**, e47 (2015).

130. G. Yu, L.-G. Wang, Q.-Y. He, ChIPseeker: An R/Bioconductor package for ChIP peak annotation, comparison and visualization. *Bioinformatics* **31**, 2382–2383 (2015).

131. W. Zhou, T. J. Triche Jr, P. W. Laird, H. Shen, SeSAMe: Reducing artifactual detection of DNA methylation by Infinium BeadChips in genomic deletions. *Nucleic Acids Res.* **46**, e123 (2018).

132. G. G. Chen, J. A. Gross, P.-E. Lutz, K. Vaillancourt, G. Maussion, A. Bramoulle, J.-F. Théroux, E. S. Gardini, U. Ehlert, G. Bourret, A. Masurel, P. Lepage, N. Mechawar, G. Turecki, C. Ernst, Medium throughput bisulfite sequencing for accurate detection of 5-methylcytosine and 5-hydroxymethylcytosine. *BMC Genomics* **18**, 96 (2017).

133. E. Leitão, J. Beygo, M. Zeschnigk, L. Klein-Hitpass, M. Bargull, S. Rahmann, B. Horsthemke, Locus-specific DNA methylation analysis by targeted deep bisulfite sequencing. *Methods Mol. Biol.* **1767**, 351–366 (2018).

134. L.-C. Li, R. Dahiya, MethPrimer: Designing primers for methylation PCRs. *Bioinformatics* **18**, 1427–1431 (2002).

135. O. Shalem, N. E. Sanjana, E. Hartenian, X. Shi, D. A. Scott, T. S. Mikkelsen, D. Heckl, B. L. Ebert, D. E. Root, J. G. Doench, F. Zhang, Genome-scale CRISPR-Cas9 knockout screening in human cells. *Science* **343**, 84–87 (2014).

136. L. Guo, L. Lin, X. Wang, M. Gao, S. Cao, Y. Mai, F. Wu, J. Kuang, H. Liu, J. Yang, S. Chu, H. Song, D. Li, Y. Liu, K. Wu, J. Liu, J. Wang, G. Pan, A. P. Hutchins, J. Liu, D. Pei, J. Chen, Resolving cell fate decisions during somatic cell reprogramming by single-cell RNA-seq. *Mol. Cell* **73**, 815–829.e7 (2019).

137. R. Montalbán-Loro, A. Lozano-Ureña, M. Ito, C. Krueger, W. Reik, A. C. Ferguson-Smith, S. R. Ferrón, TET3 prevents terminal differentiation of adult NSCs by a non-catalytic action at Snrpn. *Nat. Commun.* **10**, 1726 (2019).

138. K. Kurimoto, Y. Yabuta, Y. Ohinata, Y. Ono, K. D. Uno, R. G. Yamada, H. R. Ueda, M. Saitou, An improved single-cell cDNA amplification method for efficient high-density oligonucleotide microarray analysis. *Nucleic Acids Res.* **34**, e42 (2006).
